# Efficacy and safety of inhaled heparin in asthmatic and chronic obstructive pulmonary disease patients: a systematic review and a meta-analysis

**DOI:** 10.1038/s41598-023-40489-8

**Published:** 2023-08-16

**Authors:** Rasha Ashmawy, Adel Zaki, Ayman Baess, Iman El Sayed

**Affiliations:** 1 Department of Clinical Research, Maamora Chest Hospital, MoHP, Alexandria, Egypt; 2 Department of Biomedical Informatics and Medical Statistics, Medical Research Institute, Alexandria University, Alexandria, Egypt; 3 Department of Chest Diseases, Faculty of Medicine, Alexandria University, Alexandria, Egypt

**Keywords:** Diseases, Respiratory tract diseases

## Abstract

Asthma and chronic obstructive pulmonary disease (COPD) are prevalent chronic respiratory disorders that cause significant morbidity and mortality. Some studies evaluated the use of inhaled unfractionated heparin (UFH) in the treatment of asthma and COPD. We aimed to synthesize the available evidence for the efficacy and safety of inhaled heparin in improving lung functions among asthmatic and COPD patients. A comprehensive search was performed using Pubmed, Embase, EBSCO, Scopus, Web of Science, Cochrane CENTRAL, WHO Clinical trials, clinicaltrials.gov, Iranian Clinical trials, Google Scholar, Research Gate, ProQuest Thesis, OVID, and medRxiv databases. Two independent reviewers included all pertinent articles according to PRISMA guidelines, and extract data independently. The two reviewers checked the quality of studies using the ROB2 tool. To determine the pooled effect estimate of the efficacy and safety of inhaled heparin, a meta-analysis was carried out using the R programming language. Publication bias was evaluated using Egger’s regression test. The heterogeneity was explained using a meta-regression, and the quality of evidence was assessed by the GRADE approach. Twenty-six studies with a total of 581 patients were included in the qualitative analysis and 16 in the meta-analysis. The primary outcome was treatment success (improvement of lung function) that was measured by standardized mean differences (SMD) of the forced expiratory volume per second (FEV1) either per ml or percentage. Heparin has a large effect on both FEV1% and FEV1 ml when compared to the control group (SMD 2.7, 95% CI 1.00; 4.39; GRADE high, SMD 2.12, 95% CI − 1.49; 5.72: GRADE moderate, respectively). Secondary outcomes are other lung functions improving parameters such as PC20 (SMD 0.91, 95% CI − 0.15; 1.96). Meta-regression and subgroup analysis show that heparin type, dose, year of publication, study design, and quality of studies had a substantial effect. Regarding safety, inhaled heparin showed a good coagulation profile and mild tolerable side effects. Inhaled heparin showed improvement in lung functions either alone or when added to standard care. More large parallel RCTs are needed including COPD patients, children, and other types, and stages of asthmatic patients.

## Introduction

Asthma and chronic obstructive pulmonary disease (COPD) are prevalent chronic respiratory disorders that cause significant morbidity and mortality rates in primary care. Both conditions involve obstructive airflow limitation and inflammation and are classified within the same spectrum of pulmonary disorders^1^. If left untreated, asthma may be a risk factor for the development of chronic airflow limitation and COPD^2^. It is noteworthy that approximately 300 million people, or 4.3% of the world’s population, suffer from asthma^3,4^. Moreover, since 2001, the prevalence of asthma has been increasing by 2.9% annually^5^. Additionally, it is anticipated that the incidence of COPD will increase in the next three decades, with annual deaths attributed to COPD and related conditions projected to reach 4.5 million by 2030^6^. It is crucial to address these conditions and implement appropriate measures to improve patients’ quality of life and reduce mortality rates^6^.

The classification of asthma as a TH2 process is strongly linked to atopy and allergy^7^, as well as the characteristics of COPD, which involves multiple types of inflammatory cells emitting various inflammatory mediators^8^. The cornerstone of treatment for both conditions is anti-inflammatory drugs, especially corticosteroids, and bronchodilators with different mechanisms of action. However, these treatments have limitations, such as the nonspecific effects and serious adverse events associated with corticosteroids, and the inability of bronchodilators to treat underlying inflammation^9–11^. Individuals who have severe asthma, or COPD, or are smokers may also develop steroid resistance, which is a major barrier to effective therapy. New anti-inflammatory drugs like oral phosphodiesterase inhibitors have been developed, but due to their systemic side effects leading to search for drugs that are effective through inhaled delivery^12^. Thus, a new steroid-free combination therapy with potent anti-inflammatory activities and continuous release properties would address some of the limitations of current practice in asthma and COPD therapy^13^.

Unfractionated Heparin (UFH) is a well-known drug with both anticoagulant and anti-inflammatory properties. In recent years, inhaled heparin regimens have gained popularity in the management of pulmonary diseases, including cystic fibrosis, thromboembolism, COPD, pulmonary fibrosis, bronchial asthma, and asthma-induced airway hypersensitivity^14^. The interconnectedness of inflammation, thrombogenesis, atherogenesis, and cell proliferation implies that drugs such as heparin and its derivatives that have multiple effects (pleiotropic) may have greater therapeutic potential than compounds that only target one pathway. Remarkably, low molecular weight heparin (LMWH) enoxaparin significantly reduced eosinophilic and lymphocytic counts in bronchoalveolar lavage (BAL) samples, without any significant change in IL-5 or Eosinophil Cationic Protein (ECP) concentrations^15^.

Although both asthma and COPD share characteristics of chronic airway inflammation, excessive mucus production, and restricted airflow. In asthma, mast cell activation leads to reversible airflow blockage^16^, while in COPD, irreversible limitation is caused by factors like small airway inflammation, fibrosis, emphysema, and oxidative stress. Asthma exacerbations are triggered by rhinovirus infections and allergens^17^, while COPD exacerbations arise from viral or bacterial infections, each displaying distinct inflammation patterns. Asthma involves eosinophilic and neutrophilic inflammation, whereas COPD is associated with increased macrophages, neutrophils, and immune cells^18^. Oxidative stress contributes to inflammation in both conditions. Epithelial cell dysfunction, excessive mucus production, and the activity of neutrophil elastase exacerbate asthma and COPD^19^. Whereas neutrophil elastase impairs immune responses, mucus clearance, and causes tissue damage. Heparin, with its anti-inflammatory effects, hinders neutrophil activation and recruitment, platelet interactions, and heparanase activity, influencing lung tissue inflammation^20^. Heparin’s longer saccharide chains also inhibit neutrophil elastase and cathepsin G, potentially aiding mucociliary clearance, restoring antiprotease balance, and reducing tissue damage^21^. Heparin’s antioxidant property may further alleviate inflammation, shield antiproteases, and limit mucus overproduction driven by reactive oxygen species. In cases of COPD and severe asthma, decreased Histone deacetylase 2 (HDAC2) impairs the effectiveness of corticosteroids, which antioxidants like heparin could potentially ameliorate. Elevated eosinophil cationic proteins in asthma contribute to airway dysfunction; heparin’s charge neutralization and inhibition of eosinophil effects might hold therapeutic promise^22^.

During the early 1960s, a number of studies examined the potential use of inhaled UFH in treating bronchoconstriction associated with asthma and COPD^13–15^. Although the exact mechanism by which heparin offers protection is not yet fully understood, it remains an area of continued interest in vivo asthma experimental models, such as those based on allergen-induced acute bronchoconstriction. Moreover, the effects of heparin on eosinophils and mast cell infiltration in guinea pigs and sheep are still being investigated^23^. Another review article has recommended evaluating the therapeutic efficacy of inhaled heparin in asthma, both in vivo and in vitro. Furthermore, this review has suggested that the anti-inflammatory properties of heparin are dose-dependent and influenced by the route of administration and molecular weight of the heparin^23^.

Previous systematic reviews have indicated that the anti-inflammatory effects of heparin are not specific to inhaled heparin use in asthma and COPD^15,24^. However, it is important to note that the majority of heparin formulations used for COPD patients are LMWH administered via injection, which can lead to significant improvements in lung function but may increase the risk of bleeding for these patients^25^. This study represents the first instance of a systematic review and meta-analysis focused on evaluating the effectiveness and acceptability of inhaled heparin and its derivatives as either an alternative or supplementary treatment option for COPD and asthma. This study aims to consolidate existing evidence on the efficacy and safety of inhaled heparin for improving lung function in patients with asthma and COPD.

## Methodology

### Registration of the study

The study protocol was registered in the PROSPERO database (CRD42020163992). This study was performed in concordance with PRISMA guidelines^26^, and adherence to the Cochrane Handbook of Systematic Reviews and Meta-analysis (Version 6.3.0)^27^.

### Criteria for considering studies for this meta-analysis

#### Types of studies

We included clinical trials, either parallel or crossover designs, to assess the beneficial effects of the treatments. No observational trials were found. We excluded case reports and case series studies. We have searched for trials in the English language only with no publication date restriction imposed.

#### Types of participants

Inclusion:

1. All asthmatic or COPD patients regardless of the previous treatment.
2. All COPD patients at any stage (stages according to GOLD guidelines).
3. Patients with any type of asthma (e.g. exercise-induced asthma “EIA”, atopic,… etc.).
4. Patient with different asthma or COPD severity (e.g. mild, moderate, severe, or critically ill).
5. Asthma simulation by bronchial provocation test.

Exclusion:

1. Other lung diseases (e.g. cystic fibrosis, IPF, lung cancer).
2. Asthma or COPD if combined with other respiratory disorders or infectious diseases.

#### Types of interventions

Inhaled heparin or its derivatives (low molecular weight heparins LMWHs), we retrieved dose, duration, and delivery from available studies. As some clinicians use injectable forms of heparin as an inhalation for some respiratory diseases to minimize its side effects.

#### Types of comparator(s)/control

Placebo or standard treatment (short-acting B2 agonist (SABA), short-acting antimuscarinic (SAMA), inhaled corticosteroids (ICS), long-acting B2 agonist (LABA), long-acting antimuscarinic (LAMA), leukotriene modulators or other asthma or COPD treatment as mentioned in the guidelines).

#### Types of outcome measures

##### Primary outcome

Treatment success (improvement of lung function) that measured by differences in the forced expiratory volume per second (FEV1) either per ml or percentage (FEV1 ml or FEV1%).

##### Secondary outcome(s)

1. Other pulmonary function improvement indicators: PC20% change for asthmatic patients (amount of allergen percentage increase from initial to the final concentration that causes 20% decrease in FEV1), PEFR, AUC, FEV1/FVC ratio.
2. Measures of adverse effects, e.g., effect on coagulation profile (either no effect or has an undesirable effect), Incidence of bleeding, the severity of bleeding; if present (minor or major).
3. Airway inflammation improvement percentage, e.g., eosinophil %, neutrophils % or lymphocytes either in sputum or bronchoalveolar lavage, Fractional Exhaled Nitric Oxide (FeNO%), C-Reactive Protein (CRP%).

### Search strategy for identification of studies

#### Electronic search

We conducted a comprehensive literature search from January to March 2021 to identify all published and unpublished trials with English language restrictions and no publication date imposed. We have searched the following electronic databases to identify potential studies:

- MEDLINE (PubMed), CENTRAL (The Cochrane Central Register of Controlled Trials), Scopus, and Ovid.
- The search strategy was performed by 2 independent reviewers including only terms relating to or describing the intervention.
- Potential search terms are (Asthma OR COPD OR “Chronic obstructive pulmonary disease” bronchoconstriction OR “lung hyperreactivity” OR “pulmonary obstruction”) AND (heparin OR UFH OR LMWH OR anticoagulants).
- Before completion of the review, an updated search was done in December 2022 to check the literature again to ensure not missing any relevant studies.

Supplementary Appendix 1 shows a detailed search strategy for each database with appropriate search terms.

#### Searching other literature sources

- Clinical trials registries e.g., Clinicaltrials.gov, who.int/trial search.
- We checked the reference lists of all relevant studies and review articles for additional references.
- We searched relevant grey literature sources such as the Web of Science core collection (WOS), reports, dissertations, theses, and relevant journals to the condition.
- We searched within previous reviews on the same topic.
- We contacted relevant individuals and organizations for information about unpublished or ongoing studies.

### Data collection and analysis

#### Selection of studies

Two independent reviewers scanned the title, abstract, or both, of every retrieved record, to determine which studies should be assessed further. We investigated all potentially relevant articles as full text, and a third reviewer to resolve any discrepancies. The reviewers recorded the selection process in enough detail to complete a PRISMA (Preferred Reporting Items for Systematic Reviews and Meta-Analyses)^26^, flow diagram for “characteristics for study selection”, “Characteristics of excluded studies” and “studies awaiting classification”.

### Data extraction and management

We extracted the data from the eligible studies by 2 independent reviewers guided by the Cochrane data extraction form to populate a table of Characteristics of included studies.

The following data were extracted:

1. Study characteristics (first author, publication year, study design, the sample size of participants, funding for studies, and notable conflicts of interest of trial authors).
2. Pharmacotherapy: intervention (nebulized heparin or LMWH with dose, timing, and duration according to each study), comparison & concomitant medications.
3. Participants: age, gender, lung functions of study participants, wash-out period.
4. Outcomes (primary outcomes and secondary outcomes).

### Assessment of methodological quality of included studies

Cochrane Risk-of-Bias tool (ROB2) was used to assess the methodological quality of included studies, it assesses different types of bias in five domains^28^.

RiOB2 was independently assessed by two reviewers and one of the third reviewer resolved disagreements. We judged ‘Risk of bias criteria’ as ‘low risk’, ‘high risk’ or ‘unclear risk’ and evaluate individual bias items as described in the Cochrane Handbook for Systematic Reviews of Interventions^27^. We provided a quote from the study report together with a justification for our judgment in the “Risk of bias” table. We presented a “Risk of bias” graph and a ‘Risk of bias summary’ figure. Finally, we summarize the overall quality of the meta-analysis included studies according to ROB2 into high, some concerns, or low. In addition to Agency of health care research and quality (AHRQ) recommendations into good, fair, or poor^29^.

### Measures of treatment effect

We calculated the mean differences (MD) and standardized mean differences (SMD) of continuous outcome data, with respective 95% confidence intervals (CIs), no categorical estimates were used. We performed a meta-analysis and displayed forest plots to show individual studies and meta-analysis estimates. Then, we considered the magnitude of the effect according to Cohen’s d scale^30^; SMD more than zero means favoring the treatment side than placebo, if SMD values 0.2–0.5 it is considered a small effect or contribution, 0.5–0.8 medium, and > 0.8 considered a large effect.

### Unit of analysis issues

We considered the level at which randomization occurred, such as cross-over trials, cluster-randomized trials, and multiple observations for the same outcome. In order to decrease the unit of analysis error in a crossover design^31^, we incorporated the second approach for reporting the outcome by including the data from the first period^32^.

### Dealing with missing data

We detected missing data in any included study, and to get the information we contacted the study’s corresponding author to verify key study characteristics and obtain missing numerical outcome data where possible when the study is identified as an abstract only, and if no response for 6 weeks we conducted the available case analysis.

### Assessment of heterogeneity

Heterogeneity was assessed by visual inspection of the forest plot, Cochrane’s Q test at a significance level α = 0.1, and Higgin’s I^2^ statistics a useful statistic for quantifying inconsistency which can be interpreted as follows:

- 0% to 40%: might not be important; 30% to 60%: may represent moderate heterogeneity; 50% to 90%: may represent substantial heterogeneity and 75% to 100%: considerable heterogeneity.

When we find heterogeneity, we attempted to determine potential reasons for it by examining individual studies or conducting subgroup analysis.

### Assessment for reporting bias

We assessed possible reporting bias on two levels: within‐study bias and between‐study bias.

We examined within‐study selective outcome reporting as part of the overall ‘Risk of bias’.

We created a funnel plot of effect estimates against standard errors (SEs) to assess possible between‐study reporting bias if we will include at least 10 studies in the review. We considered possible explanations if we note the asymmetry of the funnel plot.

### Data synthesis (meta-analysis)

We performed the fixed-effect model meta-analysis, but if Cochrane Q test P-value < 0.1 and Higgin’s I^2^ > 50% indicates a significant heterogeneity between studies, we performed a random-effects model. In addition, we performed statistical analyses using RavMen5.3 software^33^, and R 4.2.2. (2022-10-31). The packages used were (meta), (dmetar), (metafor), (ggplot2), and (gridExtra).

### Analysis of subgroups and investigation of heterogeneity

Subgroup analyses were conducted, to examine the source of the clinical heterogeneity among the studies, concerning the following factors:

1. Asthmatic or COPD patients.
2. Type of heparin used or type of treatment (either alone or add-on).
3. Type of provocation material used.
4. Frequency, dose, and timing of heparin.
5. Study quality and year of publication.

Then, we performed a meta-regression to assess the impact of these factors whenever possible (for outcomes included 5 studies at least).

### Sensitivity analysis (outlier and influential removal)

According to Harrer et al. recommendations^34^, we used a variety of techniques to identify outlier and influential studies and reduce the heterogeneity between studies, including:

1. Brute force approach, if a study’s confidence interval does not fit the confidence interval for the pooled effect, it should be regarded as an outlier.
2. We calculated different influence diagnostics. Therefore, we identified the studies that have the greatest overall impact on our meta-analysis estimate and determine whether or not this significant influence has a negative impact on the pooled effect^35^.
3. Baujat plots are diagnostic graphs to find papers that excessively add to the heterogeneity in a meta-analysis^36^. The graph displays the influence of each study on the pooled effect size and the contribution of each study to the overall heterogeneity on the horizontal axis and vertical axes, respectively.
4. Leave-One-Out sensitivity forest plot, a plot shows the overall effect of all meta-analyses that could be conducted using the leave-one-out method, then print in one forest plot sorted by the pooled effect size. Illustrating the recalculated different pooled effects and 95% CI with one study omitted each time.

### Summary of findings

We presented ‘Summary of findings’ tables results of data synthesized for the primary outcome according to The GRADE approach. We assessed the quality of evidence according to one of four grades, High, moderate, low, and very low, by applying GRADE recommendations^37^ and using GRADEpro.GDT software^38^.

### Ethical approval

This review was performed by relevant national guidelines and regulations.

## Results

### Literature search

Searching literature was conducted from January to March 2021 and updated in December 2022 (Supplementary Appendix 1, shows a detailed search strategy for each database with appropriate search terms).

### Description of the studies

#### Included studies

In addition to an up-to-date electronic search of thirteen databases, we performed manual searches on the reference lists of the included studies and verification of the search for finished studies of published protocols. This led to the discovery of a total of 7086 articles, which were then electronically examined for duplication using the Endnote tool and found to contain 1604 duplicates. The remaining 5482 articles were then extracted to an Excel file for additional title abstract screening, leaving only 40 articles for full-text screening.

**Figure 1:**
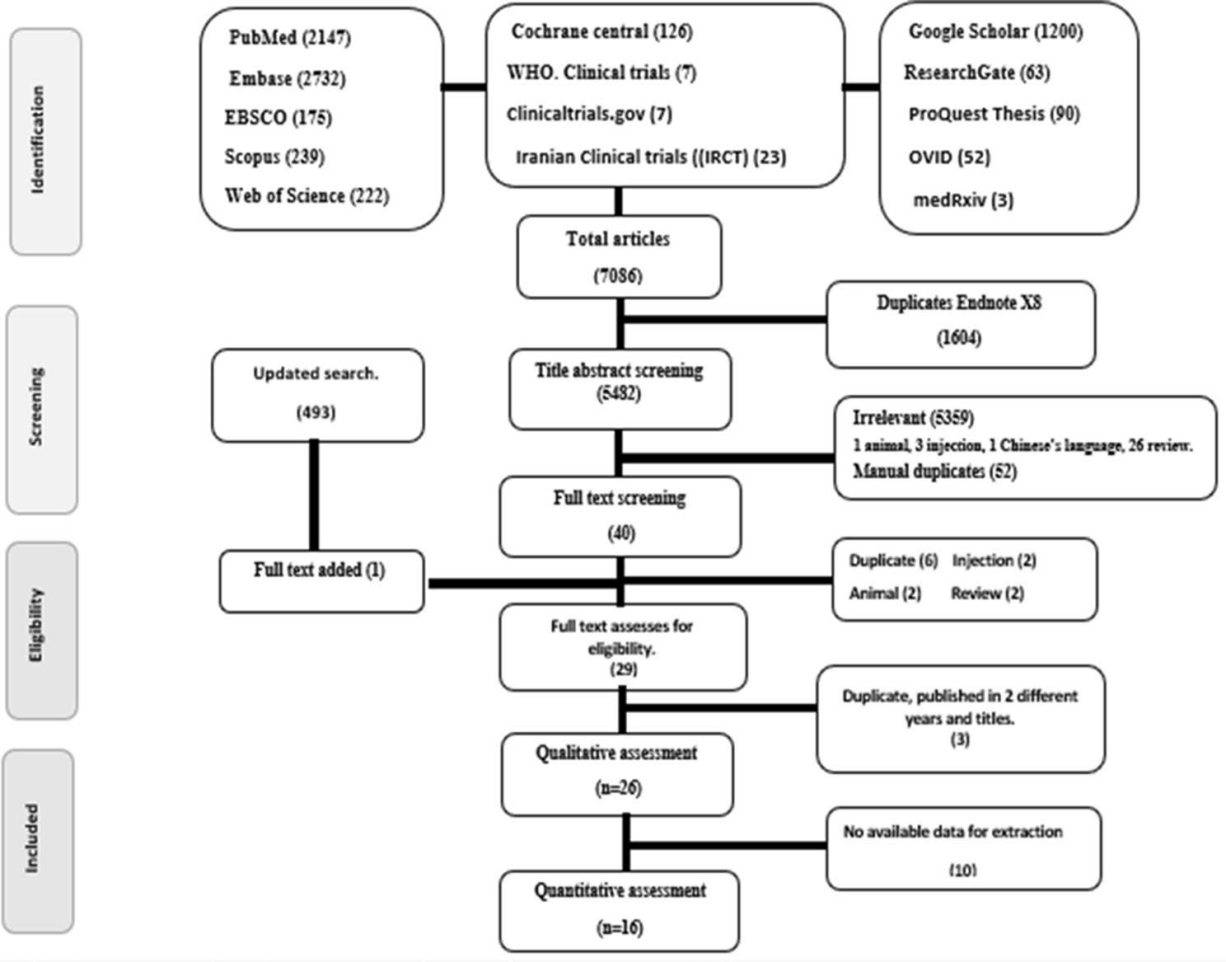
PRISMA flowchart of screened and included studies.

Finally, 26 articles with 581 participants were qualitatively retrieved (Supplementary Appendix 2). 23 articles discussed various types of asthma, two discussed COPD and one discussed the two illnesses. In terms of population, 22 publications on adults and 4 research on children were done (82 children included), Table 1 shows the summary of narrative synthesis. While 16 articles completed the meta-analysis process, Fig. 1 shows PRISMA flowchart.

**Table 1:** Summary of included articles for the narrative synthesis.

Author, Year, Country	Design	Population	Disease	Drug	Comparator	Outcomes	AHRQ	ROB2
Motamed, 2021, Iran^39^	RCT, parallel	Adult N = 70	Asthma Mild–Moderate	LMWH + Albuterol	Albuterol	FEV1, PEFR	Fair	S
Ashoor, 2020, Egypt^40^	RCT, parallel	Adults N = 60	COPD, MV stage II–IV	Heparin + Albuterol	Albuterol	Vent. days, PaCO2, PaO2/FiO2, CRP, APTT	Fair	S
Shute, 2017, UK^41^	RCT, parallel	Adults N = 40	COPD Mod–Severe	UFH + Albuterol + Beclomethasone	Placebo + Albuterol + Beclomethasone	FEV1, FVC, 6MWD	Poor	H
Duong, 2008, Canada^42^	RCT, crossover	Adults N = 18	Asthma Mild atopic	IVX-0142 Heparin derived	Placebo	FEV1, PC20	Poor	H
Abd-Elaty, 2007, Egypt^43^*	RCT, parallel	Adults N = 30	Asthma ex Mod–Severe	UFH + Albuterol + I.V. Hydrocortison	Placebo + Albuterol + I.V. Hydrocortison	Oxygen saturation, RR, No. of Albuterol inhalation needed	NA	NA
Lui, 2006, China^44^*	RCT	Adults N = 40	Asthma Mild–Mod.	UFH	Placebo	FEV1, FVC, PEFR, PC20 (Methacholine)	NA	NA
Fal, 2004, Poland^45^*	Quasi	Adults N = 24	Asthma	LMWH + standard medications	Standard medications	FEV1, Eso, lymph, EG2, SVCAM1, IL-5, ECP	NA	NA
Stelmach, 2003, Poland^46^*	RCT, crossover	Children N = 23	Asthma Mild allergic	UFH	Placebo	PC20 (histamine or leukotriene D4)	NA	NA
Muszyńska, 2002, Poland^47^*	Quasi	Adults N = 17	Asthma	LMWH	–	BALF, Inflammatory cells	NA	NA
Stelmach, 2002, Poland^48^*	RCT, crossover	Children N = 14	Asthma Mild	UFH	Placebo	PC20 (leukotriene D3)	NA	NA
Stelmach, 2001, Poland^49^*	RCT, crossover	Children N = 15	Asthma Mild atopic	UFH	Placebo	PC20 (methacholine)	NA	NA
Tutluoglu, 2001, Turkey^50^	RCT, parallel	Adults N = 38	Asthma Allergic	UFH	Placebo	RFT, PC20 (KCL), EG2, IL-5, ECP	Fair	S
Ceyhan, 2000, Turkey^51^*	RCT, crossover	Adults N = 15	Asthma Mild	UFH or LMWH	Placebo	PC20 (Methacholine)	NA	NA
Kwasniewski, 2000^52^*	Not mentioned	Adults N = 21	Asthma and COPD	LMWH + SOC	SOC	Anti-X, APTT, platelets	NA	NA
Tranfa, 2000, Italy^53^	RCT, crossover	Adults N = 8	Asthma Atopic	UFH	Placebo	PC20 (UNDW)	Poor	H
Tahir, 1999, USA^54^	RCT, crossover	Adults N = 13	Adults EIA	UFH or LMWH	Placebo	FEV1	Fair	S
Lee, 1998, Korea*	RCT, crossover	Adults N = 8	Asthma EIA	UFH	Control	FEV1, PC20 (methacholine), APTT	NA	NA
Ceyhan, 1997, Turkey^55^*	RCT, crossover	Adults N = 15	Asthma Mild	UFH	Placebo	Geometric mean PDC20 (adenosine)	NA	NA
Kalpaklioglu, 1997, Turkey^56^	RCT, crossover	Adults N = 12	Asthma Mild	UFH	Placebo	PC20 (methacholine), raw, SGaw	Poor	H
Polosa, 1997, Italy^57^	RCT, parallel	Adults N = 17	Asthma Atopic	UFH	Placebo	FEV1, AMP produced bronchoconstriction	Good	L
Diamant, 1996, UK^58^	RCT, crossover	Adults N = 8	Asthma Mild–Mod.	UFH	Placebo	EAR.AUC, LAR. AUC	Good	L
Garrigo, 1996, USA^59^	RCT, crossover	Adults N = 9	Asthma EIA	UFH	Placebo	SGaw	Poor	H
Hong, 1996^60^*	RCT, crossover	Children N = 30	Asthma	UFH + standard ttt	Standard ttt	Effective treatment rate	NA	NA
Pavord, 1996, UK^61^	RCT, crossover	Adults N = 11	Asthma Mild	UFH	Placebo	FEV1, PC20 (Sod. metabisulphite), ATTP	Poor	H
Ceyhan, 1995, Turkey^62^	RCT, crossover	Adults N = 13	Asthma Mild	UFH	Placebo	PC20 (methacholine)	Poor	H
Ahmed, 1993, USA^63^	RCT, crossover	Adults N = 12	Asthma EIA	UFH	Placebo		Poor	H

*SOC* standard of care, *ttt* treatment, *ROB2* Risk of bias tool if *H* high, *S* some concerns, *L* low, *RCT* randomized control trial, *LMWH* low molecular weight heparin, *UFH* unfractionated heparin, *MV* mechanical ventilation.

*Abstract only.

### Quality assessment

**Figure 2:**
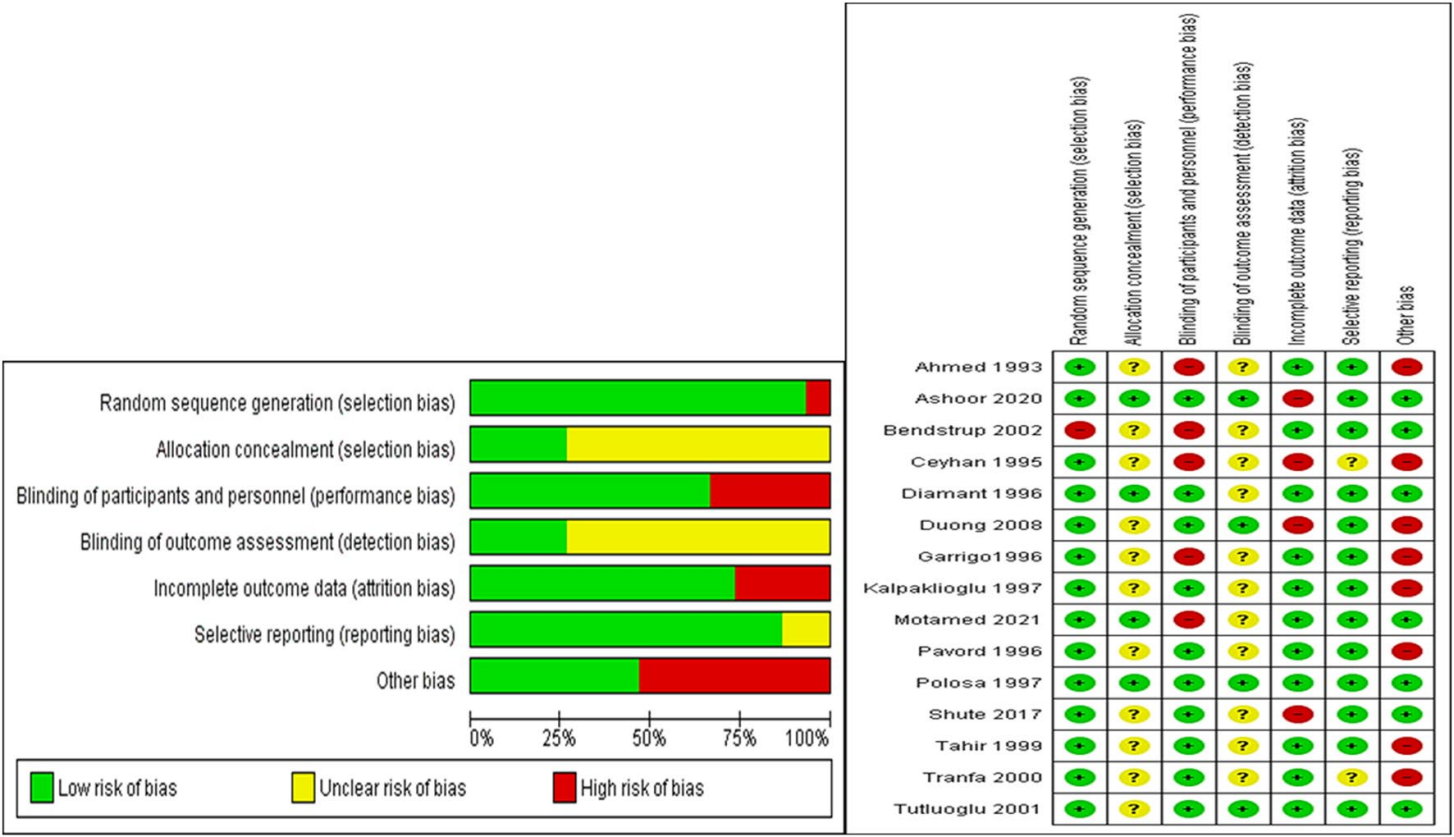
Quality assessment of the included studies in the meta-analysis.

Figure 2 shows the summary and individual quality of the included studies in this meta-analysis using the ROB2 tool, we found that most of the included studies had a low risk of selection bias (90% had random sequence), performance bias (70% had double blinding), attrition bias (70% had complete outcome data, and reporting bias (90% good reporting of outcomes), while nearly 75% of studies have an unclear risk of selection bias (allocation concealments not reported), and detection bias (blinding of the outcome, not Cleary mentioned), but due to the cross over design of 50% of studies they have a higher risk in other risks of bias.

### Primary outcome (lung function improvement indicator)

#### Forced expiratory volume at 1 s% (FEV1%)

**Figure 3:**
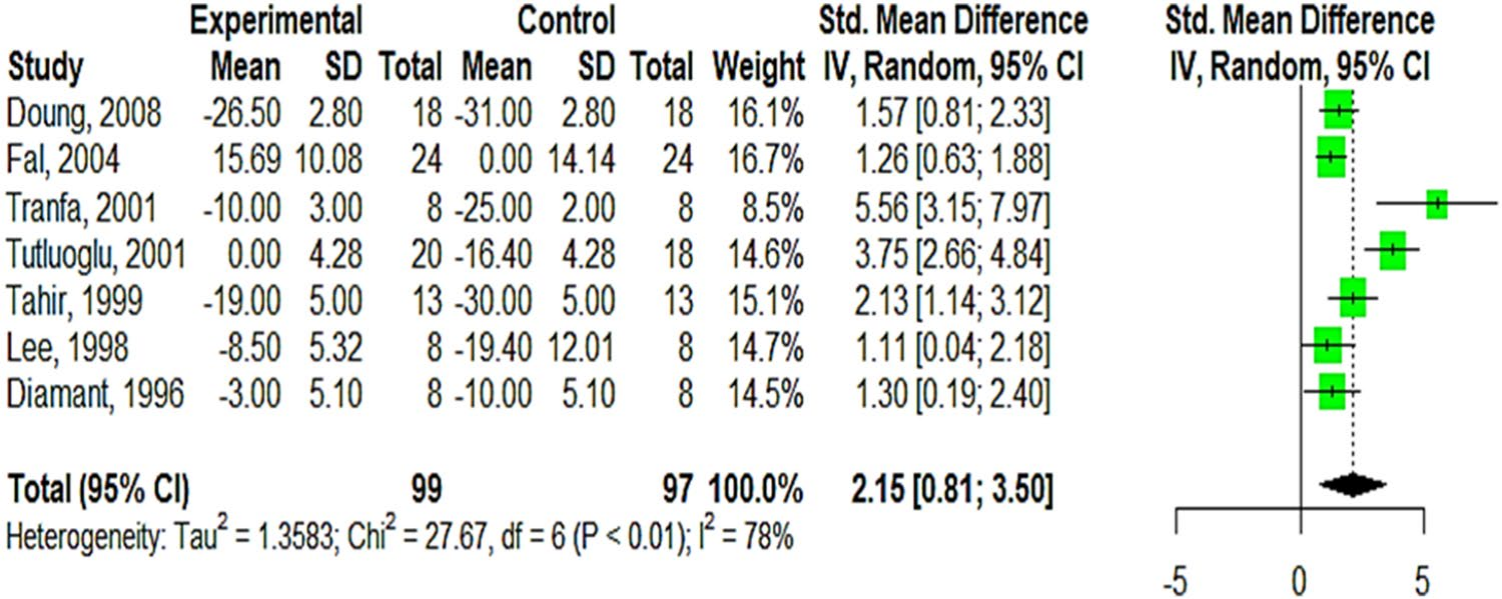
Forest plot showing pooled SMD of FEV1% after omitting Ahmed 1993 by leave-one sensitivity analysis.

Overall 8 studies were included in this meta-analysis with a total of 220 participants, all reported studies are for asthmatic patients. The pooled effect size SMD (Standard Mean Difference) of FEV1% was statistically significant at 2.7 (95% CI 1.00; 4.39, Tau^2^ = 3.2415, I^2^ = 85%, GRADE high), which means that using inhaled heparin make a large effect on FEV1% in asthmatic patients. Then, the Baujat plot was performed to check for outliers and influential causing heterogeneity, we found that Ahmed 1993, Tutuoglu 2001, and Tranfa 2001 are outliers regarding their contribution to heterogeneity (9 to > 15%), but we couldn’t consider them as influential due to their small sample size, (Supplementary Appendix 3, Fig. S1A–C). Figure 3 shows the SMD after performing a leave-one sensitivity analysis of Ahmed 1993, the result pooled effect slightly affected but still large and statistically significant SMD 2.15 (95% CI 0.81;3.50, Tau^2^ = 1.358) and heterogeneity decreased to I^2^ = 78%.

#### Subgroup analysis

Supplementary Appendix 2, Fig. S1E subgroup by heparin type shows that UFH is highly effective for improving FEV1% than LMWH (UFH SMD 3.23, 95% CI 0.88; 5.57, I2 = 86%, LMWH SMD 1.38, 95% CI − 0.58; 3.34, I2 = 0%). Figure S1F subgroup by provocation using allergen statistically significant difference for improving FEV1% than exercise (SMD 2.46, 95% CI 0.22; 4.70, I2 = 83%). With different bronchoconstriction inducers. Figure S1G,H,J,N show subgrouping by heparin time, frequency, dose respectively, and treatment type, where studies not mentioned heparin timing had the statically significant effect (SMD 1.22, 95% CI 0.41; 2.03, I^2^ = 0%), Using heparin once with a dose of 1000IU/Kg alone had the higher and statistically significant SMD 4.3 (95% CI 1.15; 7.45), SMD 3.5 (95% CI 0.47; 6.53), SMD 2.95 (95% CI 0.99; 4.9), respectively. Regarding study publication year category and quality, there was no statistically significant effect while for study design RCT cross-over had SMD 3.23 (95% CI 0.24; 6.22), Fig. S1K–M.

Besides Meta-regression was performed to detect the most predominant predictors of heterogeneity, predictors were the type of heparin and frequency (heparin used once a day for 5 days, ß =  − 3, 95% CI − 6.9; − 0.08), which can explain some of this heterogeneity (R^2^ = 34.85%).

#### Forced expiratory volume at 1 s FEV1 (ml)

**Figure 4:**
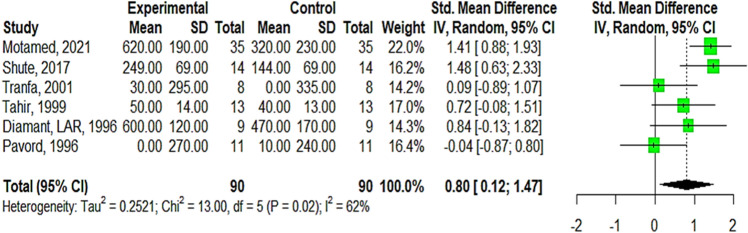
Forest plot illustrates the pooled SMD of FEV1 (ml) after performing leave-one sensitivity analysis, omit Tutuoglu 2001.

A total of 7 studies with 218 participants were included in this meta-analysis, all reported studies are for asthmatic patients except Shute for COPD patients. The pooled effect size (Standard Mean Difference) of FEV1 ml was statistically insignificant SMD 2.12 (95% CI − 1.49; 5.72, Tau^2^ = 13.48, I^2^ = 91%, GRADE Moderate), besides a substantial heterogeneity that affects the pooled estimate (Supplementary Appendix 2, Fig. S2A). Surprisingly after performing leave one sensitivity analysis by omitting Tuluoglu, 2001, the SMD become statistically significant with a large contribution to the outcome favoring the use of heparin SMD 0.8 (95% CI 0.12; 1.47, Tau^2^ = 0.252), in addition to a significant decrease in heterogeneity I^2^% = 62%, Fig. 4. The Baujat plot also confirms this as shown in (Supplementary Appendix 2, Fig. S2B), Tutuoglu is considered as an outlier and slightly influences the pooled effect.

#### Subgroup analysis

Supplementary Appendix 2, Fig. S2E,F subgroup by heparin and disease type shows no statistically significant difference, while Fig. S2F subgroup by provocation type shows that studies do not use allergen-induced bronchoconstriction had a statistically significant effect (SMD 1.43, 95% CI 1.02; 1.83, I^2^ = 0%). Figure S2H,J,K,O show subgrouping by heparin time, frequency, dose, and treatment type respectively, where subgroup analysis didn’t show any statistically significant difference pooled estimates except for studies not mentioning heparin timing had the statically significant effect and heparin as add on the drug (SMD 1.43, 95% CI 1.02; 1.83, I^2^ = 0%). Regarding study design and quality, there was no statistically significant effect while for the study publication year category (2015–2021) SMD 1.43 (95% CI 1.02; 1.83, I^2^ = 0%). Figure S2L–N.

Then, we conducted a meta-regression including study design and publication year category as predictors, and we found that 100% of the intra-studies heterogeneity contributed to study type either parallel or crossover (parallel, ß = 11.33, 95% CI 10.23; 12.4) and year of publication category (the publication year (2015–2021), ß =  − 10.67, 95% CI − 11.97; − 9.5, (2001–2005), ß =  − 0.68, 95% CI − 1.1; − 0.24, (1996–1999), ß =  − 0.8, CI − 1.2; − 0.4).

### Secondary outcomes

#### Other lung function improvement indicators

##### Forced vital capacity (FVC)

Supplementary Appendix 3, Fig. S4 shows that only 2 studies reported Forced Vital Capacity including 28 COPD and 38 asthmatic patients, in each study alone SMD has a high statistically significant effect favoring using inhaled heparin, but we couldn’t use pooled estimate due to large heterogeneity I^2^% = 96%. Shute 2017, Tultuoglu 2001 (SMD 1.22 and 11.42, 95% CI 0.4; 2.04 and 8.64; 14.2) respectively.

##### Peak expiratory flow rate (PEFR)

Similar to FVC, only 2 studies reported PEFR with 108 asthmatic participants but here one favors heparin use Tutuoglu 2001 (SMD 15.23, 95% CI 11.57; 18.89), while Motamed 2021 favors the placebo (SMD − 1.86, 95% CI − 2.43; − 1.3), I^2^% = 99%, (Supplementary Appendix 3, Fig. S5).

##### Provocation concentration of allergen causing 20% fall of FEV1% (PC20) for asthmatic patients

**Figure 5:**
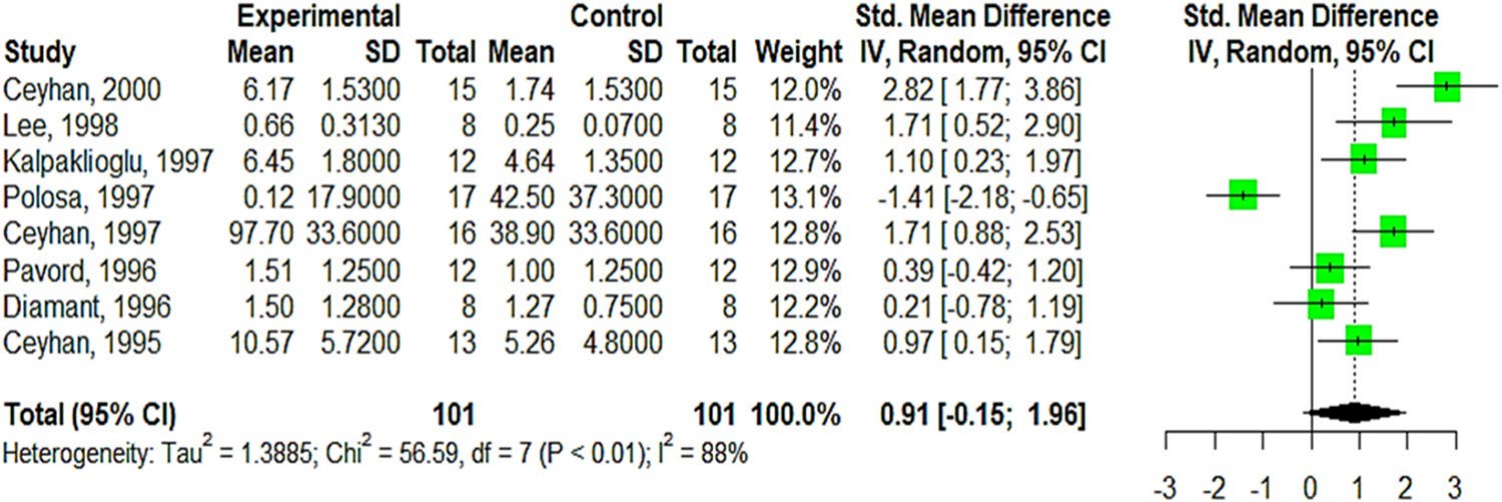
Forest plot illustrates the pooled SMD of PC20 after performing leave-one sensitivity analysis, omit Duong, 2008.

The overall 9 studies with 238 asthmatic participants were included in this meta-analysis. The pooled effect size of PC20 is not statistically significant with a small contribution, SMD = 0.38 (95% CI − 1.2; 1.95, Tau2 = 3.87, I^2^% = 93%), besides a substantial heterogeneity that affects the pooled estimate. The Baujat plot also confirms this as shown in, Duong 2008 is considered an outlier and slightly influences the pooled effect (Supplementary Appendix 2, Fig. S3A,B). Upon omitting Duong 2008, leave-one sensitivity analysis, the SMD is still statistically insignificant with a high contribution to the outcome favoring the use of heparin (SMD 0.91, 95% CI − 0.15;1.96, I^2^% = 85%, Tau2 = 1.39), Fig. 5.

#### Subgroup analysis

Supplementary Appendix 2, subgroup by heparin type (Fig. S3E), allergen type (Fig. S3F), frequency (Fig. S3G), heparin timing (Fig. S3H), year of publication (Fig. S3K), and study design (Fig. S3L) shows no statistically significant difference. While the subgroup by heparin dose (Fig. S3J) shows that UFH dose 1000 Iu/kg had a highly statistically significant effect on PC20 (SMD 1.27, 95% CI 0.26; 2.29, I^2^ = 74%), and the subgroup by the quality of the study (Fig. S3M) shows that Abstract only studies had a high statistically significant effect on PC20 (SMD 2.06, 95% CI 0.49; 3.63, I^2^ = 34%). Meta-regression could explain 93.19% of between-studies heterogeneity by 2 predictors of quality of the study and heparin type (Good quality, ß =  − 2.76, 95% CI − 4.4; − 1.2, Poor quality, ß =  − 1.25, 95% CI − 2.7; − 0.2, UFH, ß = 4.9, 95% CI 2.7; 7.27).

##### Area under the curve (AUC)

Three studies with 78 asthmatic participants reported AUC, Duong 2008, Tahir 1999, and Diamant 1996. Although the individual SMD was statistically significant and favored using heparin (SMD 0.92, 0, 1.92; 95% CI 0.23–1.61, 0, 0.68–3.16) respectively, the pooled estimate is not statistically significant, either by omitting Tahir 1999 no difference was found, and also seems to favor the heparin side (SMD 1.28; 95% CI − 4.85 to 7.41, I^2^% = 45%) (Supplementary Appendix 3, Fig. S6).

#### Effect of inhaled heparin on coagulation factors and bleeding

##### Activated partial thromboplastin time (APTT)

Pavord^61^, Ashoor^40^, Lee^64^, Ahmed^63^, and Duong^42^ reported that inhaled heparin did not affect coagulation and partial thromboplastin time.

##### Plasma anti-factor-X activity

Tahir^54^ reported no effect of inhaled heparin on plasma factor X (Antifactor X was 0.5 IU/ml before and 1 h after nebulized heparin), while Kwasniewski^52^ reported that Nandaparin increased anti-factor activity more than the control group (P < 0.05), and no effect on other coagulation parameters was observed 8–9 days after treatment.

#### Adverse events of inhaled heparin

##### Headache

Two studies reported headache, Ceyhan^62^, said that 61.5% of participants had a headache after inhalation of heparin that self-resolved 1–2 h later, while Duong^42^, only one participant suffer from mild self-limited headache.

##### Bronchospasm

Ceyhan^62^ had one participant withdraw from the trial after heparin inhalation due to suffering from bronchospasm.

##### Serious adverse events

Shute^41^ and Duong^42^ reported that they didn’t find any serious adverse events in their participants.

##### Others

Duong^42^ reported that total lymphocyte count and eosinophil were increased in the placebo arm than in the heparin arm.

##### Adverse events

Polosa^57^, Tranfa^53^, Diamant^58^, Garrigo^59^, Hong^60^ and Motamed^39^ they reported that adverse events not seen at all.

### Publication bias

FEV1% publication bias was assessed in 2 different ways; visual inspection shows an aggregation of most studies on the left side from the diagonal line indicating asymmetry in the funnel plot. Then further confirmation using Eggers’ test: Linear regression test of funnel plot asymmetry (Test result: t = 3.25, df = 8, P-value = 0.017). Rank correlation test using Begg and Mazumdar’s test is not applicable because of the number of studies less than ten. For FEV1 ml, visual inspection shows an aggregation of most studies on the left side from the diagonal line (Supplementary Appendix 2, Figs. S1O, S2P).

## Discussion

Asthma and COPD are among the most widespread obstructive pulmonary diseases on a global scale. Despite the availability of targeted therapies, management of these conditions remains challenging. Therefore, we aimed to assess the effectiveness and safety of inhaled heparin and its derivatives as an alternative or complementary treatment for asthma and COPD. Our analysis demonstrated that inhaled heparin significantly improves pulmonary function, particularly FEV1, and PC20, without increasing the risk of bleeding in adult asthmatic and COPD patients, whether used alone or in combination with other therapies, especially for those with severe or critical conditions. Subgroup analysis revealed that adding UFH as a treatment, at a dose of 1000 IU/Kg, at least 20 min prior to an allergen or exercise provocation test, yielded the highest significant pooled estimate. Most randomized controlled trials did not demonstrate any adverse events and those that did were rare and mild, such as headache and self-limited bronchospasm, with no serious adverse events reported.

Our findings reveal a significant statistical advantage in using inhaled heparin to improve FEV1% in adult asthmatic patients, with a high level of evidence to support this claim. These results are consistent with Yang’s meta-analysis^25^ that examined the use of injectable LMWH in COPD patients and found that it improves FEV1 (MD = 0.19, 95% CI 0.09–0.29, P = 0.0002) but increases the risk of hemorrhage. In addition, heparin may have benefits for various lung diseases. Two meta-analyses by Xiangyue in 2020^65^ and Xinghao in 2020^66^ concluded that low-dose heparin injection and LMWH can improve oxygenation and lung function in patients with acute respiratory distress syndrome (ARDS)/Acute lung injury (ALI), reduce mortality, but may also increase the risk of bleeding.

This meta-analysis extends the systematic review of the inflammatory protective effect of heparin, focusing on the inhaled form for bronchoconstriction-associated diseases^15^. Moreover, the Mongale review, (20 studies, 536 patients), found that earlier studies have indicated that inhalation of UFH treats local inflammation, mucus hypersecretion, and lung injury without systemic anticoagulation or any incidence of pulmonary hemorrhage^67^. The inhalation of heparin suppresses the initial reaction to allergens and exercise-induced asthma, likely by preventing the release of mediators from mast cells.

Our finding matches the conclusion of Fröhlich review^68^ for using oral inhalation is the best way to deliver protein (such as heparin) and peptides for diseases affecting the lungs (e.g. asthma, COVID-19, etc.). In addition to Petris’ review^69^ found that anticoagulation therapy is very important for COPD patients and can reduce their risk of mortality due to some bronchopulmonary changes and pulmonary embolism. Also, it was noted that the antioxidant properties of heparin can contribute to decreased inflammation and safeguard anti-proteins against oxidative inactivation, limit reactive oxygen species (ROS)-induced mucus hypersecretion, and counteract the oxidative stress as a feature of bronchial asthma and COPD, with their wide-ranging effects in the airways and lung parenchyma specifically in COPD patients^13^.

The positive outcomes of heparin found in the literature for the pulmonary route require a focus on the preparation and evaluation of heparin in advanced drug delivery systems, specifically nano/microparticles and liposomes^22^. Moreover, timing is a very important factor in heparin inhalation because the effect of inhaled heparin is not immediate like the short-acting B2 agonist. According to Yildiz et al. review^14^, inhaled heparin is safe and beneficial for treating lung diseases.

### Strengthens

This is the first specific meta-analysis addressing inhaled heparin for asthma and COPD patients. Our comprehensive updated search in 15 databases to synthesize all published evidence regarding this topic, besides all studies included in this meta-analysis were RCT, with high to moderate GRADE evidence. This facilitates the decision of using this important and widely available drug. Sub-group analysis helps in deciding the best dose, time, and formula of heparin because using subtherapeutic doses makes biased negative results.

### Limitation

This meta-analysis majority of included studies are for adult asthmatic patients.

## Conclusion

For COPD patients and children asthmatic patients need more studies for using inhaled heparin in these conditions. New drug delivery formulations need in vivo research to ensure their efficacy in bronchoconstriction diseases. Most of the included studies were cross-over designs with low sample sizes and of high risk of bias, thus we need new research regarding this important route of administration for this drug. The study suggests that inhaled heparin and its derivatives in asthma or COPD exacerbations may be beneficial and could be prescribed in addition to the standard therapy. The right dose, timing, frequency, and duration of heparin therapy should be considered to achieve the best clinical outcomes for those patients.

### Supplementary Information

Supplementary Information.

## Data Availability

Data will be available upon request from the first or corresponding authors.

## Abbreviations

AHRQ: Agency of health care research and quality
BAL: Bronchoalveolar lavage
Cis: Confidence intervals
COPD: Chronic obstructive pulmonary disease
CRP: C-reactive protein
ECP: Esionphilic cationic protein
EIA: Exercise-induced asthma
FEV1: Forced expiratory volume at 1 s
FeNO: Fractional exhaled nitric oxide
FVC: Forced vital capacity
GRADE: Grading of recommendations assessments, development, and, evaluations
ICS: Inhaled corticosteroids
IL: Interleukin
IPF: Interstitial pulmonary fibrosis
IU: International unit
IV: Intravenous
kg: Kilogram
LABA: Long-acting B2 agonist
LAMA: Long-acting antimuscarinic
LMWH: Low molecular weight heparin
MD: Mean difference
MV: Mechanical ventilation
PC20: The amount of allergen percentage increases from the initial to the final concentration which causes a 20% decrease in FEV1
PEFR: Peak expiratory flow rate
PRISMA: Preferred Reporting Items for Systematic Reviews and Meta-Analysis
RCT: Randomized controlled trial
ROB: Risk of bias tool
SABA: Short-acting B2 agonist
SEs: Stand errors
SMD: Standard mean difference
SOC: Standard of care
TH: T-helper cell
UFH: Unfractionated heparin

## References

[1] Yeh GY, Horwitz R (2017). Integrative medicine for respiratory conditions disease asthma COPD respiratory disease integrative medicine. Med. Clin..

[2] Silva GE, Sherrill DL, Guerra S, Barbee RA (2004). Asthma as a risk factor for COPD in a longitudinal study. Chest.

[3] Brasier AR (2014). Heterogeneity in asthma. Adv. Exp. Med. Biol..

[4] Yarmohammadi H, Cunningham-Rundles C (2017). Idiopathic CD4 lymphocytopenia: Pathogenesis, etiologies, clinical presentations and treatment strategies. Ann. Allergy Asthma Immunol..

[5] Loftus PA, Wise SK (2016). Epidemiology of asthma. Curr. Opin. Otolaryngol. Head Neck Surg..

[6] Singh D, Agusti A, Anzueto A, Barnes PJ, Bourbeau J, Celli BR (2019). Global strategy for the diagnosis, management, and prevention of chronic obstructive lung disease: The GOLD science committee report 2019. Eur. Respir. J..

[7] Wenzel SE (2012). Asthma phenotypes: The evolution from clinical to molecular approaches. Nat. Med..

[8] Barnes PJ (2016). Inflammatory mechanisms in patients with chronic obstructive pulmonary disease. J. Allergy Clin. Immunol..

[9] *Pocket Guide for Asthma Management and Prevention—Global Initiative for Asthma—GINA*. https://ginasthma.org/pocket-guide-for-asthma-management-and-prevention/ (2022).

[10] *2022 GOLD Reports—Global Initiative for Chronic Obstructive Lung Disease—GOLD*. https://goldcopd.org/2022-gold-reports-2/ (2022).

[11] Pedersen S (2006). Clinical safety of inhaled corticosteroids for asthma in children: An update of long-term trials. Drug Saf..

[12] Barnes PJ (2013). Corticosteroid resistance in patients with asthma and chronic obstructive pulmonary disease. J. Allergy Clin. Immunol..

[13] Patel B, Rashid J, Gupta N, Ahsan F (2017). Low-molecular-weight heparin-coated and montelukast-filled inhalable particles: A dual-drug delivery system for combination therapy in asthma. J. Pharm. Sci..

[14] Yildiz-Pekoz A, Ozsoy Y (2017). Inhaled heparin: Therapeutic efficacy and recent formulations. J. Aerosol. Med. Pulm. Drug Deliv..

[15] Mousavi, S. & Moradi, M. *Anti-inflammatory Effects of Heparin and Its Derivatives: A Systematic Review‏. hindawi.com*. https://www.hindawi.com/journals/aps/2015/507151/abs/ (2015). 10.1155/2015/507151 PMC4443644 26064103

[16] Virk H, Arthur G, Bradding P (2016). Mast cells and their activation in lung disease. Transl. Res..

[17] Yang IV, Lozupone CA, Schwartz DA (2017). The environment, epigenome, and asthma. J. Allergy Clin. Immunol..

[18] Caramori G, Casolari P, Barczyk A, Durham AL, Di Stefano A, Adcock I (2016). COPD immunopathology. Semin. Immunopathol..

[19] Lanzetti M, Da Costa CA, Nesi RT, Barroso MV, Martins V, Victoni T (2012). Oxidative stress and nitrosative stress are involved in different stages of proteolytic pulmonary emphysema. Free Radic. Biol. Med..

[20] Shute JK (2023). Heparin, low molecular weight heparin, and non-anticoagulant derivatives for the treatment of inflammatory lung disease. Pharm..

[21] Lever R, Smailbegovic A, Page CP (2010). Locally available heparin modulates inflammatory cell recruitment in a manner independent of anticoagulant activity. Eur. J. Pharmacol..

[22] Shute JKJK, Puxeddu E, Calzetta L (2018). Therapeutic use of heparin and derivatives beyond anticoagulation in patients with bronchial asthma or COPD. Curr. Opin. Pharmacol..

[23] Yildiz-Pekoz A. *YO Aerosol Medicine and Pulmonary Drug. Inhaled Heparin: Therapeutic Efficacy and Recent Formulations‏. liebertpub.com*‏. 10.1089/jamp.2015.1273 (2021). 10.1089/jamp.2015.1273 28418758

[24] Monagle K, Merten E, Hepponstall M, Monagle P, Newall F (2011). Inhalational use of antithrombotic agents: Evidence for use. J. Thromb. Haemost..

[25] Yang M, Xu Y, Chen H, Xu Z, Luo F (2020). Benefits and risks of low molecular weight heparin in patients with acute exacerbation of chronic obstructive pulmonary disease: A meta-analysis of randomized controlled trials. Inflammopharmacology.

[26] Liberati. *PRISMA Checklist*. http://prisma-statement.org/ (2009).

[27] Julian Higgins, J. T. *Cochrane Handbook for Systematic Reviews of Interventions|Cochrane Training. 6.3*. https://training.cochrane.org/handbook/current (2022).

[28] *Risk of Bias Tools—Current Version of RoB 2*. https://www.riskofbias.info/welcome/rob-2-0-tool/current-version-of-rob-2 (2021).

[29] Viswanathan M, Ansari MT, Berkman ND, Chang S, Hartling L, McPheeters LM, Treadwell JR (2012). Methods guide for comparative effectiveness reviews assessing the risk of bias of individual studies in systematic reviews of health care interventions. Agency Healthc. Res. Qual. Methods Guid. Comp. Eff. Rev..

[30] Andrade C (2020). Mean difference, standardized mean difference (SMD), and their use in meta-analysis: As simple as it gets. J. Clin. Psychiatry.

[31] *9.3 Study Designs and Identifying the Unit of Analysis*. https://handbook-5-1.cochrane.org/chapter_9/9_3_study_designs_and_identifying_the_unit_of_analysis.htm (2022).

[32] *16 Special Topics in Statistics*. https://handbook-5-1.cochrane.org/chapter_16/16_special_topics_in_statistics.htm (2022).

[33] Review Manager (RevMan). https://community.cochrane.org/help/tools-and-software/revman-5/revman-5-download/installation (The Nordic Cochrane Centre, The Cochrane Collaboration, 2014).

[34] Harrer M, Cuijpers P, Furukawa TA, Ebert DD, Harrer M, Cuijpers P, Furukawa TA, Ebert DD (2021). Chapter 5 between-study heterogeneity. Doing Meta-Analysis in R: A Hands-On Guide.

[35] Viechtbauer W, Cheung MW-L (2010). Outlier and influence diagnostics for meta-analysis. Res. Synth. Methods.

[36] Baujat B, Mahé C, Pignon JP, Hill C (2002). A graphical method for exploring heterogeneity in meta-analyses: Application to a meta-analysis of 65 trials. Stat. Med..

[37] Schünemann, H. *Introduction to GRADE Handbook*. www.gradeworkinggroup.org (2013).

[38] GRADEpro Guideline Development Tool. *GRADEpro GDT (Software). McMaster University and Evidence Prime*. https://www.gradepro.org/ (2022).

[39] Motamed H, Verki MM, Nematollahi AV, Hesam S (2021). Evaluation of efficacy of nebulized low molecular weight heparin as an adjunctive extra treatment for acute mild-moderate asthma attack; a randomized clinical trial study. Pulm. Pharmacol. Ther..

[40] Ashoor TM, Hasseb AM, Esmat IM (2020). Nebulized heparin and salbutamol versus salbutamol alone in acute exacerbations of chronic obstructive pulmonary disease requiring mechanical ventilation: A double-blind randomized controlled trial. Korean J. Anesthesiol..

[41] Shute JK, Calzetta L, Cardaci V, di Toro S, Page CP, Cazzola M (2018). Inhaled nebulised unfractionated heparin improves lung function in moderate to very severe COPD: A pilot study. Pulm. Pharmacol. Ther..

[42] Duong M, Cockcroft D, Boulet LP, Ahmed T, Iverson H, Atkinson DC (2008). The effect of IVX-0142, a heparin-derived hypersulfated disaccharide, on the allergic airway responses in asthma. Allergy Eur. J. Allergy Clin. Immunol..

[43] Abd-Elaty NM, Elprince M (2007). Abstracts of the XX World Allergy Congress TM 2007 ORAL ABSTRACT SESSIONS. World Allergy Organ. J..

[44] Liu X, Liu XJL (2006). Clinical study of heparin spray inhalation in treatment of bronchial asthma. Chin. Gen. Pract..

[45] Fal AM, Kraus-Filarska M, Miecielica J, Małolepszy J (2003). Mechanisms of action of nebulized low-molecular-weight heparin in patients with bronchial asthma. Pol. Merkur. Lek..

[46] Stelmach I, Jerzynska J, Stelmach W, Majak P, Brzozowska A, Gorski P (2003). The effect of inhaled heparin on airway responsiveness to histamine and leukotriene D4. Allergy Asthma Proc..

[47] Passowicz-Muszyńska E, Jankowska R, Krasnowska M (2002). The effect of inhaled low molecular weight heparin on cell composition in bronchoalveolar lavage fluid and serum levels of soluble receptor of IL-2 in asthmatics. Pol. Merkur. Lek..

[48] Stelmach I, Jerzyńska J, Bobrowska M, Brzozowska A, Majak P, Kuna P (2002). The effect of inhaled heparin on postleukotriene bronchoconstriction in children with asthma. Pol. Merkur. Lek..

[49] Stelmach I, Jerzyńska J, Bobrowska M, Brzozowska A, Majak P, Kuna P (2001). The effect of inhaled heparin on airway responsiveness to metacholine in asthmatic children|Wplyw wziewnej heparyny na pometacholinowy skurcz oskrzeli u dzieci z astma̧ oskrzelowa̧. Pol. Arch. Med. Wewn..

[50] Tutluoglu B, Gürbüz N, Atis S, Abanozlu S, Ibis R, Kanik A (2001). Effects of heparin on hypertonic potassium chloride-induced bronchoconstriction. Ann. Pharmacother..

[51] Ceyhan BBB, Celikel T (2000). Effect of inhaled low molecular weight heparin on methacholine-induced bronchoconstriction. Int. J. Clin. Pharmacol. Ther..

[52] Kwasniewski A, Krasnowska M, Korbuszewska-Gontarz B (2000). Effect of inhalation of low molecular weight heparin on some blood clotting parameters in patients with bronchial asthma. Allergy Asthma Immunol..

[53] Tranfa CMEME, Vatrella A, Parrella R, Pelaia G, Bariffi F, Marsico SAA (2001). Effect of inhaled heparin on water-induced bronchoconstriction in allergic asthmatics. Eur. J. Clin. Pharmacol..

[54] Ahmed T, Gonzalez BJ, Danta I (1999). Prevention of exercise-induced bronchoconstriction by inhaled low-molecular-weight. Heparin.

[55] Ceyhan BBB, Çelikel T (1997). Effect of inhaled heparin on adenosine-induced bronchial hyperreactivity. Int. J. Clin. Pharmacol. Ther..

[56] Kalpaklioglu AF, Demirel YS, Saryal S, Misirligil Z, Kalpaklioǧlu AF, Demirel YS (1997). Effect of pretreatment with heparin on pulmonary and cutaneous response. J. Asthma.

[57] Polosa R, Magri S, Vancheri C, Armato F, Santonocito G, Mistretta A (1997). Time course of changes in adenosine 5’-monophosphate airway responsiveness with inhaled heparin in allergic asthma. J. Allergy Clin. Immunol..

[58] Diamant Z, Timmers MCMC, Van Der Veen H, Page CPCP, Van Der Meer FJFJ, Sterk PJPJ (1996). Effect of inhaled heparin on allergen-induced early and late asthmatic responses in patients with atopic asthma. Am. J. Respir. Crit. Care Med..

[59] Garrigo J, Danta I, Ahmed T (1996). Time course of the protective effect of inhaled heparin on exercise-induced asthma. Am. J. Respir. Crit. Care Med..

[60] Hong Y, Wang X (1996). Therapeutic observation of heparin inhalation for asthma with 30 children. Acta Acad. Med. Hubei.

[61] Pavord I, Mudassar T, Bennett J, Wilding P, Knox A (1996). The effect of inhaled heparin on bronchial reactivity to sodium metabisulphite and methacholine in patients with asthma. Eur. Respir. J..

[62] Ceyhan B, Celikel T (1995). Effect of inhaled heparin on methacholine-induced bronchial hyperreactivity. Chest.

[63] Ahmed T, JoseGarrigo ID (1993). Preventing bronchoconstriction in exercise-induced asthma with inhaled heparin. N. Engl. J. Med..

[64] Sin Hyung L, Jae Jeong S, Sang Youb L, Jae Youn C, Kwang Ho I, Se Hwa Y (1998). The protective effect of inhaled heparin, cromolyn, budesonide, and furosemide on exercise-induced asthma. Tuberc. Respir. Dis. (Seoul).

[65] Xiangyue J, Ruihua F, Jiangping L, Ya’nan G, Xiubing G, Yehao L (2020). Meta-analysis of the curative effect of low molecular weight heparin on acute respiratory distress syndrome. Zhonghua Wei Zhong Bing Ji Jiu Yi Xue.

[66] Xinghao Z, Feng S, Xiang L, Guixia Y, Yumei C, Tianhui H (2020). Meta-analysis of the impact of continuous anticoagulation therapy with low-dose heparin or low molecular weight heparin on the prognosis of patients with acute respiratory distress syndrome. Zhonghua Wei Zhong Bing Ji Jiu Yi Xue.

[67] Monagle K, Ryan A, Hepponstall M, Mertyn E, Monagle P, Ignjatovic V (2015). Inhalational use of antithrombotics in humans: Review of the literature. Thromb. Res..

[68] Fröhlich E, Salar-Behzadi S (2021). Oral inhalation for delivery of proteins and peptides to the lungs. Eur. J. Pharm. Biopharm..

[69] Petris OR, Cojocaru E, Fildan AP, Cojocaru C (2021). COPD and anticoagulation therapy: Time for a new approach?. Int. J. COPD.

