# Supplementary material for: Efficacy and safety of inhaled heparin in asthmatic and chronic obstructive pulmonary disease patients: a systematic review and a meta-analysis
Source: Sci Rep. 2023 Aug 16;13:13326. doi: 10.1038/s41598-023-40489-8 (PMC10432425; doi:10.1038/s41598-023-40489-8)
Supplement: Supplementary file 1 — Supplementary Information. [file 41598_2023_40489_MOESM1_ESM.pdf]

# Supplementary material

| | |
|---|---|
| **Appendix 1** | **Detailed search strategy** |
| **Appendix 2** | **Forest plot before sensitivity analysis, Baujat plot, sensitivity analysis, subgroup analysis, and publication bias of FEV1%, FEV1 ml and PC20 outcomes.** |
| **Appendix 3** | **GRADE table (before and after sensitivity) of FEV1% FEV1 ml and PC20** |
| **Appendix 4** | **Forest plot of other outcomes (FVC, PEFR, AUC)** |
| **Appendix 5** | **Narrative synthesis of included studies.** |
| **Appendix 6** | **PRISMA Checklist** |

# Appendix 1

## Literature search strategy (main search and updated)

**Table S1: showing detailed database search strategy and used keywords.**

| | Database | Keywords | |
|---|---|---|---|
| 1 | PubMed | ("chronic obstructive pulmonary disease"[All Fields] OR ("pulmonary disease, chronic obstructive"[MeSH Terms] OR ("pulmonary"[All Fields] AND "disease"[All Fields] AND "chronic"[All Fields] AND "obstructive"[All Fields]) OR "chronic obstructive pulmonary disease"[All Fields] OR "copd"[All Fields]) OR ("asthma"[MeSH Terms] OR "asthma"[All Fields] OR "asthmas"[All Fields] OR "asthma s"["ll Fields]) OR "status asthmaticus"[A"l fields] OR ("bronchoconstricted"[All Fields] OR "bronchoconstricting"[All Fields] OR "bronchoconstriction"[MeSH Terms] OR "bronchoconstriction"[All Fields] OR "bronchoconstrictions"[All Fields] OR "bronchoconstrictive"[All Fields]) OR (("airway"[All Fields] OR "airway s"[All Fields] OR "airways"[All Fields]) AND ("2sthma2rin2t"[All Fields] OR "inflamations"[All Fields])) OR "airway hyperresponsivness"[All Fields] OR ("bronchial spasm"[MeSH Terms] OR ("bronchial"[All Fields] AND "spasm"[All Fields]) OR "bronchial spasm"[All Fields] OR "bronchospasm"[All Fields] OR "bronchospasms"[All Fields]) OR ("aecopd"[All Fields] OR "aecopds"[All Fields]) OR "chronic airflow obstruction"[All Fields] OR "Chronic Obstructive Lung Disease"[All Fields] OR "Chronic Obstructive Airway Disease"[All Fields] OR "Pulmonary Emphysema"[All Fields] OR "Asthmatic Crisis"[All Fields] OR ("status | 2147 |

| | | | |
|---|---|---|---|
| | | **asthmaticus"[MeSH Terms] OR ("status"[All Fields] AND "asthmaticus"[All Fields]) OR "status asthmaticus"[All Fields] OR ("asthmatic"[All Fields] AND "shock"[All Fields]) OR "asthmatic shock"[All Fields]) OR "occupational asthma*"[All Fields] OR ("asthma, exercise induced"[MeSH Terms] OR ("asthma"[All Fields] AND "exercise induced"[All Fields]) OR "exercise-induced asthma"[All Fields] OR ("asthma"[All Fields] AND "exercise"[All Fields] AND "induced"[All Fields]) OR "asthma exercise induced"[All Fields]) OR "bronchial spasm*"[All Fields] OR ("exertion"[All Fields] OR "exertional"[All Fields] OR "exertions"[All Fields]) OR "nsaid induced asthma*"[All Fields] OR "Asthma-COPD Overlap Syndrome"[All Fields] OR "chronic bronchitis"[All Fields] OR ("airway obstruction"[MeSH Terms] OR ("airway"[All Fields] AND "obstruction"[All Fields]) OR "airway obstruction"[All Fields] OR "choked"[All Fields] OR "choking"[All Fields])) AND ("heparin"[MeSH Terms] OR "heparin"[All Fields] OR "heparine"[All Fields] OR "heparins"[All Fields] OR "heparin s"[All Fields] OR "heparinate"[All Fields] OR "heparinated"[All Fields] OR "heparines"[All Fields] OR "heparinic"[All Fields] OR "3sthma3rin3ted"[All Fields] OR "heparinised"[All Fields] OR "heparinization"[All Fields] OR "heparinize"[All Fields] OR "heparinized"[All Fields] OR "heparinizing"[All Fields] OR ("heparin, low molecular weight"[MeSH Terms] OR ("heparin"[All Fields] AND "low molecular weight"[All Fields]) OR "low-molecular-weight heparin"[All Fields] OR "lmwh"[All Fields]) OR "low molecular weight heparin"[All Fields] OR "inhaled heparin"[All Fields] OR "nebulized heparin"[All Fields] OR (("heparin"[MeSH Terms] OR "heparin"[All Fields] OR "heparine"[All Fields]** | |

| | | | |
|---|---|---|---|
| | | **OR “heparins”[All Fields] OR “heparin s”[All Fields] OR “heparinate”[All Fields] OR “heparinated”[All Fields] OR “heparines”[All Fields] OR “heparinic”[All Fields] OR “4sthma4rin4ted”[All Fields] OR “heparinised”[All Fields] OR “heparinization”[All Fields] OR “heparinize”[All Fields] OR “heparinized”[All Fields] OR “heparinizing”[All Fields]) AND “dervatives”[All Fields]) OR “heparan sulfate”[All Fields] OR “heparin-like molecules”[All Fields] OR (“anticoagulants”[Pharmacological Action] OR “anticoagulants”[MeSH Terms] OR “anticoagulants”[All Fields] OR “anticoagulant”[All Fields] OR “4sthma4rin4ted”[All Fields] OR “anticoagulated”[All Fields] OR “anticoagulating”[All Fields] OR “anticoagulation”[All Fields] OR “anticoagulations”[All Fields] OR “anticoagulative”[All Fields]) OR “unfractionated heparin sodium”[All Fields] OR “inhaled anticoagulation”[All Fields] OR “antithrombotic therapy”[All Fields] OR ((“anticoagulants”[Pharmacological Action] OR “anticoagulants”[MeSH Terms] OR “anticoagulants”[All Fields] OR “anticoagulant”[All Fields] OR “4sthma4rin4ted”[All Fields] OR “anticoagulated”[All Fields] OR “anticoagulating”[All Fields] OR “anticoagulation”[All Fields] OR “anticoagulations”[All Fields] OR “anticoagulative”[All Fields]) AND (“oligosaccharides”[MeSH Terms] OR “oligosaccharides”[All Fields] OR “oligosaccharide”[All Fields] OR “oligosaccharidic”[All Fields])) OR “enoxaparin*”[All Fields] OR “dalteparin*”[All Fields] OR “nadroparin*”[All Fields] OR (“heparin”[MeSH Terms] OR “heparin”[All Fields] OR “liquaemin”[All Fields]) OR “fragmin*”[All Fields] OR (“enoxaparin”[MeSH Terms] OR** | |

| | | | |
|---|---|---|---|
| | | **“enoxaparin”[All Fields] OR “enoxaparine”[All Fields] OR “lovenox”[All Fields] OR “enoxaparin s”[All Fields] OR “enoxaparins”[All Fields]) OR “clexan*”[All Fields] OR “fraxiparin*”[All Fields] OR (“tinzaparin”[MeSH Terms] OR “tinzaparin”[All Fields] OR “5sthma5”[All Fields]) OR (“heparan sulfate proteoglycans”[MeSH Terms] OR (“heparan”[All Fields] AND “sulfate”[All Fields] AND “proteoglycans”[All Fields]) OR “heparan sulfate proteoglycans”[All Fields] OR “hspg”[All Fields]) OR “Proteoheparan Sulfate”[All Fields])** | |
| **2** | **Scopus** | **(COPD OR asthma OR ‘chronic lung obstruction’ OR ‘chronic obstructive pulmonary disease’ OR bronchoconstriction OR emphysema OR ‘lung hyperreactivity’ OR ‘airway inflammation’ OR bronchospasm OR exertion) AND (anticoagulant OR heparin* OR LMWH OR ‘low molecular weight heparin’ OR UFH OR enoxaparin* OR Clexane OR Innohep OR fraxiparin* OR 5sthma5rin* OR dalteparin*) AND (inhaled OR nebulized)** | **239** |
| **3** | **Cochrane central** | **#1 asthma OR COPD OR “chronic obstructive pulmonary disease” OR bronchoconstriction OR emphysema OR airway obstruction OR bronchospasm OR AECOPD**<br>**#2 heparin OR unfractionated heparin OR UFH OR LMWH OR anticoagulants**<br>**#3 #1 AND #2 in Trials** | **126** |
| **4** | **Embase** | **‘chronic airflow obstruction’ OR ‘chronic airway obstruction’ OR ‘chronic obstructive bronchitis’** | **2732** |

| | | | |
|---|---|---|---|
| | | **OR ‘chronic obstructive bronchopulmonary disease’ OR ‘chronic obstructive lung disease’ OR ‘chronic obstructive lung disorder’ OR ‘chronic obstructive pulmonary disease’ OR ‘chronic obstructive pulmonary disorder’ OR ‘chronic obstructive respiratory disease’ OR ‘copd’ OR ‘lung chronic obstructive disease’ OR ‘lung disease, chronic obstructive’ OR ‘lung diseases, obstructive’ OR ‘obstructive lung disease’ OR ‘obstructive lung disease, chronic’ OR ‘obstructive pulmonary disease’ OR ‘obstructive respiratory disease’ OR ‘obstructive respiratory tract disease’ OR ‘pulmonary disease, chronic obstructive’ OR ‘pulmonary disorder, chronic obstructive’ OR ‘asthma’/exp OR ‘asthma’ OR ‘asthma bronchiale’ OR ‘asthma pulmonale’ OR ‘asthma, bronchial’ OR ‘asthmatic’ OR ‘asthmatic subject’ OR ‘bronchial asthma’ OR ‘bronchus asthma’ OR ‘childhood asthma’ OR ‘chronic asthma’ OR ‘lung allergy’ OR ‘obstructive airway disease’/exp OR ‘obstructive airway disease’) NOT** | |

| | | | |
|---|---|---|---|
| | | 'coronavirus disease 2019'/exp AND ('heparin derivative'/exp OR 'low molecular weight heparin'/exp)) AND ('clinical article'/de OR 'clinical study'/de OR 'clinical trial'/de OR 'clinical trial topic'/de OR 'cohort analysis'/de OR 'comparative study'/de OR 'control group'/de OR 'controlled clinical trial'/de OR 'controlled study'/de OR 'crossover procedure'/de OR 'double blind procedure'/de OR 'drug dose comparison'/de OR 'human'/de OR 'intermethod comparison'/de OR 'major clinical study'/de OR 'meta analysis'/de OR 'meta analysis topic'/de OR 'methodology'/de OR 'multicenter study'/de OR 'normal human'/de OR 'observational study'/de OR 'phase 2 clinical trial topic'/de OR 'prospective study'/de OR 'randomized controlled trial'/de OR 'randomized controlled trial topic'/de OR 'retrospective study'/de OR 'single blind procedure'/de OR 'systematic review'/de) | |
| **5** | **EBSCO** | **(COPD OR asthma OR "chronic lung obstruction" OR "chronic obstructive pulmonary disease" OR bronchoconstriction OR emphysema OR "lung hyperreactivity" OR "airway inflammation" OR** | **175** |

| | | | |
|---|---|---|---|
| | | **bronchospasm OR exertion) AND (anticoagulant OR heparin* OR LMWH OR "low molecular weight heparin" OR UFH OR enoxaparin* OR Clexane OR Innohep OR fraxiparin* OR 8sthma8rin* OR dalteparin*)** | |
| **6** | **ProQuest Thesis** | **(COPD OR asthma OR 'chronic obstructive pulmonary disorders' OR bronchoconstriction) AND (anticoagulants OR LMWH OR UFH OR anticoagulants)** | **90** |
| **8** | **Web Of Science** | **(COPD OR asthma OR 'chronic obstructive pulmonary disorders' OR bronchoconstriction) AND (anticoagulants OR LMWH OR UFH OR anticoagulants)** | **222** |
| **9** | **Clinicaltrials.gov** | **(COPD AND heparin)+(COPD AND Anticoagulants)**<br>**( asthma AND heparin OR anticoagulant OR LMWH)=0** | **7** |
| **10** | **WHO clinical trials (ICTRP)** | **(COPD OR asthma OR "chronic lung obstruction" OR "chronic obstructive pulmonary disease" OR bronchoconstriction OR emphysema OR "lung hyperreactivity" OR "airway inflammation" OR bronchospasm OR exertion) AND (anticoagulant OR heparin* OR LMWH OR "low molecular weight heparin" OR UFH OR enoxaparin* OR Clexane OR Innohep OR fraxiparin* OR 8sthma8rin* OR dalteparin*)** | **7** |
| **11** | **Iranian Clinical trials ((IRCT)** | **((((((COPD) OR (Chronic obstructive pulmonary disease)) OR asthma) OR bronchoconstriction) OR emphysema) OR excertion)) AND (((((anticoagulants) OR heparin) OR enoxaparin) OR LMWH) OR UFH)** | **23** |
| **12** | **OVID** | **(COPD OR 8sthma* OR "chronic lung obstruction" OR "chronic obstructive pulmonary disease" OR bronchoconstriction OR emphysema OR 'lung hyperreactivity" Or "airway inflammation" OR bronchospasm OR exertion) AND (anticoagulant* OR** | **52** |

| | | | |
|---|---|---|---|
| | | heparins OR “LMWH OR low molecular weight heparin” OR UFH OR enoxaparin* OR Clexane OR Innohep OR fraxiparin OR tinzaparin OR dalteparin) AND (inhaled OR nebulized) | |
| 13 | medRxiv | (asthma OR COPD OR “chronic obstructive pulmonary disorders” OR bronchoconstriction) AND (anticoagulants OR heparin OR LMWH) | 3 |
| 14 | Google scholar | (asthma OR COPD OR “chronic obstructive pulmonary disorders” OR bronchoconstriction) AND (inhaled anticoagulants OR nebulized heparin OR inhalation LMWH) | 1200 |
| 15 | Researcher Gate | (asthma OR COPD OR “chronic obstructive pulmonary disorders” OR bronchoconstriction) AND (anticoagulants OR  heparin OR LMWH) | 63 |

**Table S2: Updated database search (December 2022):**

| | Database | Keywords | |
|---|---|---|---|
| 1 | PubMed<br>Filter from 2021 | (“chronic obstructive pulmonary disease”[All Fields] OR (“pulmonary disease, chronic obstructive”[MeSH Terms] OR (“pulmonary”[All Fields] AND “disease”[All Fields] AND “chronic”[All Fields] AND “obstructive”[All Fields]) OR “chronic obstructive pulmonary disease”[All Fields] OR “copd”[All Fields]) OR (“asthma”[MeSH Terms] OR “asthma”[All Fields] OR “asthmas”[All Fields] OR “asthma s"[“ll Fields]) OR "status asthmaticus"[A”l fields] OR (“bronchoconstricted”[All Fields] OR “bronchoconstricting”[All Fields] OR | 24 |

| | | | |
|---|---|---|---|
| | | **"bronchoconstriction"[MeSH Terms] OR "bronchoconstriction"[All Fields] OR "bronchoconstrictions"[All Fields] OR "bronchoconstrictive"[All Fields]) OR (("airway"[All Fields] OR "airway s"[All Fields] OR "airways"[All Fields]) AND ("10sthma10rin10t"[All Fields] OR "inflamations"[All Fields])) OR "airway hyperresponsivness"[All Fields] OR ("bronchial spasm"[MeSH Terms] OR ("bronchial"[All Fields] AND "spasm"[All Fields]) OR "bronchial spasm"[All Fields] OR "bronchospasm"[All Fields] OR "bronchospasms"[All Fields]) OR ("aecopd"[All Fields] OR "aecopds"[All Fields]) OR "chronic airflow obstruction"[All Fields] OR "Chronic Obstructive Lung Disease"[All Fields] OR "Chronic Obstructive Airway Disease"[All Fields] OR "Pulmonary Emphysema"[All Fields] OR "Asthmatic Crisis"[All Fields] OR ("status asthmaticus"[MeSH Terms] OR ("status"[All Fields] AND "asthmaticus"[All Fields]) OR "status asthmaticus"[All Fields] OR ("asthmatic"[All Fields] AND "shock"[All Fields]) OR "asthmatic shock"[All Fields]) OR "occupational asthma*"[All Fields] OR ("asthma, exercise induced"[MeSH Terms] OR ("asthma"[All Fields] AND "exercise induced"[All Fields]) OR "exercise-induced asthma"[All Fields] OR ("asthma"[All Fields] AND "exercise"[All Fields] AND "induced"[All Fields]) OR "asthma exercise induced"[All Fields]) OR "bronchial spasm*"[All Fields] OR ("exertion"[All Fields] OR "exertional"[All Fields] OR "exertions"[All Fields]) OR "nsaid induced asthma*"[All Fields] OR "Asthma-COPD Overlap Syndrome"[All Fields] OR "chronic bronchitis"[All Fields] OR ("airway obstruction"[MeSH Terms] OR ("airway"[All Fields] AND "obstruction"[All Fields]) OR** | |

| | | | |
|---|---|---|---|
| | | **"airway obstruction"[All Fields] OR "choked"[All Fields] OR "choking"[All Fields])) AND ("heparin"[MeSH Terms] OR "heparin"[All Fields] OR "heparine"[All Fields] OR "heparins"[All Fields] OR "heparin s"[All Fields] OR "heparinate"[All Fields] OR "heparinated"[All Fields] OR "heparines"[All Fields] OR "heparinic"[All Fields] OR "11sthma11rin11ted"[All Fields] OR "heparinised"[All Fields] OR "heparinization"[All Fields] OR "heparinize"[All Fields] OR "heparinized"[All Fields] OR "heparinizing"[All Fields] OR ("heparin, low molecular weight"[MeSH Terms] OR ("heparin"[All Fields] AND "low molecular weight"[All Fields]) OR "low-molecular-weight heparin"[All Fields] OR "lmwh"[All Fields]) OR "low molecular weight heparin"[All Fields] OR "inhaled heparin"[All Fields] OR "nebulized heparin"[All Fields] OR (("heparin"[MeSH Terms] OR "heparin"[All Fields] OR "heparine"[All Fields] OR "heparins"[All Fields] OR "heparin s"[All Fields] OR "heparinate"[All Fields] OR "heparinated"[All Fields] OR "heparines"[All Fields] OR "heparinic"[All Fields] OR "11sthma11rin11ted"[All Fields] OR "heparinised"[All Fields] OR "heparinization"[All Fields] OR "heparinize"[All Fields] OR "heparinized"[All Fields] OR "heparinizing"[All Fields]) AND "dervatives"[All Fields]) OR "heparan sulfate"[All Fields] OR "heparin-like molecules"[All Fields] OR ("anticoagulants"[Pharmacological Action] OR "anticoagulants"[MeSH Terms] OR "anticoagulants"[All Fields] OR "anticoagulant"[All Fields] OR "11sthma11rin11ted"[All Fields] OR "anticoagulated"[All Fields] OR "anticoagulating"[All Fields] OR "anticoagulation"[All Fields] OR "anticoagulations"[All Fields] OR "anticoagulative"[All Fields]) OR** | |

| | | | |
|---|---|---|---|
| | | **"unfractionated heparin sodium"[All Fields] OR "inhaled anticoagulation"[All Fields] OR "antithrombotic therapy"[All Fields] OR (("anticoagulants"[Pharmacological Action] OR "anticoagulants"[MeSH Terms] OR "anticoagulants"[All Fields] OR "anticoagulant"[All Fields] OR "12sthma12rin12ted"[All Fields] OR "anticoagulated"[All Fields] OR "anticoagulating"[All Fields] OR "anticoagulation"[All Fields] OR "anticoagulations"[All Fields] OR "anticoagulative"[All Fields]) AND ("oligosaccharides"[MeSH Terms] OR "oligosaccharides"[All Fields] OR "oligosaccharide"[All Fields] OR "oligosaccharidic"[All Fields])) OR "enoxaparin*"[All Fields] OR "dalteparin*"[All Fields] OR "nadroparin*"[All Fields] OR ("heparin"[MeSH Terms] OR "heparin"[All Fields] OR "liquaemin"[All Fields]) OR "fragmin*"[All Fields] OR ("enoxaparin"[MeSH Terms] OR "enoxaparin"[All Fields] OR "enoxaparine"[All Fields] OR "lovenox"[All Fields] OR "enoxaparin s"[All Fields] OR "enoxaparins"[All Fields]) OR "clexan*"[All Fields] OR "fraxiparin*"[All Fields] OR ("tinzaparin"[MeSH Terms] OR "tinzaparin"[All Fields] OR "12sthma12"[All Fields]) OR ("heparan sulfate proteoglycans"[MeSH Terms] OR ("heparan"[All Fields] AND "sulfate"[All Fields] AND "proteoglycans"[All Fields]) OR "heparan sulfate proteoglycans"[All Fields] OR "hspg"[All Fields]) OR "Proteoheparan Sulfate"[All Fields])** | |
| **2** | **Scopus** | **(COPD OR asthma OR 'chronic lung obstruction' OR 'chronic obstructive pulmonary disease' OR bronchoconstriction OR emphysema OR 'lung hyperreactivity' OR 'airway inflammation' OR bronchospasm OR exertion) AND (anticoagulant OR** | **7** |

| | | | |
|---|---|---|---|
| | | **heparin* OR LMWH OR 'low molecular weight heparin' OR UFH OR enoxaparin* OR Clexane OR Innohep OR fraxiparin* OR 13sthma13rin* OR dalteparin*) AND (inhaled OR nebulized)** | |
| **3** | **Cochrane central** | **#1 asthma OR COPD OR "chronic obstructive pulmonary disease" OR bronchoconstriction OR emphysema OR airway obstruction OR bronchospasm OR AECOPD**<br>**#2 heparin OR unfractionated heparin OR UFH OR LMWH OR anticoagulants**<br>**#3 #1 AND #2 in Trials** | **19** |
| **4** | **Embase** | **'chronic airflow obstruction' OR 'chronic airway obstruction' OR 'chronic obstructive bronchitis' OR 'chronic obstructive bronchopulmonary disease' OR 'chronic obstructive lung disease' OR 'chronic obstructive lung disorder' OR 'chronic obstructive pulmonary disease' OR 'chronic obstructive pulmonary disorder' OR 'chronic obstructive respiratory disease' OR 'copd' OR 'lung chronic obstructive disease' OR 'lung disease, chronic obstructive' OR 'lung diseases, obstructive' OR 'obstructive lung disease' OR 'obstructive lung disease, chronic' OR 'obstructive pulmonary disease' OR 'obstructive** | **10** |

| | | | |
|---|---|---|---|
| | | **respiratory disease' OR 'obstructive respiratory tract disease' OR 'pulmonary disease, chronic obstructive' OR 'pulmonary disorder, chronic obstructive' OR 'asthma'/exp OR 'asthma' OR 'asthma bronchiale' OR 'asthma pulmonale' OR 'asthma, bronchial' OR 'asthmatic' OR 'asthmatic subject' OR 'bronchial asthma' OR 'bronchus asthma' OR 'childhood asthma' OR 'chronic asthma' OR 'lung allergy' OR 'obstructive airway disease'/exp OR 'obstructive airway disease') NOT 'coronavirus disease 2019'/exp AND ('heparin derivative'/exp OR 'low molecular weight heparin'/exp)) AND ('clinical article'/de OR 'clinical study'/de OR 'clinical trial'/de OR 'clinical trial topic'/de OR 'cohort analysis'/de OR 'comparative study'/de OR 'control group'/de OR 'controlled clinical trial'/de OR 'controlled study'/de OR 'crossover procedure'/de OR 'double blind procedure'/de OR 'drug dose comparison'/de OR 'human'/de OR 'intermethod comparison'/de OR 'major clinical study'/de OR 'meta analysis'/de** | |

| | | | |
|---|---|---|---|
| | | OR ‘meta analysis topic’/de OR ‘methodology’/de OR ‘multicenter study’/de OR ‘normal human’/de OR ‘observational study’/de OR ‘phase 2 clinical trial topic’/de OR ‘prospective study’/de OR ‘randomized controlled trial’/de OR ‘randomized controlled trial topic’/de OR ‘retrospective study’/de OR ‘single blind procedure’/de OR ‘systematic review’/de) | |
| **5** | **EBSCO** | **(COPD OR asthma OR “chronic lung obstruction” OR “chronic obstructive pulmonary disease” OR bronchoconstriction OR emphysema OR “lung hyperreactivity” OR “airway inflammation” OR bronchospasm OR exertion) AND (anticoagulant OR heparin* OR LMWH OR “low molecular weight heparin” OR UFH OR enoxaparin* OR Clexane OR Innohep OR fraxiparin* OR 15sthma15rin* OR dalteparin*)** | **23** |
| **6** | **Web Of Science** | **(COPD OR asthma OR ‘chronic obstructive pulmonary disorders’ OR bronchoconstriction) AND (anticoagulants OR LMWH OR UFH OR anticoagulants)** | **36** |
| **9** | **Clinicaltrials.gov** | **(COPD AND heparin)+(COPD AND Anticoagulants)**<br>**( asthma AND heparin OR anticoagulant OR LMWH)** | **0** |
| **10** | **WHO clinical trials (ICTRP)** | **(COPD OR asthma OR “chronic lung obstruction” OR “chronic obstructive pulmonary disease” OR bronchoconstriction OR emphysema OR “lung hyperreactivity” OR “airway inflammation” OR bronchospasm OR exertion) AND (anticoagulant OR** | **0** |

| | | | |
|---|---|---|---|
| | | **heparin* OR LMWH OR "low molecular weight heparin" OR UFH OR enoxaparin* OR Clexane OR Innohep OR fraxiparin* OR 16sthma16rin* OR dalteparin*)** | |
| **11** | **Iranian Clinical trials ((IRCT)** | **((((((COPD) OR (Chronic obstructive pulmonary disease)) OR asthma) OR bronchoconstriction) OR emphysema) OR excertion)) AND (((((anticoagulants) OR heparin) OR enoxaparin) OR LMWH) OR UFH)** | **0** |
| **12** | **OVID** | **(COPD OR 16sthma* OR "chronic lung obstruction" OR "chronic obstructive pulmonary disease" OR bronchoconstriction OR emphysema OR 'lung hyperreactivity" Or "airway inflammation" OR bronchospasm OR exertion) AND (anticoagulant* OR heparins OR "LMWH OR low molecular weight heparin" OR UFH OR enoxaparin* OR Clexane OR Innohep OR fraxiparin OR tinzaparin OR dalteparin) AND (inhaled OR nebulized)** | **20** |
| **13** | **medRxiv** | **(asthma OR COPD OR "chronic obstructive pulmonary disorders" OR bronchoconstriction) AND (anticoagulants OR heparin OR LMWH)** | **3** |
| **14** | **Google scholar**<br><br>**(filter since 2021)** | **(asthma OR COPD OR "chronic obstructive pulmonary disorders" OR bronchoconstriction) AND (inhaled anticoagulants OR nebulized heparin OR inhalation LMWH)** | **350** |

| | | | |
|---|---|---|---|
| | | **DOI: 10.1055/s-0042-1749395** | |
| **15** | **Researchergate**<br><br>**From 2021** | **(asthma OR COPD OR "chronic obstructive pulmonary disorders" OR bronchoconstriction) AND ( anticoagulants OR heparin OR LMWH)** | **1** |
| | **Total** | | **493** |

# Appendix 2

## Result graphs of FEV1%, FEV1 ml and PC20

# S1. Forced expiratory volume at 1 second % (FEV1%):

| Study | Experimental Mean | Experimental SD | Experimental Total | Control Mean | Control SD | Control Total | Weight | Std. Mean Difference IV, Random, 95% CI |
|---|---|---|---|---|---|---|---|---|
| Doung, 2008 | -26.50 | 2.80 | 18 | -31.00 | 2.80 | 18 | 13.7% | 1.57 [0.81; 2.33] |
| Fal, 2004 | 15.69 | 10.08 | 24 | 0.00 | 14.14 | 24 | 13.9% | 1.26 [0.63; 1.88] |
| Tranfa, 2001 | -10.00 | 3.00 | 8 | -25.00 | 2.00 | 8 | 9.7% | 5.56 [3.15; 7.97] |
| Tutluoglu, 2001 | 0.00 | 4.28 | 20 | -16.40 | 4.28 | 18 | 13.0% | 3.75 [2.66; 4.84] |
| Tahir, 1999 | -19.00 | 5.00 | 13 | -30.00 | 5.00 | 13 | 13.2% | 2.13 [1.14; 3.12] |
| Lee, 1998 | -8.50 | 5.32 | 8 | -19.40 | 12.01 | 8 | 13.1% | 1.11 [0.04; 2.18] |
| Diamant, 1996 | -3.00 | 5.10 | 8 | -10.00 | 5.10 | 8 | 13.0% | 1.30 [0.19; 2.40] |
| Ahmed, 1993 | -9.00 | 5.00 | 12 | -35.00 | 2.00 | 12 | 10.3% | 6.59 [4.41; 8.77] |
| **Total (95% CI)** | | | **111** | | | **109** | **100.0%** | **2.70 [1.00; 4.39]** |

Heterogeneity: $Tau^2 = 3.2415$; $Chi^2 = 45.90$, df = 7 ($P < 0.01$); $I^2 = 85\%$

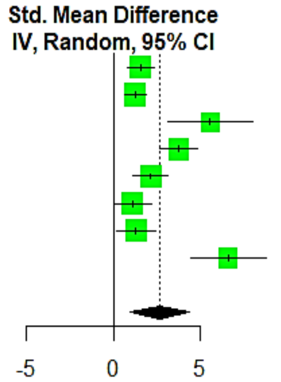


**Figure S1A: Forest plot showing the pooled effect Standard mean difference (SMD) of FEV1%.**

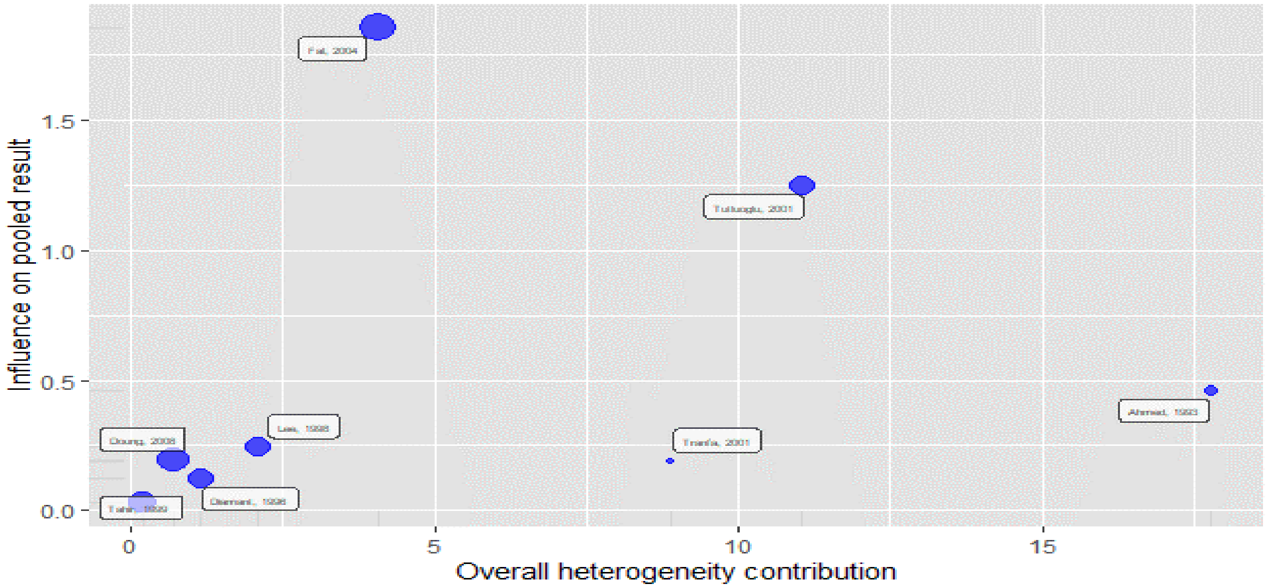


**Figure S1B: Baujat plot for FEV1% checking for influential and outliers, showing contribution of each included study on the pooled effect and the overall heterogeneity.**

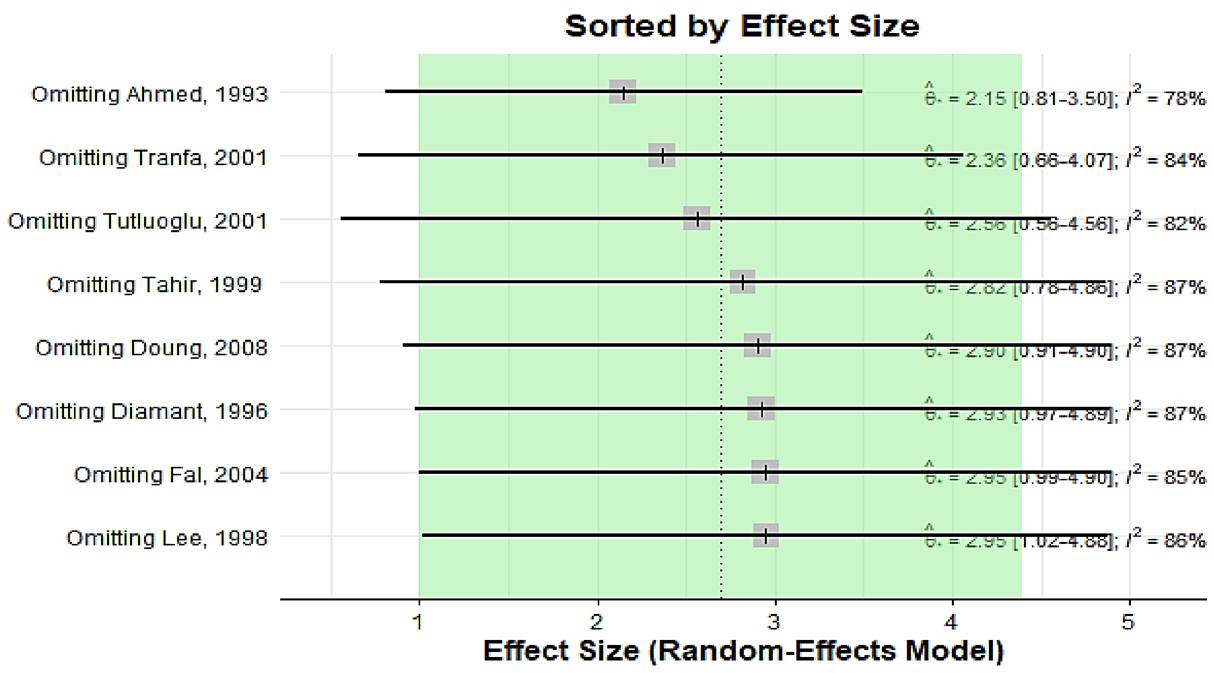


**Figure S1C: Diagram showing the net resulted pooled effect (SMD, CI) and heterogeneity I² of FEV1% after conducting leave one sensitivity analysis per study.**

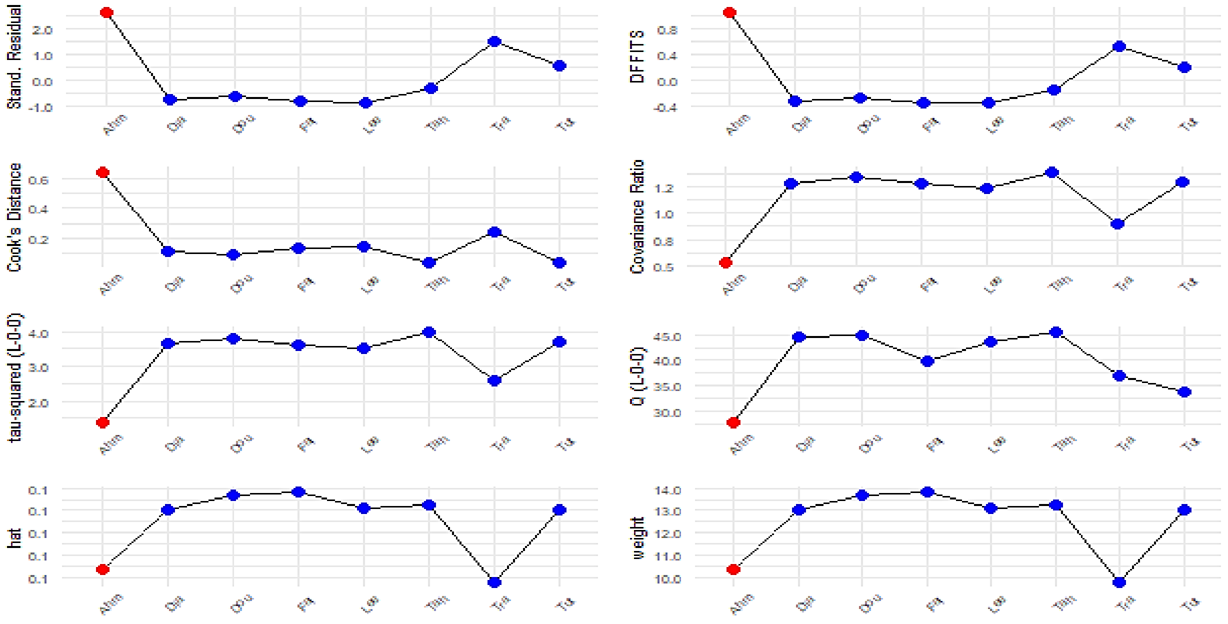


**Figure S1D: Diagram shows different calculated influence diagnostics for SMD of FEV1%.**

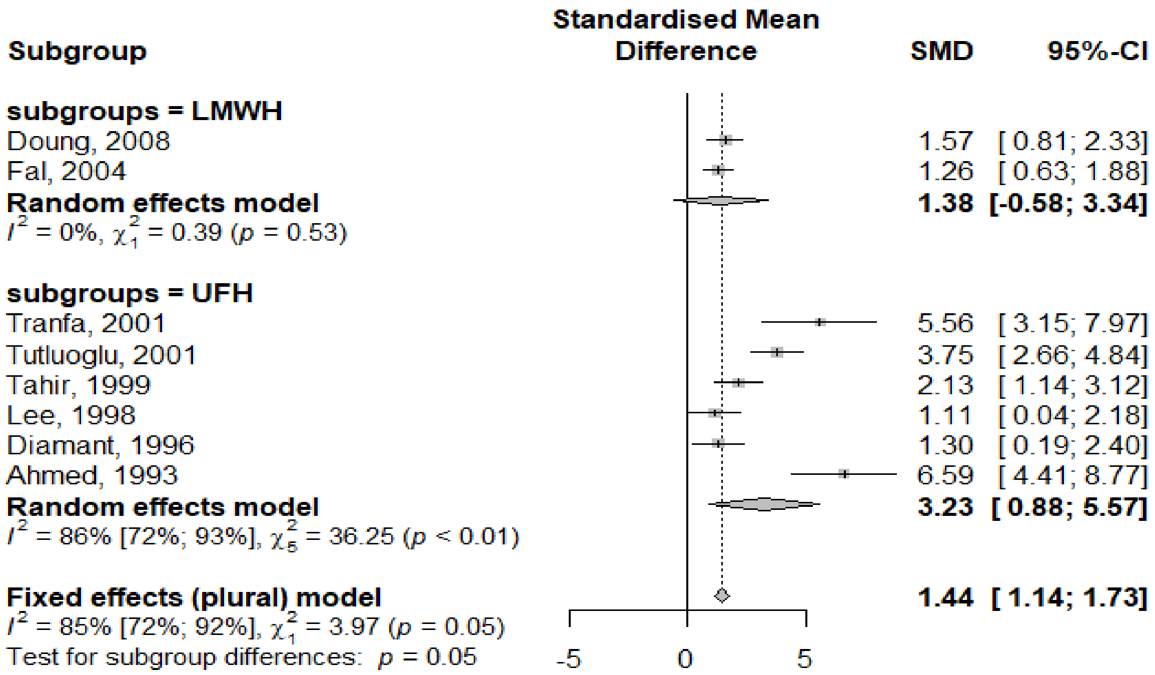


**Figure S1E: Subgroup analysis of SMD of FEV1% by heparin type.**

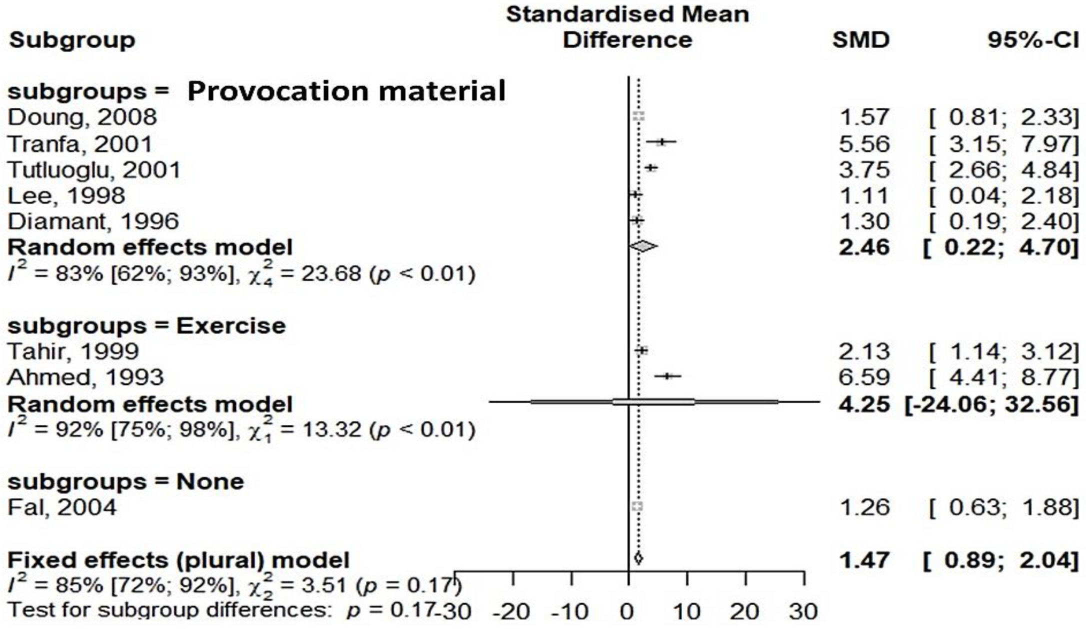


**Provocation material**: material used at provocation test either Methacholine, Histamine, Hypertonic potassium chloride, or Ultrasonically nebulized distilled water.

**Figure S1F: Subgroup analysis of SMD of FEV1% by Provocation test.**

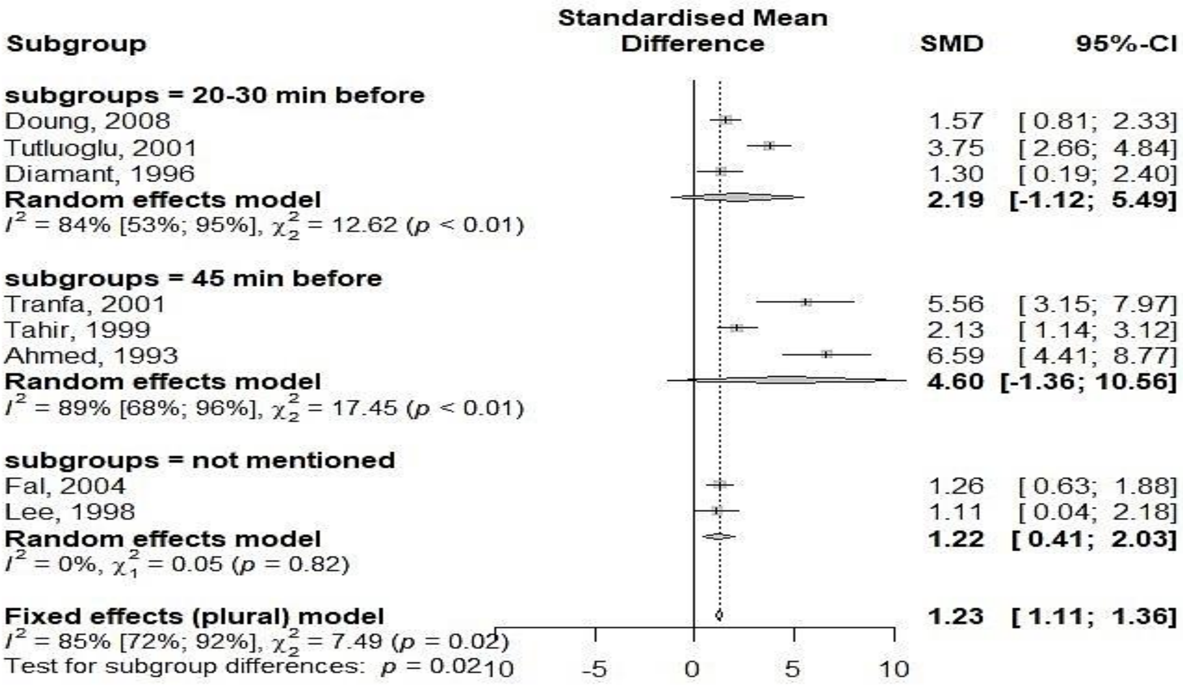


**Figure S1G: Subgroup analysis of SMD of FEV1% by heparin timing.**

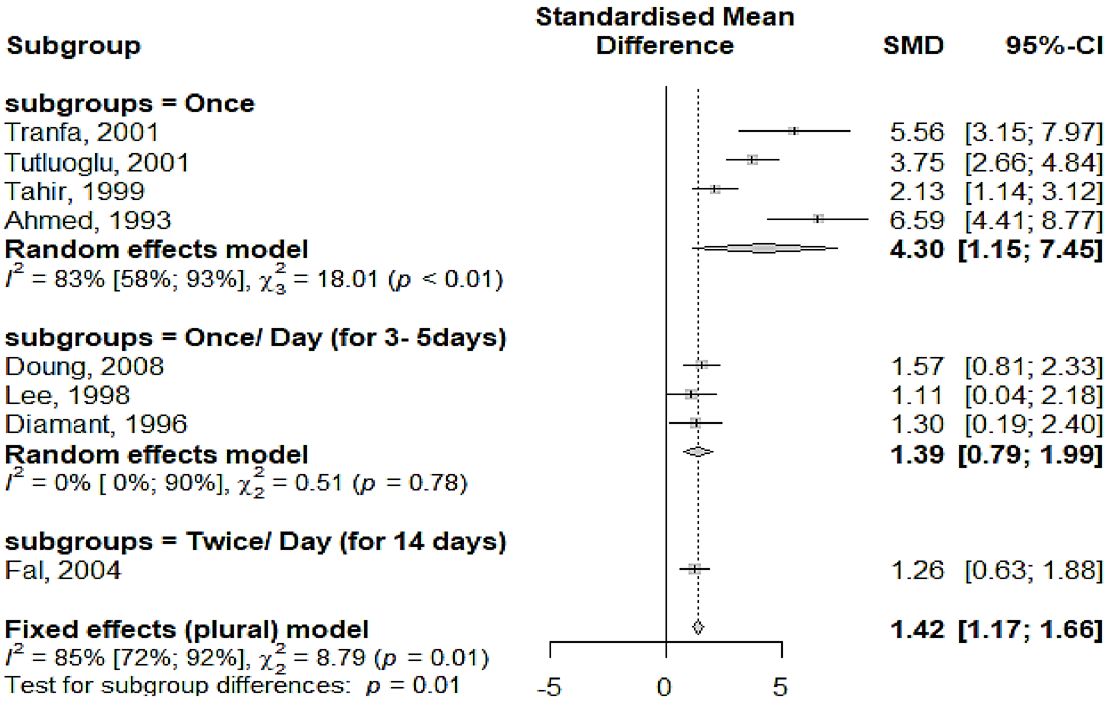


**Figure S1H: Subgroup analysis of SMD of FEV1% by frequency of administration.**

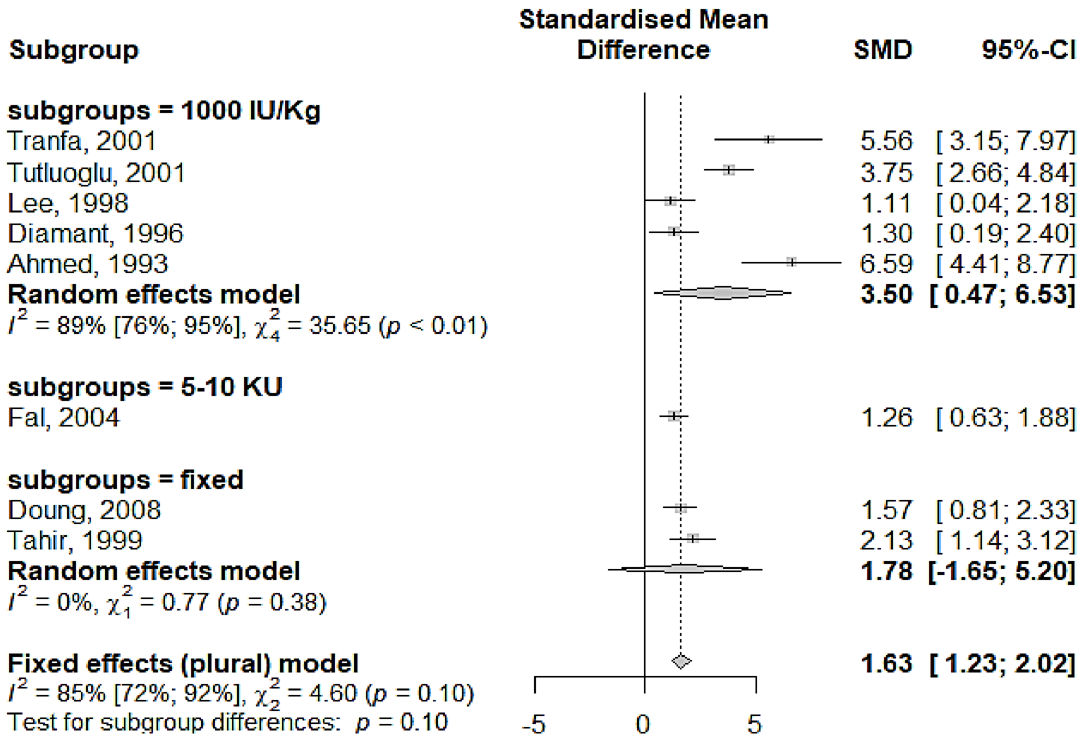


**Figure S1J: Subgroup analysis of SMD of FEV1% by heparin dose.**

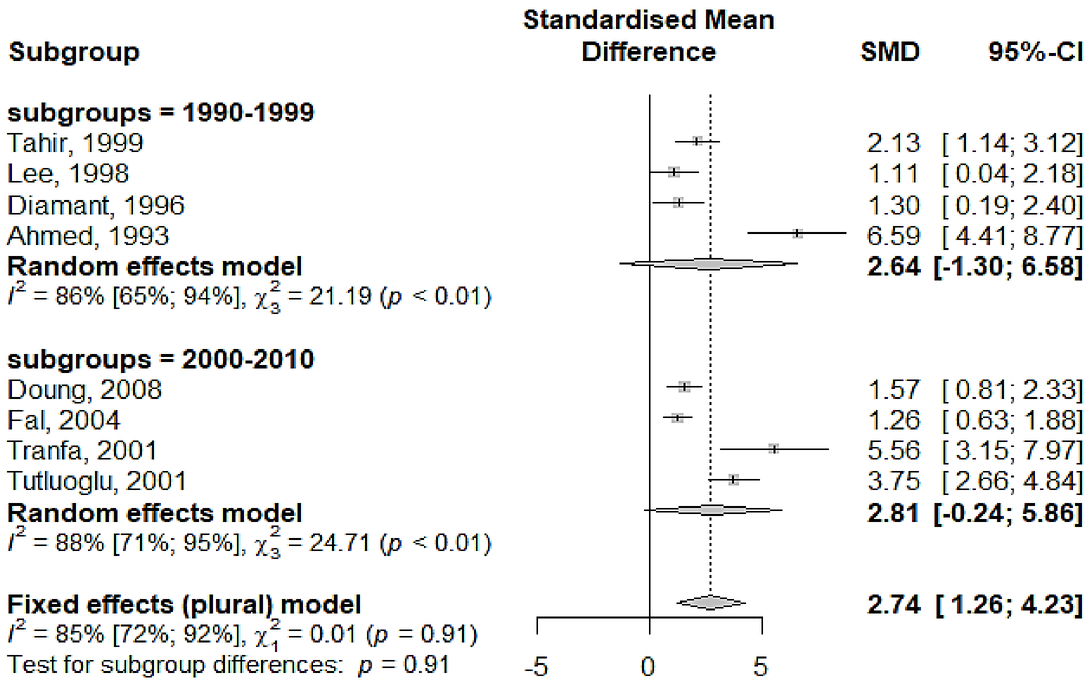


**Figure S1K: Subgroup analysis of SMD of FEV1% by publication year category.**

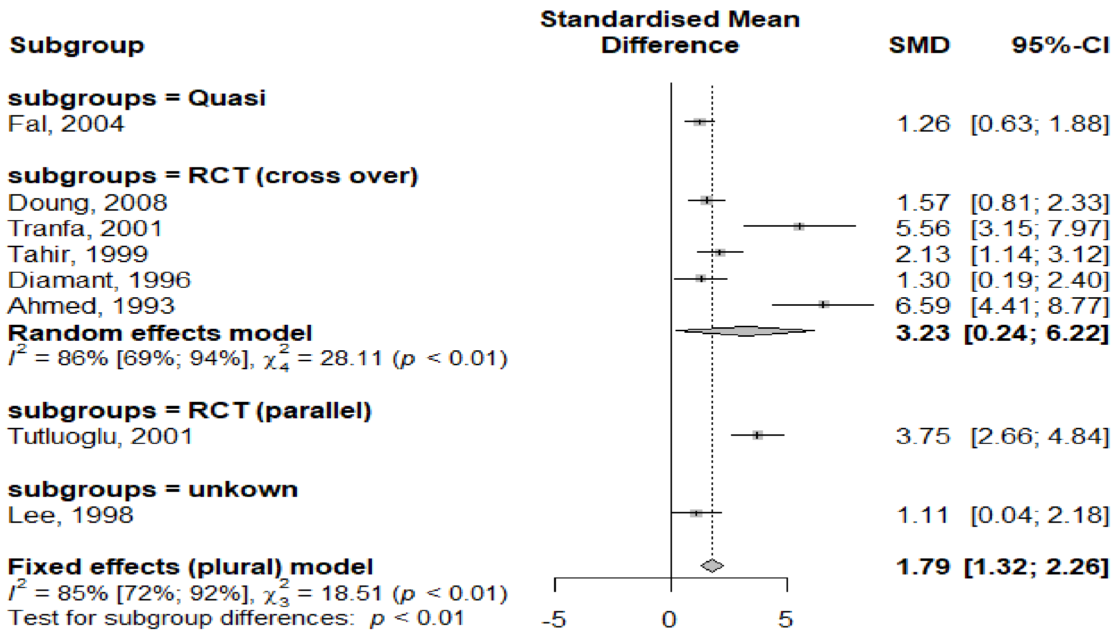


**Figure S1L: Subgroup analysis of SMD of FEV1% by study design.**

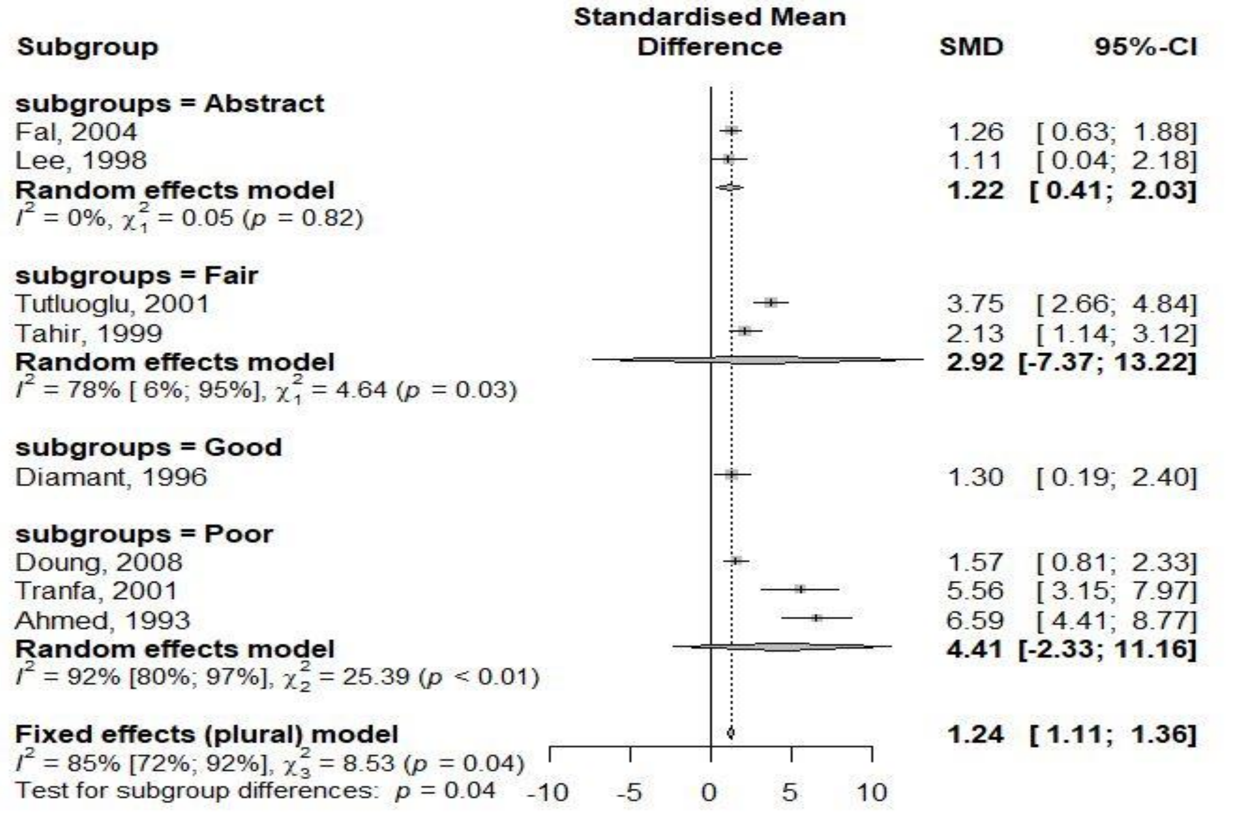


**Figure S1M: Subgroup analysis of SMD of FEV1% by quality of study**

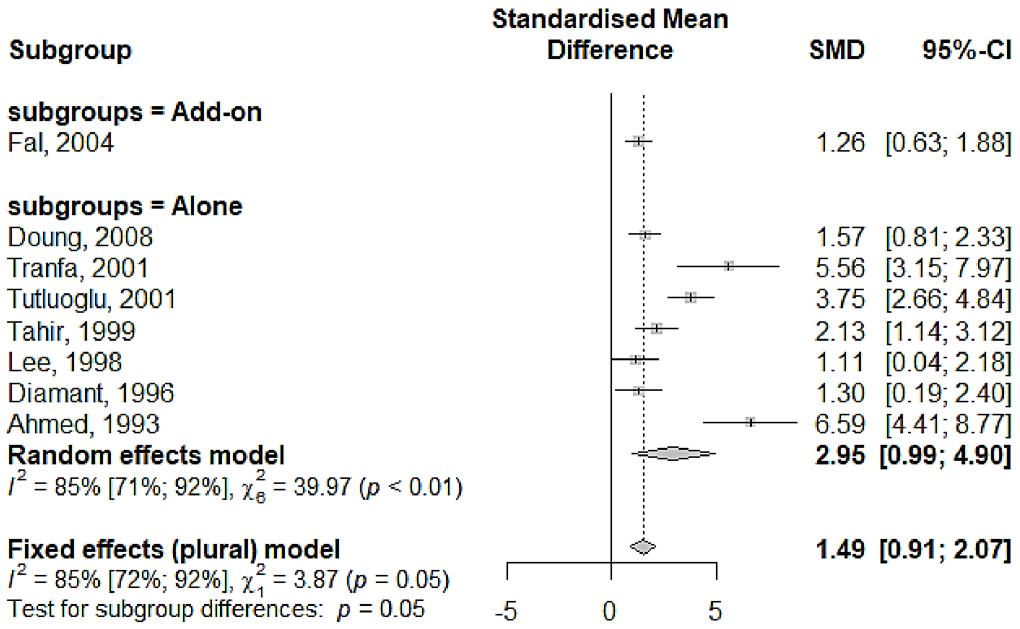


**Figure S1N: Subgroup analysis of SMD of FEV1% by heparin treatment type either add on or alone.**

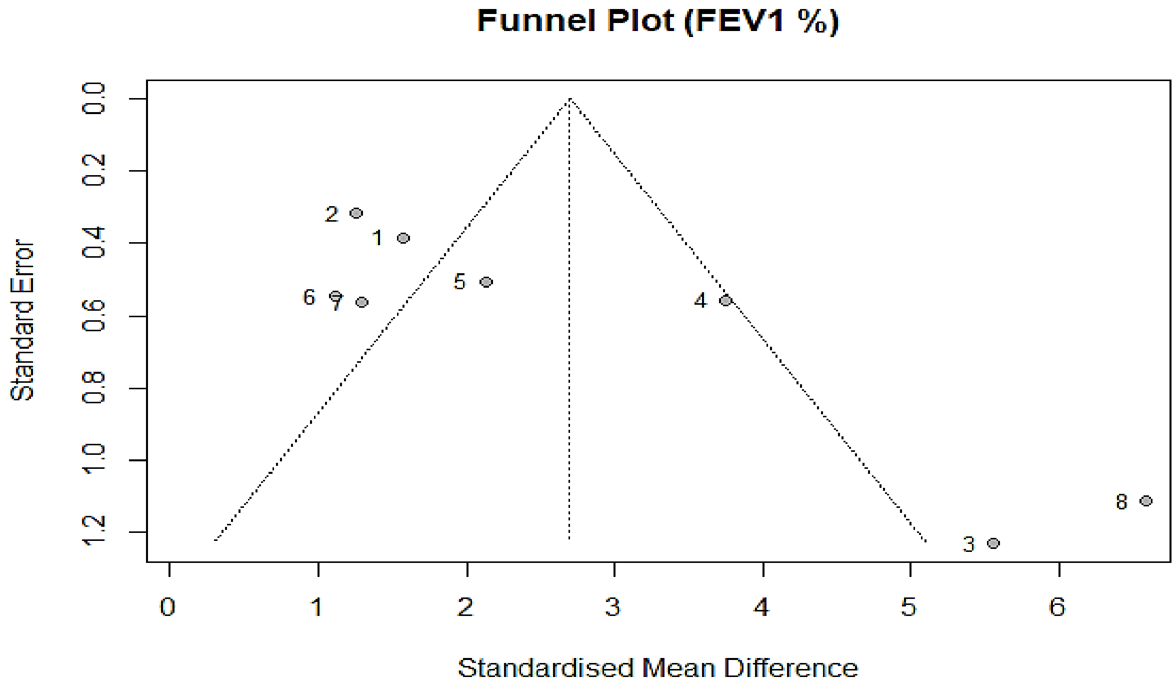


**Figure S1O: funnel plot showing publication bias for published studies regarding SMD of FEV1%.**

## S2. Forced expiratory volume at 1 second FEV1 (ml):

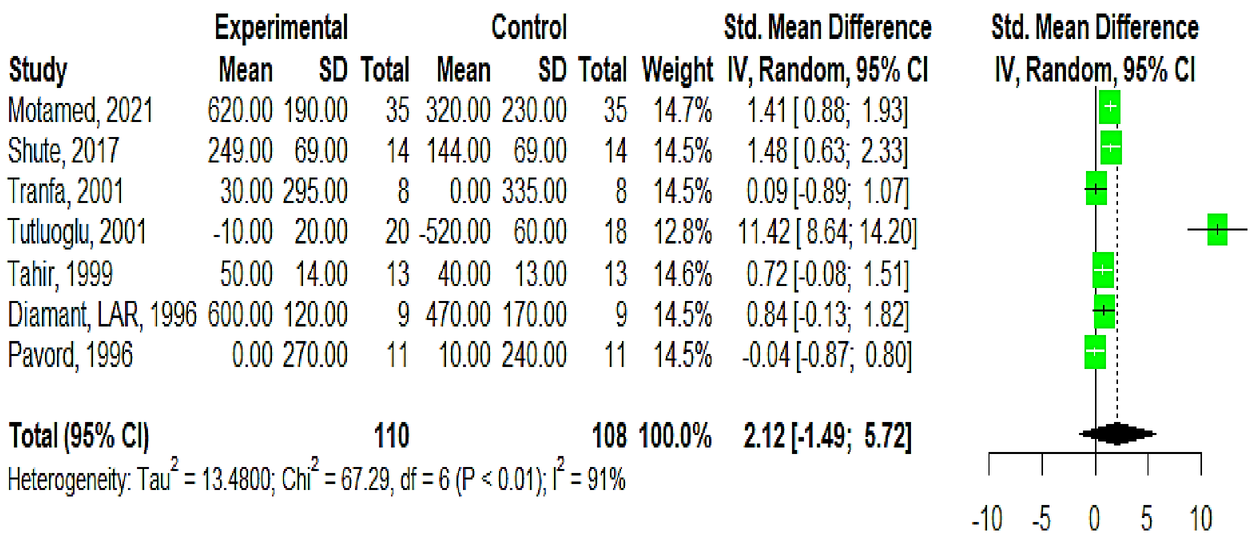

| Study | Experimental Mean | Experimental SD | Experimental Total | Control Mean | Control SD | Control Total | Weight | Std. Mean Difference IV, Random, 95% CI |
|---|---|---|---|---|---|---|---|---|
| Motamed, 2021 | 620.00 | 190.00 | 35 | 320.00 | 230.00 | 35 | 14.7% | 1.41 [0.88; 1.93] |
| Shute, 2017 | 249.00 | 69.00 | 14 | 144.00 | 69.00 | 14 | 14.5% | 1.48 [0.63; 2.33] |
| Tranfa, 2001 | 30.00 | 295.00 | 8 | 0.00 | 335.00 | 8 | 14.5% | 0.09 [-0.89; 1.07] |
| Tutluoglu, 2001 | -10.00 | 20.00 | 20 | -520.00 | 60.00 | 18 | 12.8% | 11.42 [8.64; 14.20] |
| Tahir, 1999 | 50.00 | 14.00 | 13 | 40.00 | 13.00 | 13 | 14.6% | 0.72 [-0.08; 1.51] |
| Diamant, LAR, 1996 | 600.00 | 120.00 | 9 | 470.00 | 170.00 | 9 | 14.5% | 0.84 [-0.13; 1.82] |
| Pavord, 1996 | 0.00 | 270.00 | 11 | 10.00 | 240.00 | 11 | 14.5% | -0.04 [-0.87; 0.80] |
| **Total (95% CI)** | | | **110** | | | **108** | **100.0%** | **2.12 [-1.49; 5.72]** |

Heterogeneity: $Tau^2 = 13.4800$; $Chi^2 = 67.29$, df = 6 ($P < 0.01$); $I^2 = 91\%$



**Figure S2A:** **Forest plot illustrates the pooled SMD of FEV1 ml.**

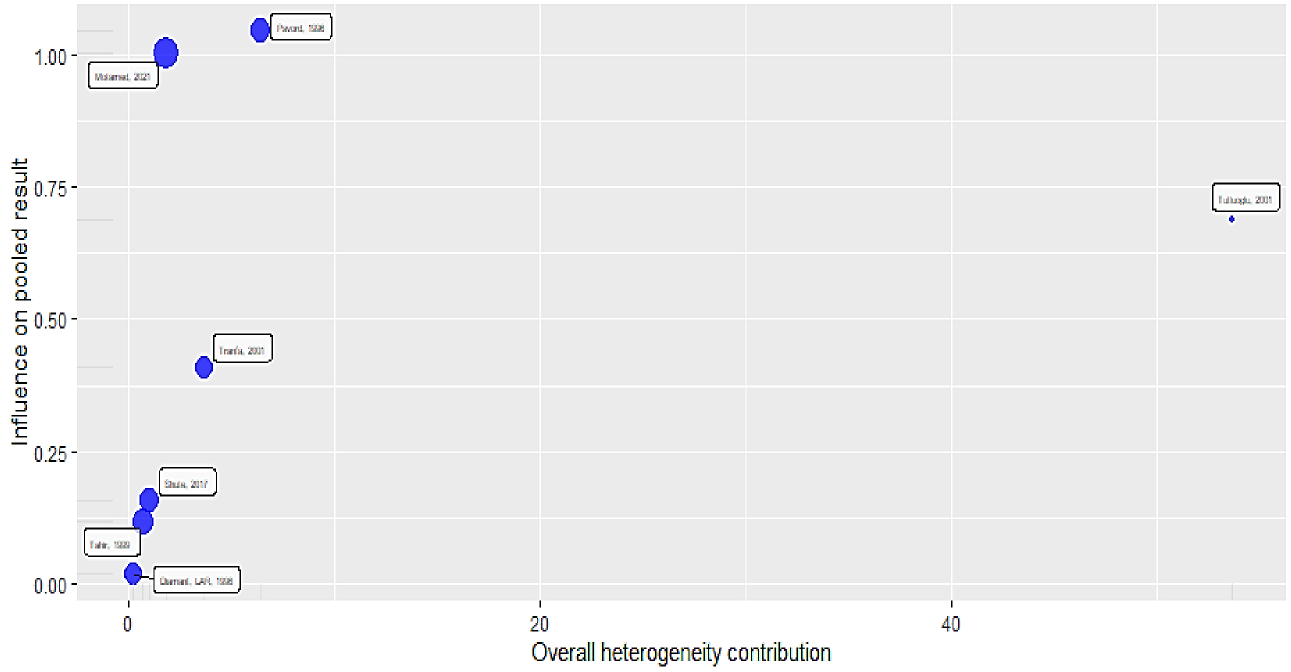


**Figure S2B: Baujat plot for FEV1ml checking for influential and outliers, showing contribution of each included study on the pooled effect and the overall heterogeneity.**

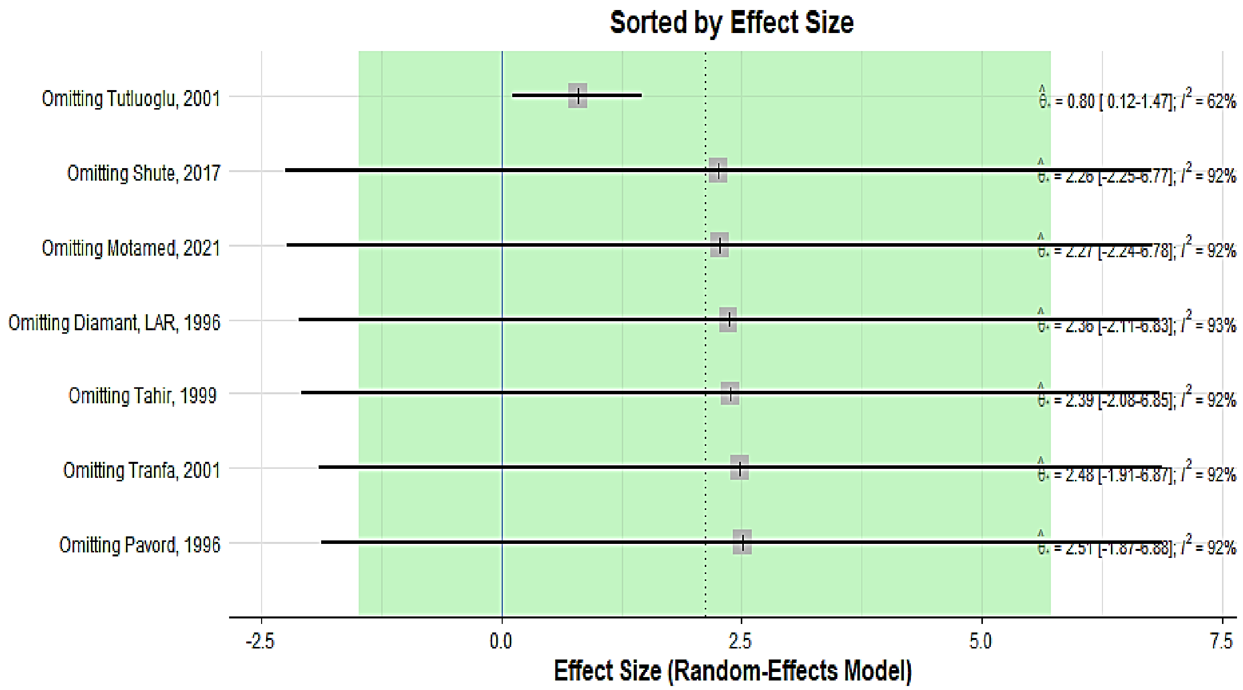


**Figure S2C: Diagram showing the net resulted pooled effect (SMD, CI) and heterogeneity $I^2$ of FEV1 ml after conducting leave one sensitivity analysis per study.**

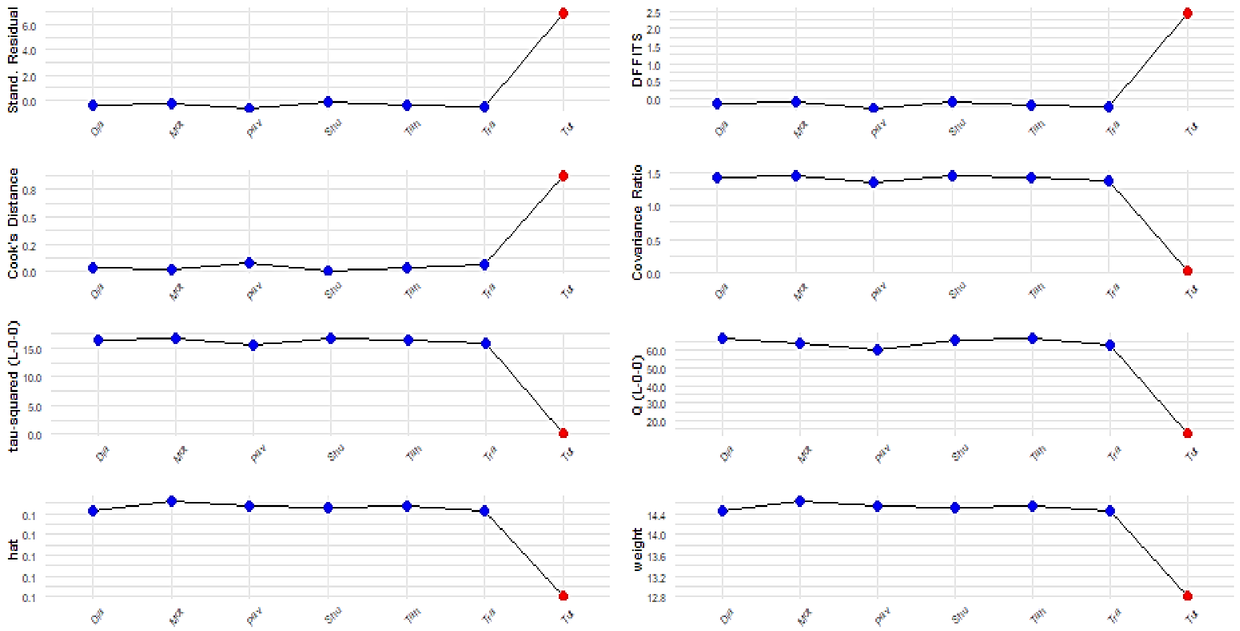


**Figure S2D: Diagram shows different calculated influence diagnostics of FEV1ml pooled estimate**

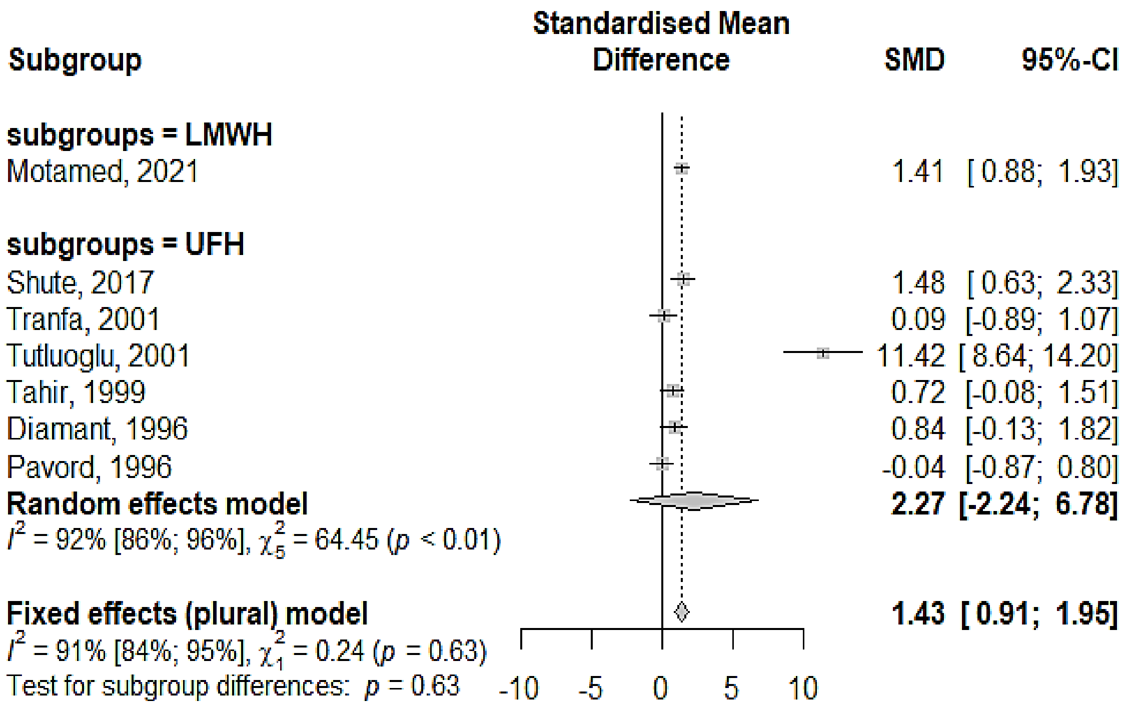


**Figure S2E: Subgroup analysis of FEV1 ml (SMD) by heparin type.**

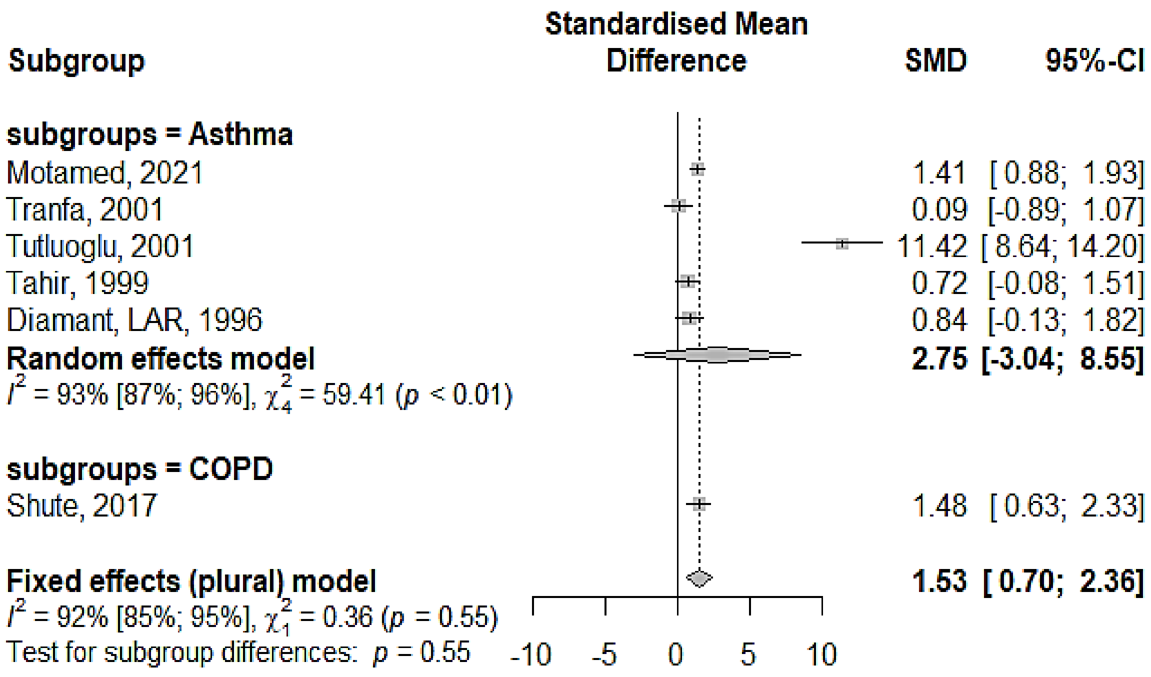


**Figure S2F: Subgroup analysis of FEV1 ml (SMD) by disease type.**

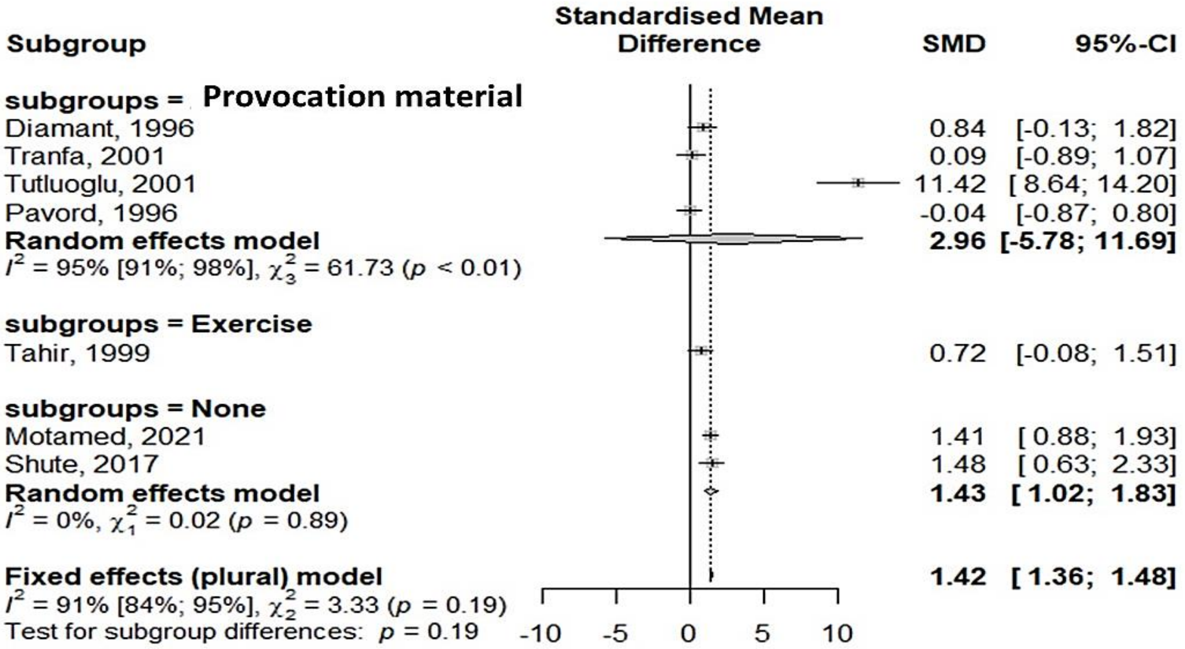


**Provocation material**: material used at provocation test either Methacholine, Histamine, Hypertonic potassium chloride, or Ultrasonically nebulized distilled water.

**Figure S2G: Subgroup analysis of SMD of FEV1 ml by Provocation test.**

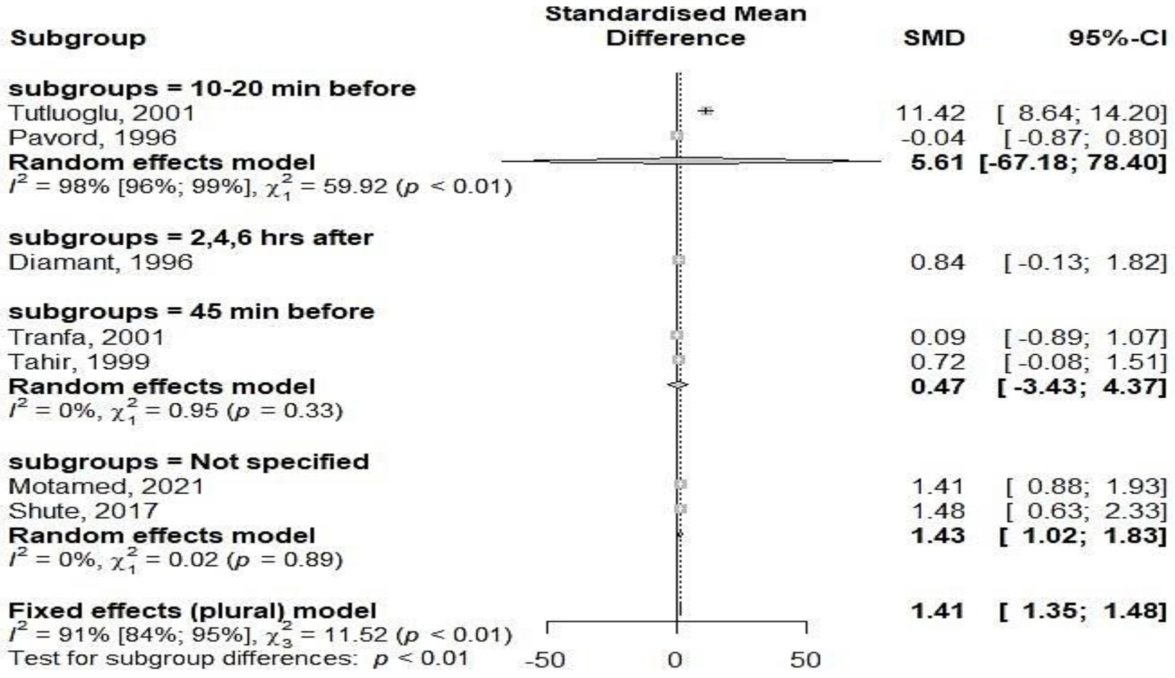


**Figure S2H: Subgroup analysis of FEV1 ml (SMD) by timing of heparin.**

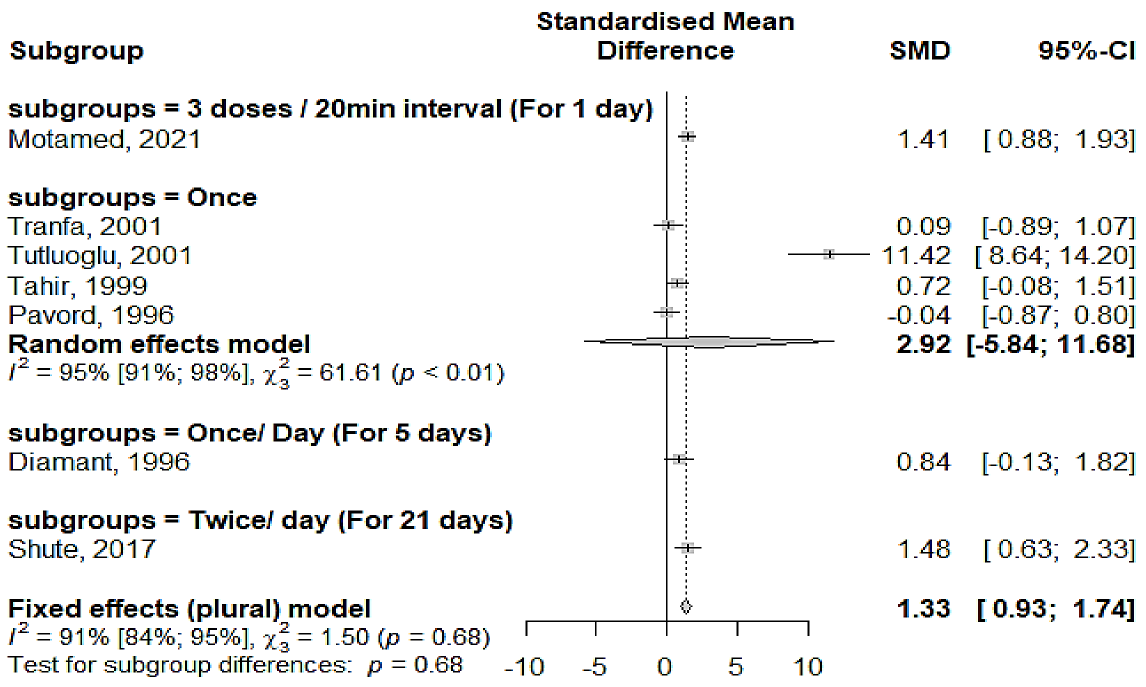


**Figure S2J: Subgroup analysis FEV1 ml (SMD) by frequency of using heparin.**

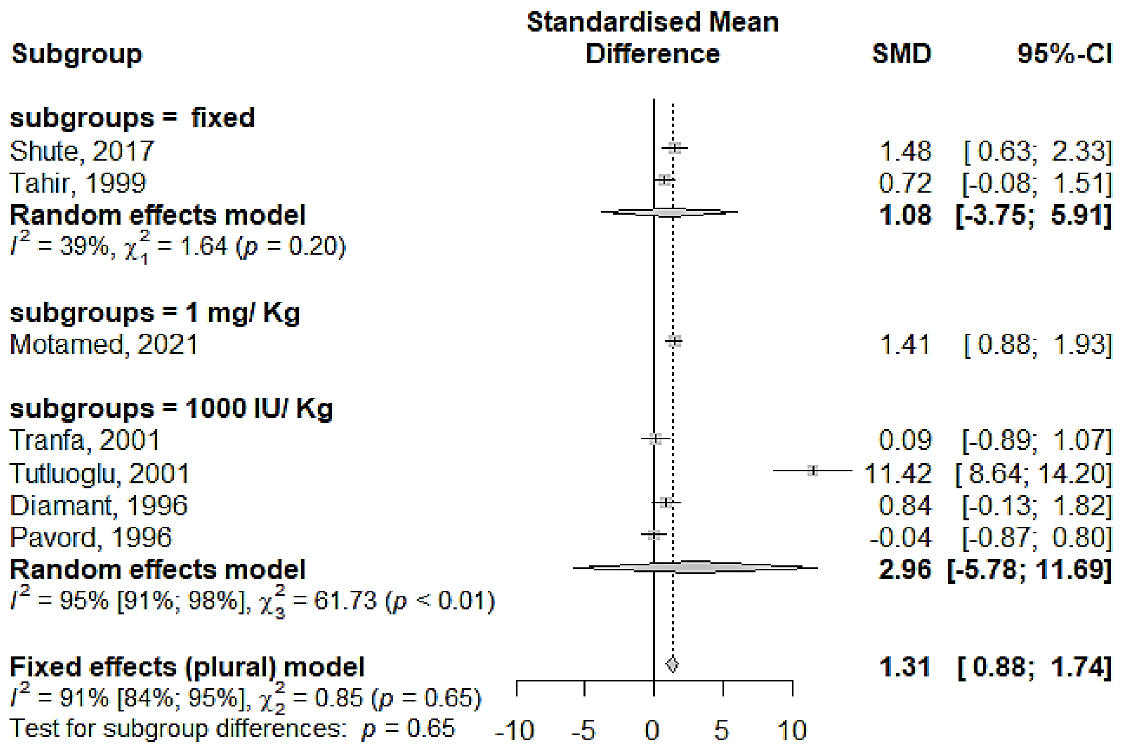


**Figure S2K: Subgroup analysis of FEV1 ml (SMD) by heparin dose.**

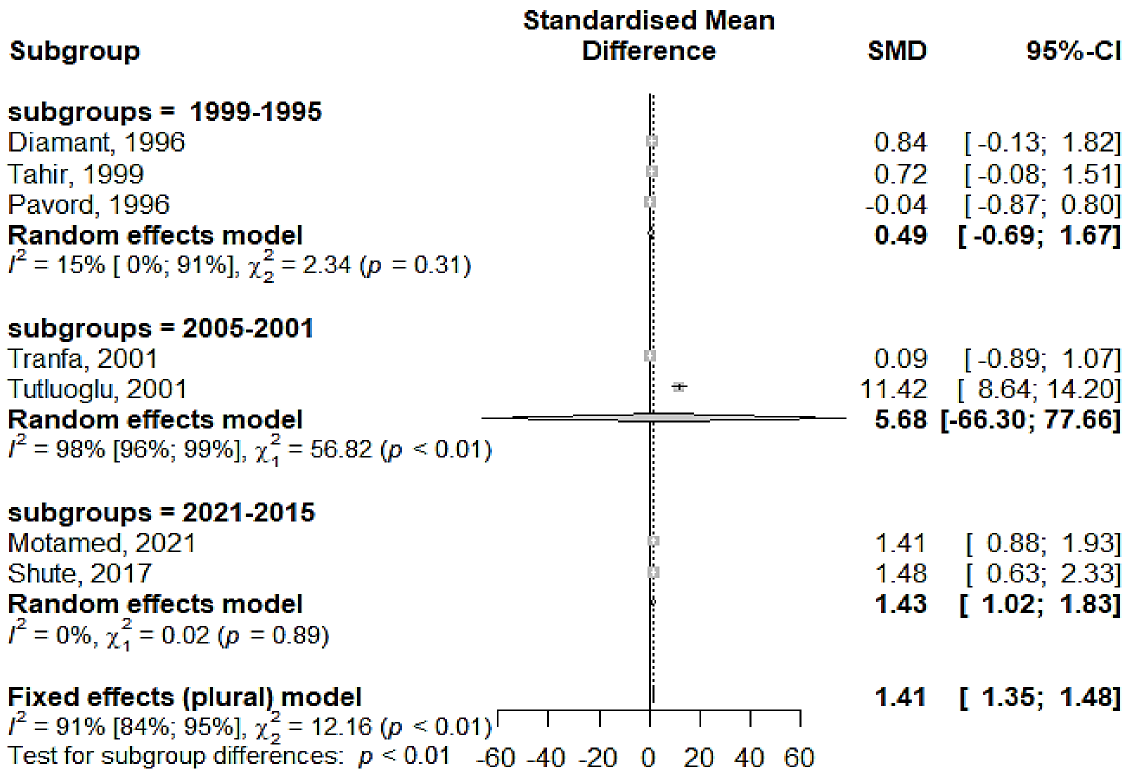


**Figure S2L: Subgroup analysis of FEV1 ml (SMD) by publication year category.**

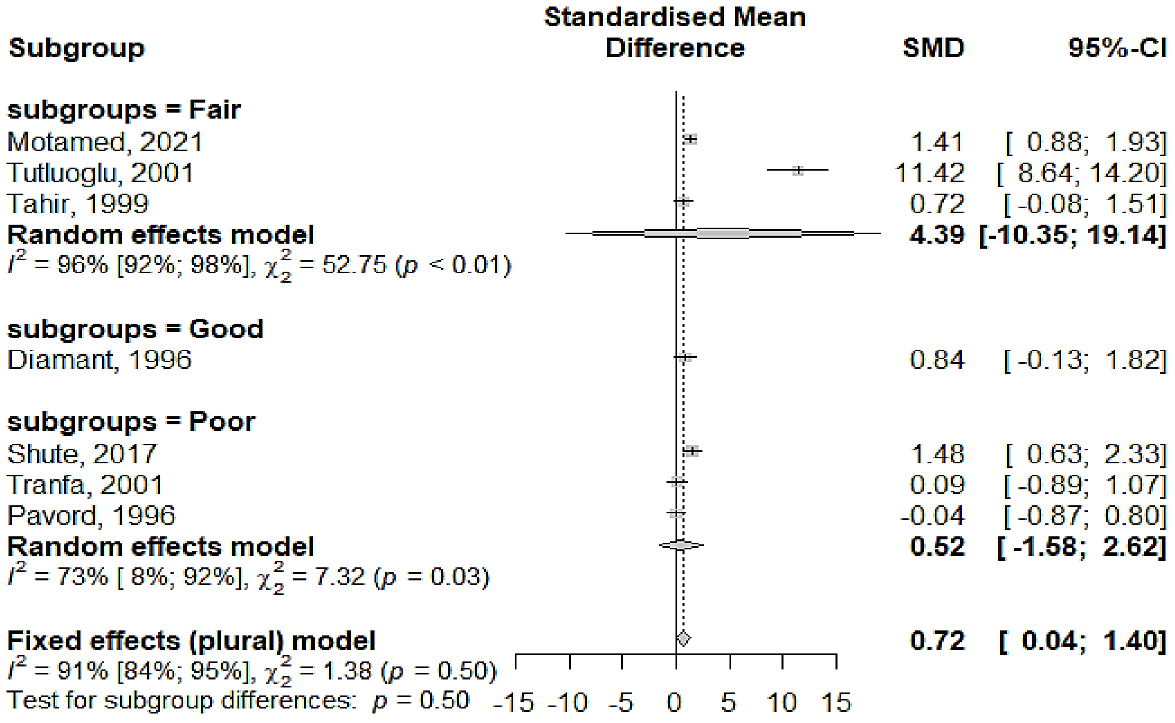


**Figure S2M: Subgroup analysis of FEV1 ml (SMD) by study quality.**

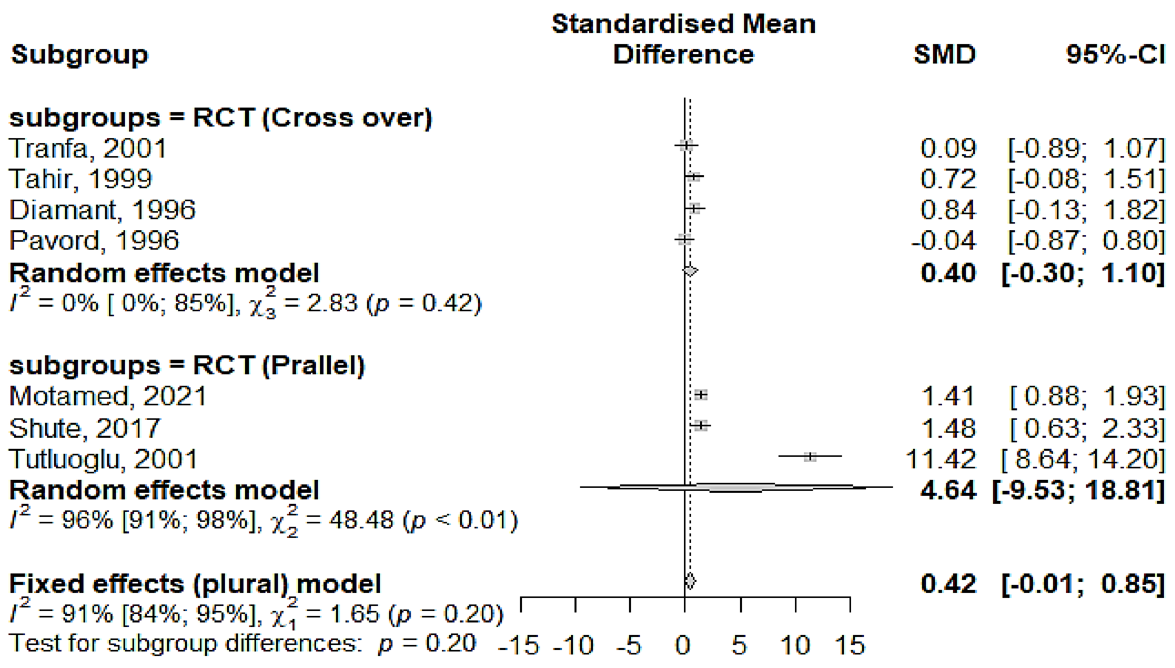


**Figure S2N: Subgroup analysis of FEV1 ml (SMD) by study design.**

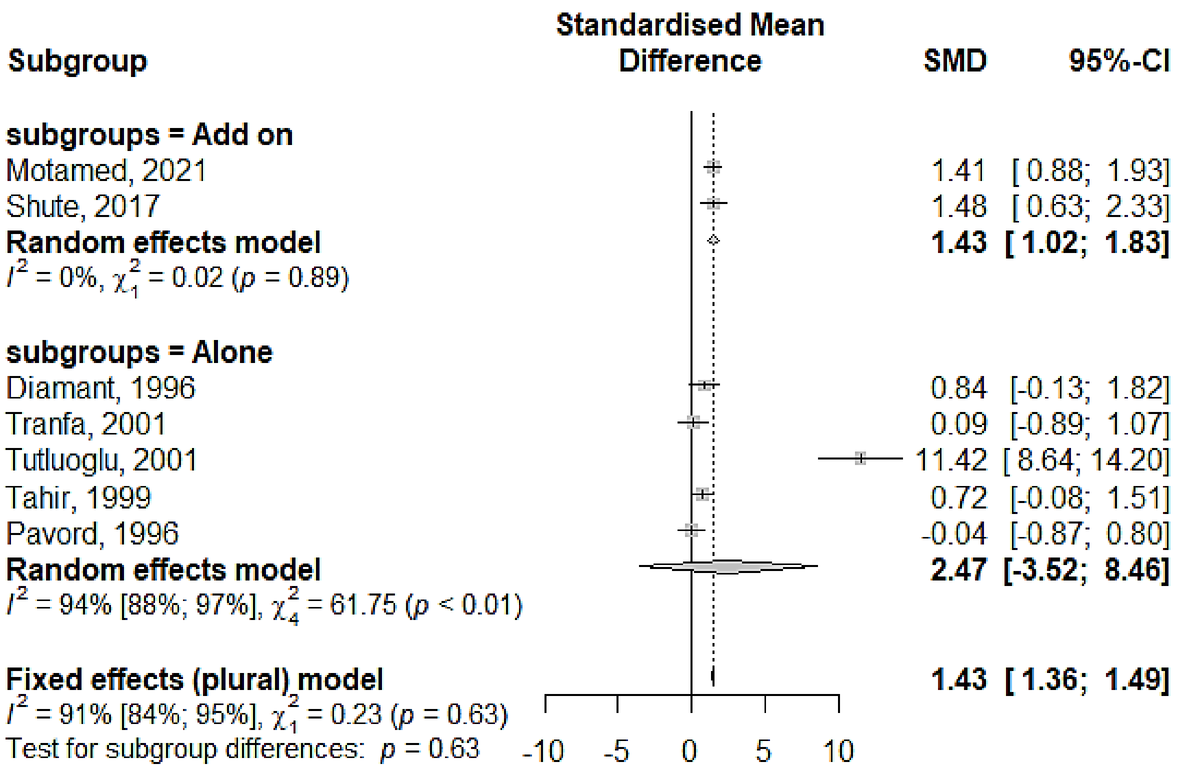


**Figure S2O: Subgroup analysis of FEV1 ml (SMD) by study Heparin treatment either used alone or add on to standard therapy.**

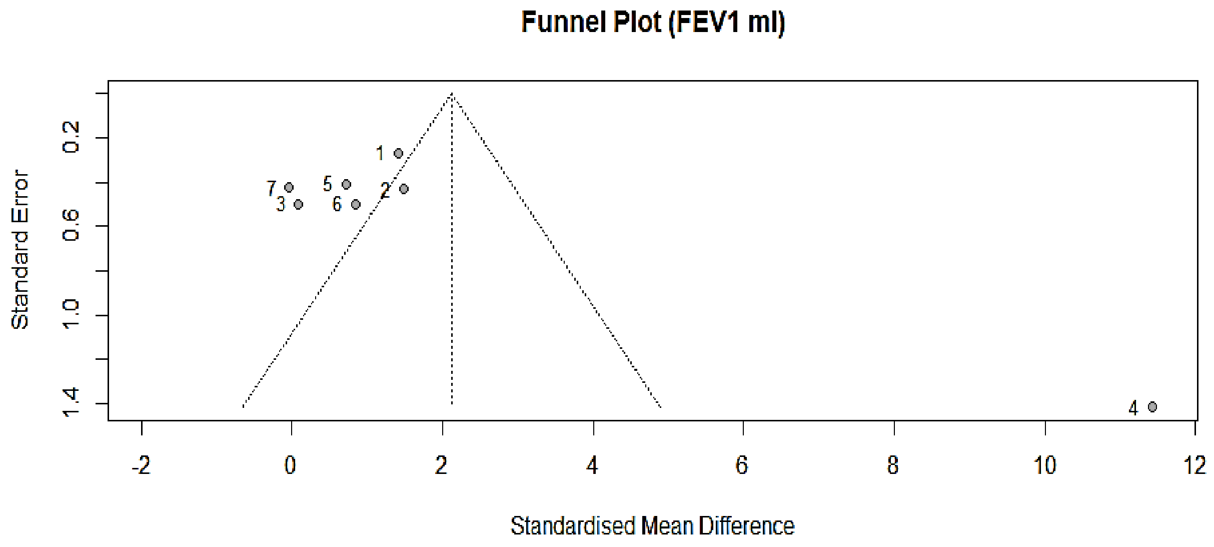


**Figure S2P: Funnel plot showing publication bias of published articles regarding FEV1(ml).**

## S3. Provocation concentration of allergen causing 20% fall of FEV1% (PC20) for asthmatic patients only:

| Study | Experimental Mean | Experimental SD | Experimental Total | Control Mean | Control SD | Control Total | Weight | Std. Mean Difference IV, Random, 95% CI |
|---|---|---|---|---|---|---|---|---|
| Doung, 2008 | 8.42 | 0.3000 | 18 | 9.70 | 0.3000 | 18 | 10.7% | -4.17 [-5.38; -2.96] |
| Ceyhan, 2000 | 6.17 | 1.5300 | 15 | 1.74 | 1.5300 | 15 | 11.0% | 2.82 [1.77; 3.86] |
| Lee, 1998 | 0.66 | 0.3130 | 8 | 0.25 | 0.0700 | 8 | 10.8% | 1.71 [0.52; 2.90] |
| Kalpaklioglu, 1997 | 6.45 | 1.8000 | 12 | 4.64 | 1.3500 | 12 | 11.2% | 1.10 [0.23; 1.97] |
| Polosa, 1997 | 0.12 | 17.9000 | 17 | 42.50 | 37.3000 | 17 | 11.4% | -1.41 [-2.18; -0.65] |
| Ceyhan, 1997 | 97.70 | 33.6000 | 16 | 38.90 | 33.6000 | 16 | 11.3% | 1.71 [0.88; 2.53] |
| Pavord, 1996 | 1.51 | 1.2500 | 12 | 1.00 | 1.2500 | 12 | 11.3% | 0.39 [-0.42; 1.20] |
| Diamant, 1996 | 1.50 | 1.2800 | 8 | 1.27 | 0.7500 | 8 | 11.1% | 0.21 [-0.78; 1.19] |
| Ceyhan, 1995 | 10.57 | 5.7200 | 13 | 5.26 | 4.8000 | 13 | 11.3% | 0.97 [0.15; 1.79] |
| **Total (95% CI)** | | | **119** | | | **119** | **100.0%** | **0.38 [-1.20; 1.95]** |

Heterogeneity: $Tau^2 = 3.8758$; $Chi^2 = 116.02$, df = 8 ($P < 0.01$); $I^2 = 93\%$

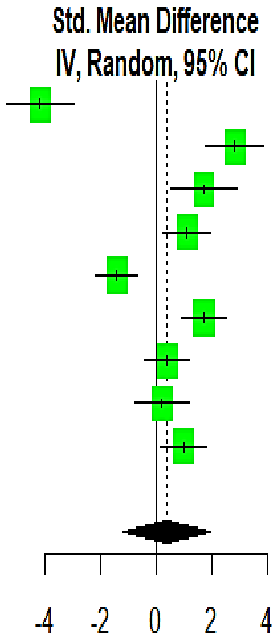


**Figure S3A: Forest plot illustrates the pooled SMD of PC20.**

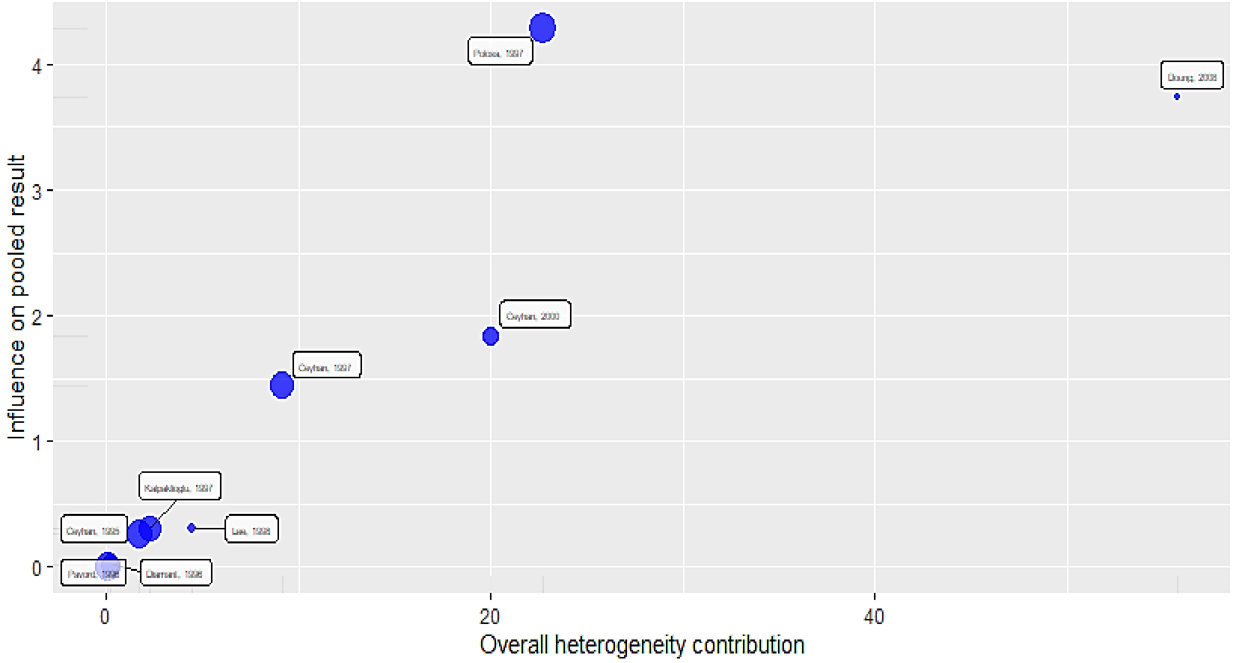


**Figure S3B:** **Baujat plot for PC20 checking for influential and outliers, showing contribution of each included study on the pooled effect and the overall heterogeneity.**

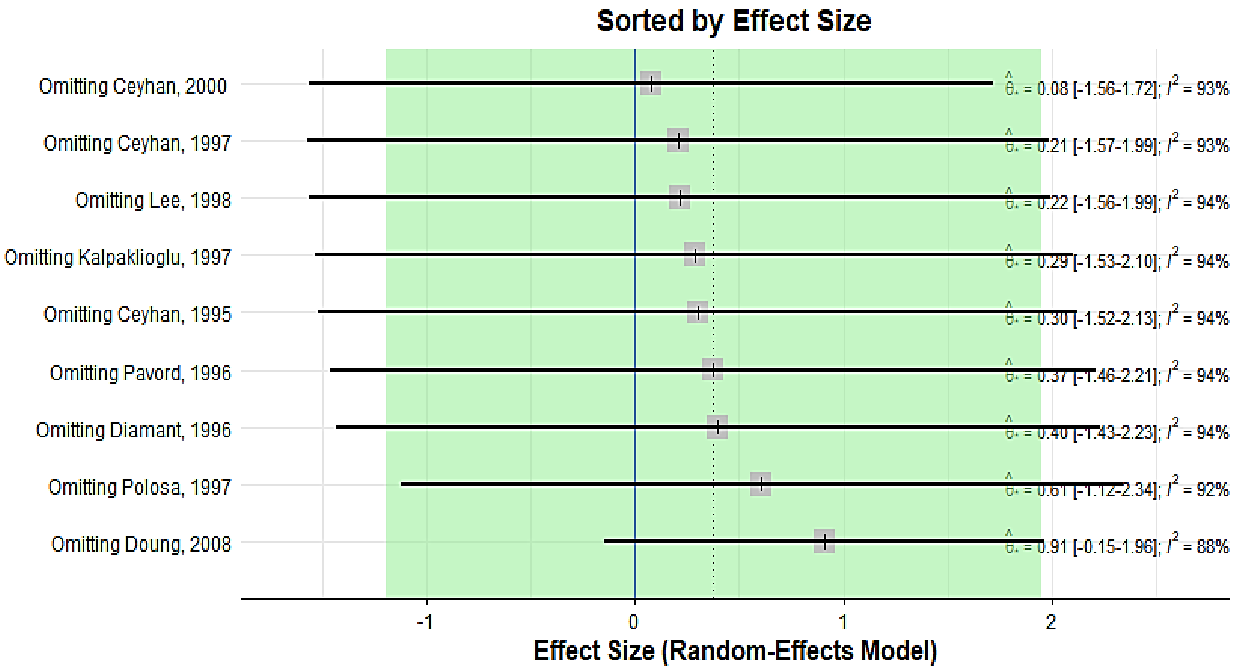


**Figure S3C: Diagram showing the net resulted pooled effect (SMD, CI) and heterogeneity $I^2$ of PC20 after conducting leave one sensitivity analysis per study.**

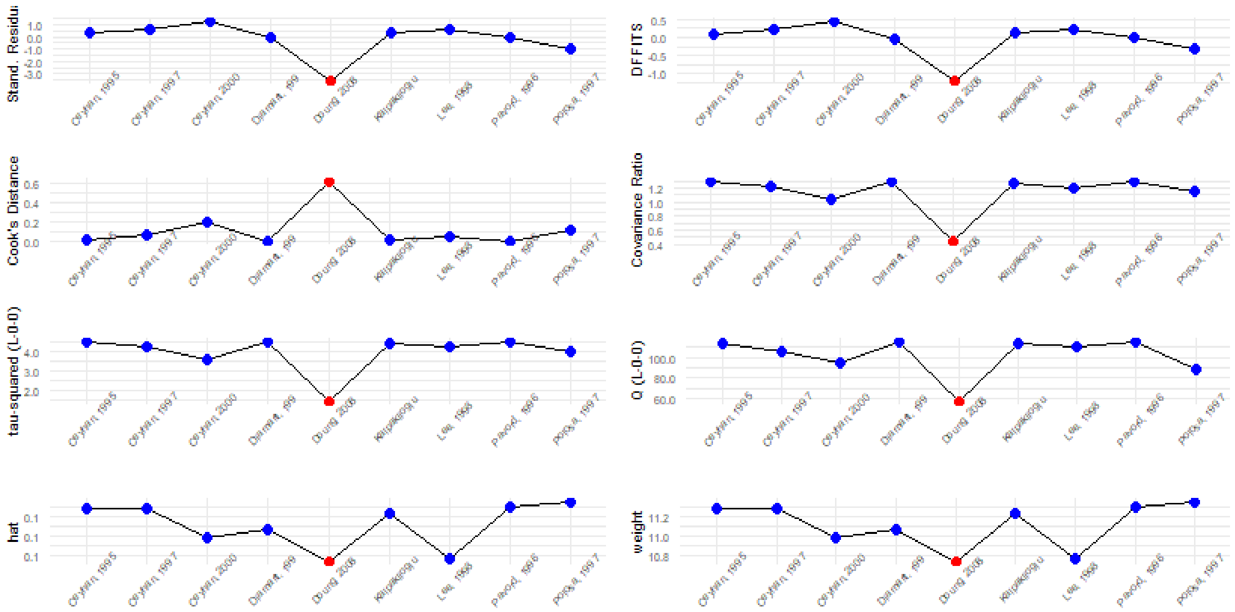


**Figure S3D: Diagram shows different calculated influence diagnostics affecting PC20 SMD.**

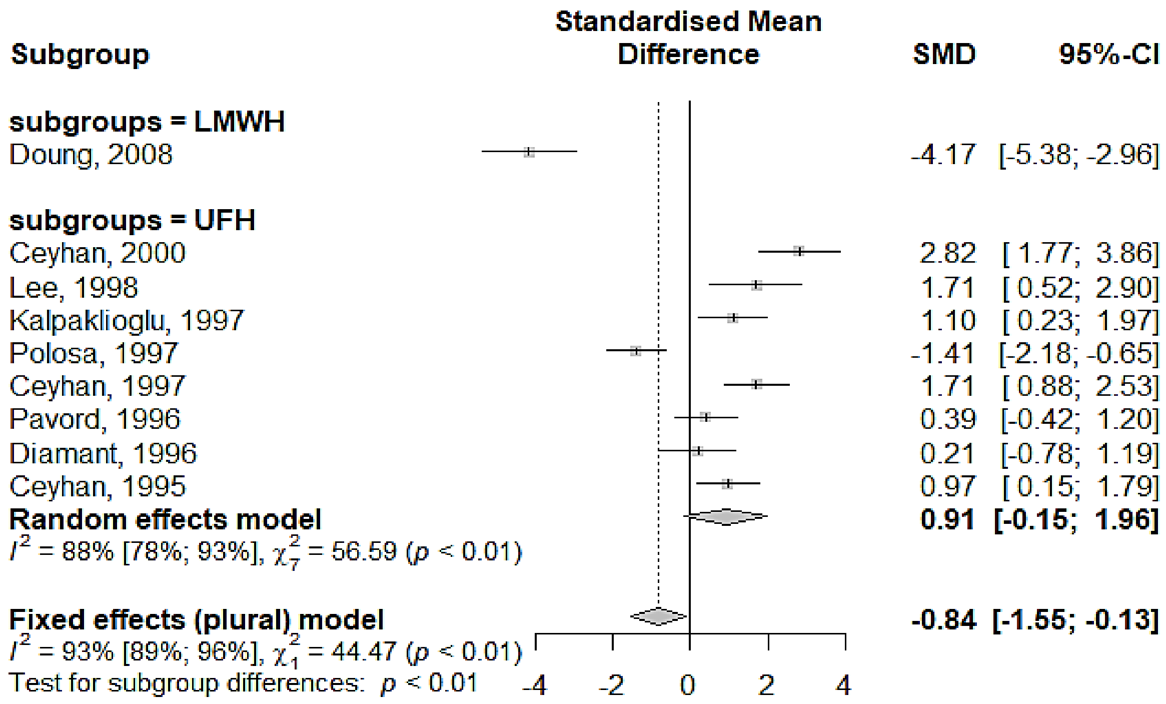


**S3E: Subgroup analysis of PC20 (SMD) by heparin type.**

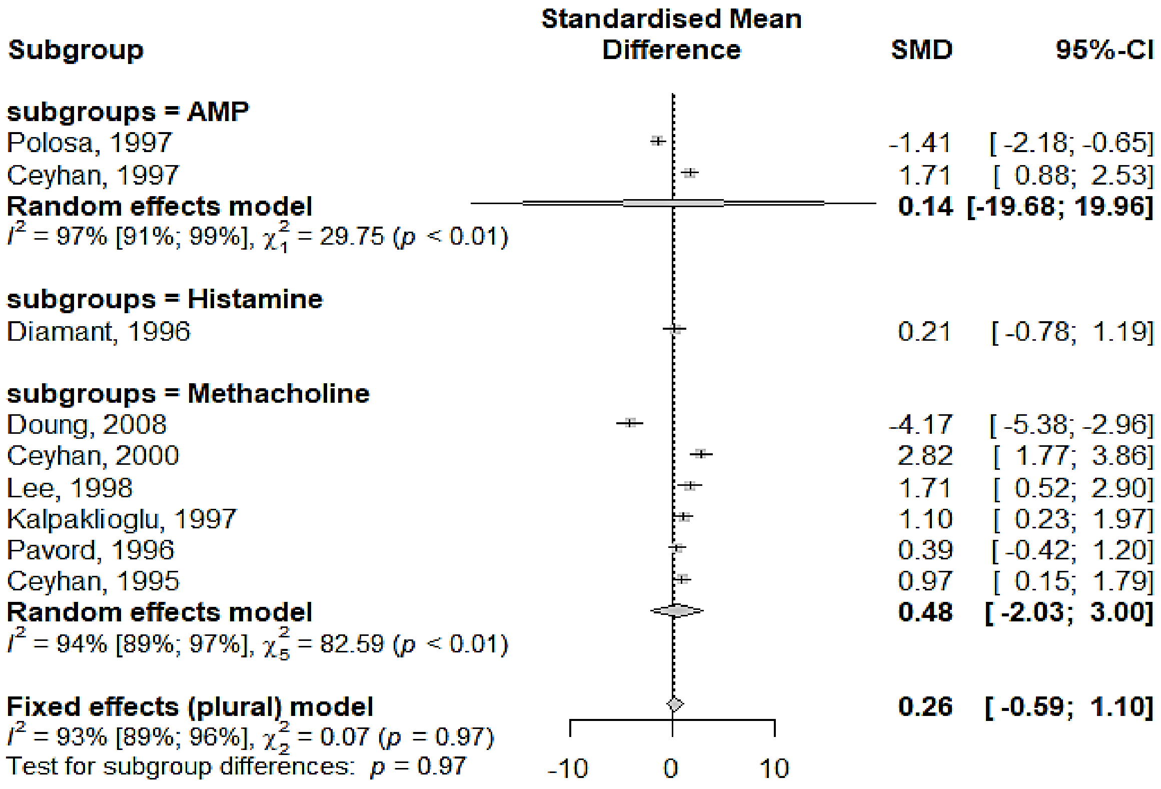


**Figure S3F: Subgroup analysis of SMD of PC20 by provocation test material used).**

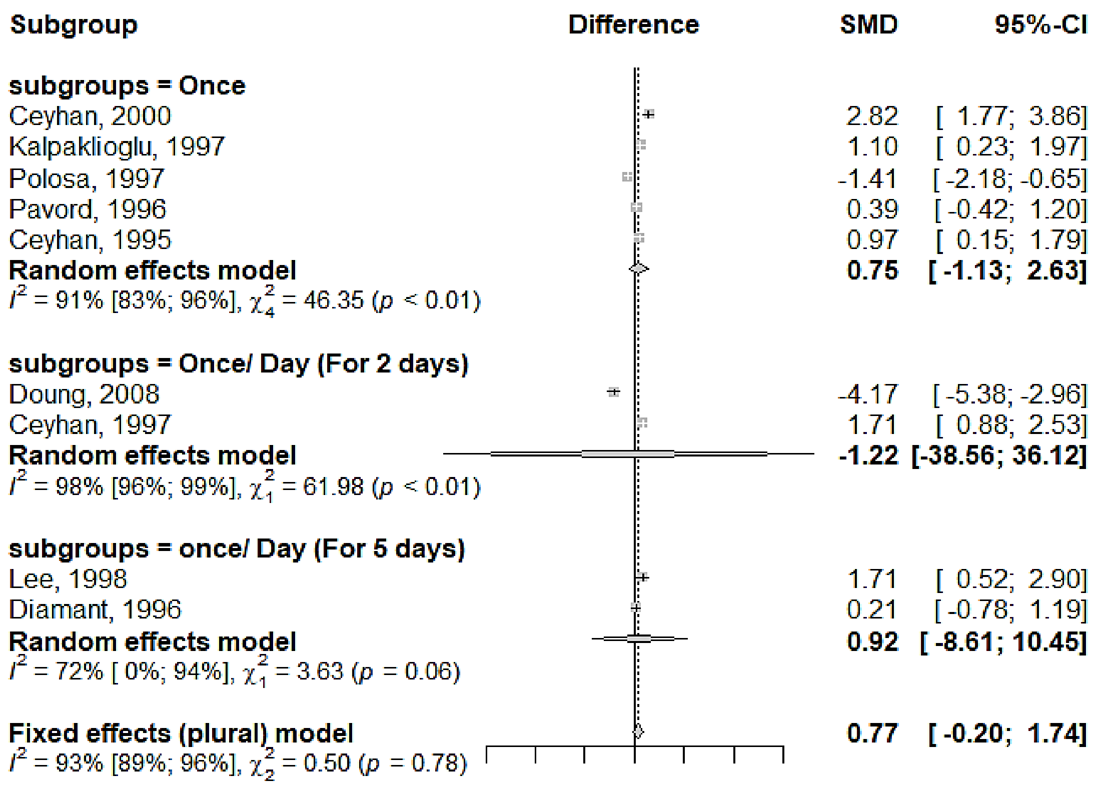


**S3G: subgroup analysis of SMD of PC20 by Frequency of heparin.**

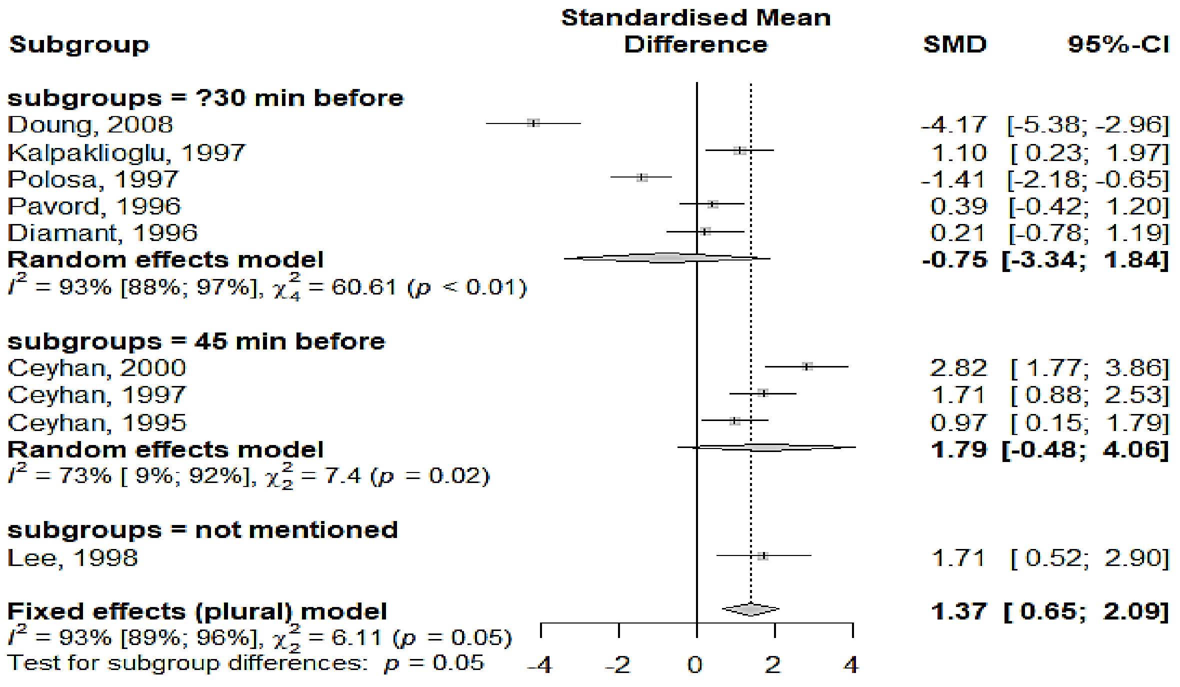


**S3H: Subgroup analysis of SMD of PC20 by the timing of heparin** (? 30 min = <30 minutes

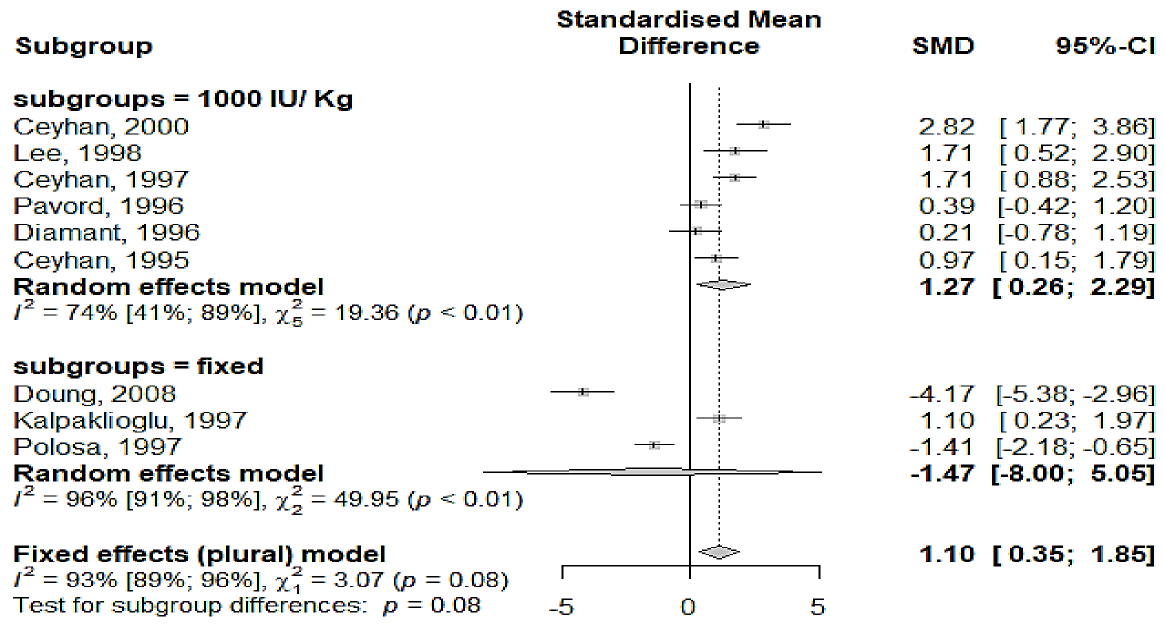


**S3J: Subgroup analysis of SMD of PC20by heparin dose**

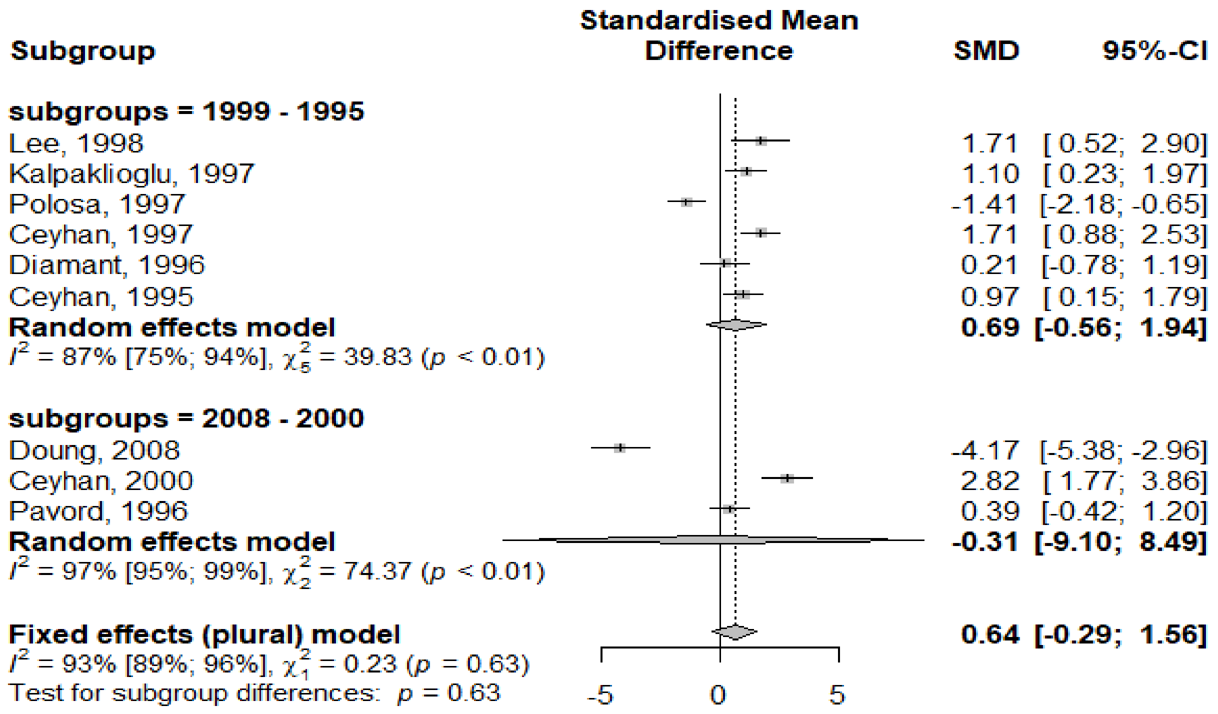


**S3K: Subgroup analysis of SMD of PC20 by publication year category.**

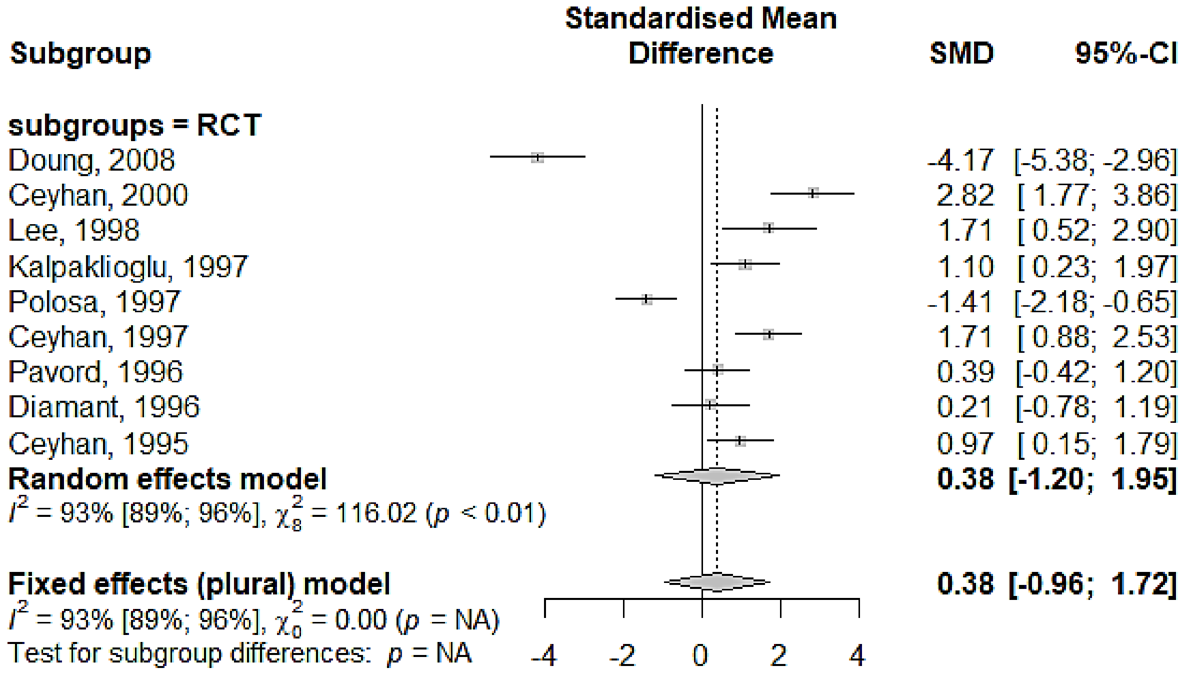


**Figure S3L: Subgroup analysis of SMD of PC20 by study design**

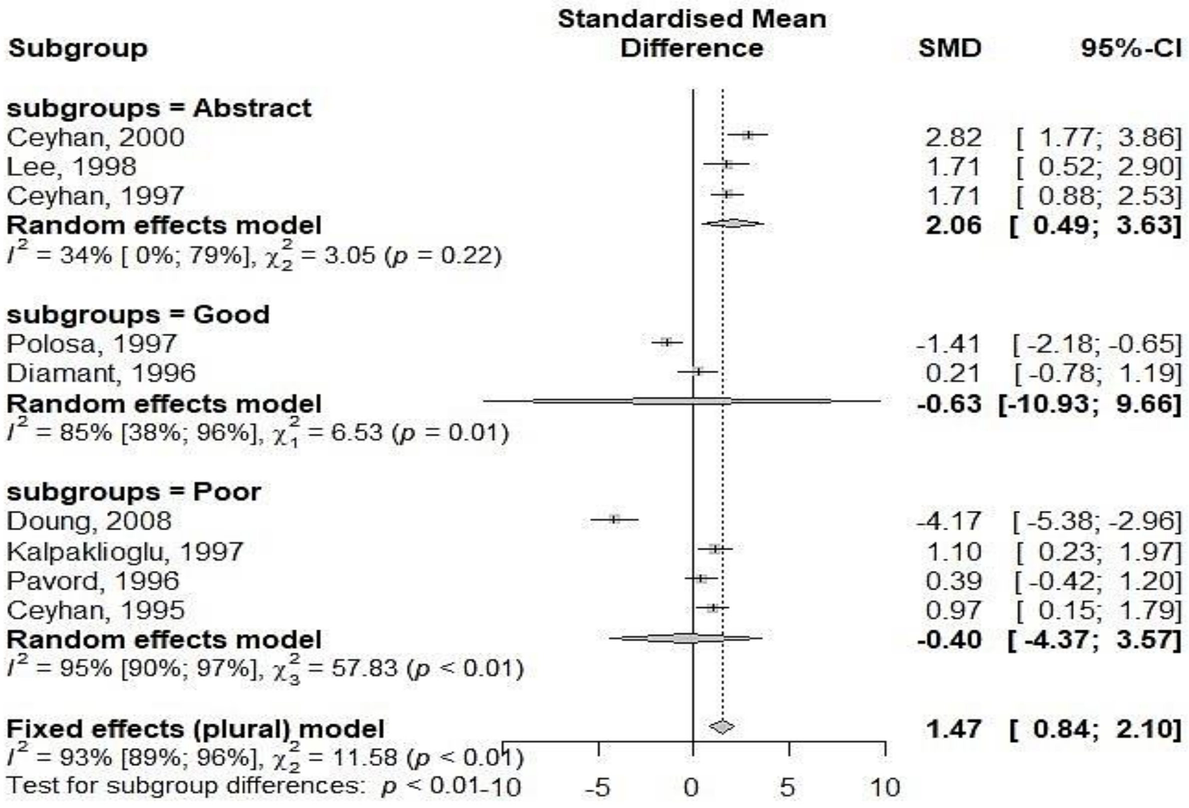


**Figure S3L: Subgroup analysis of SMD of PC20 by study quality.**

# Appendix 3

**Author(s):** Rasha Ashmawy, Iman El Sayed, Adel Zaki, Ayman Baess
**Question:** Inhaled heparin compared to Placebo or SC for Asthma or COPD
**Setting:**
**Bibliography:**

| Certainty assessment | | | | | | | № of patients | | Effect | | Certainty | Importance |
|---|---|---|---|---|---|---|---|---|---|---|---|---|
| № of studies | Study design | Risk of bias | Inconsistency | Indirectness | Imprecision | Other considerations | Inhaled heparin | Placebo or SC | Relative (95% CI) | Absolute (95% CI) | | |
| **FEV1 meam diffrence (ml)** | | | | | | | | | | | | |
| 7 | randomised trials | serious | serious | not serious | not serious | publication bias strongly suspected strong association dose response gradient | 110 | 108 | - | SMD **1.64 higher** (0.51 higher to 2.76 higher) | ⨁⨁⨁◯ Moderate | |
| **FEV1%** | | | | | | | | | | | | |
| 8 | randomised trials | serious | not serious | not serious | not serious | strong association dose response gradient | 111 | 109 | - | SMD **2.34 higher** (1.5 higher to 3.18 higher) | ⨁⨁⨁⨁ High | |
| **PC20** | | | | | | | | | | | | |
| 9 | randomised trials | serious | not serious | not serious | not serious | strong association dose response gradient | 120 | 114 | - | SMD **0.64 higher** (0.34 higher to 0.94 higher) | ⨁⨁⨁⨁ High | |

**CI:** confidence interval; **SMD:** standardised mean difference

## GRADE Table before sensitivity analysis

**Author(s):**
**Question:** Heparin compared to Control for Asthma or COPD
**Setting:**
**Bibliography:**

| Certainty assessment | | | | | | | № of patients | | Effect | | Certainty | Importance |
|---|---|---|---|---|---|---|---|---|---|---|---|---|
| № of studies | Study design | Risk of bias | Inconsistency | Indirectness | Imprecision | Other considerations | Heparin | Control | Relative (95% CI) | Absolute (95% CI) | | |
| **FEV1 meam diffrence (ml)** | | | | | | | | | | | | |
| 6 | randomised trials | serious | serious | not serious | not serious | strong association all plausible residual confounding would reduce the demonstrated effect dose response gradient | 90 | 90 | - | SMD **0.8 higher** (0.27 higher to 1.32 higher) | ⊕⊕⊕⊕ High | IMPORTANT |
| **FEV1%** | | | | | | | | | | | | |
| 7 | randomised trials | serious | serious | not serious | not serious | strong association all plausible residual confounding would reduce the demonstrated effect dose response gradient | 99 | 97 | - | SMD **2.09 higher** (1.28 higher to 2.9 higher) | ⊕⊕⊕⊕ High | IMPORTANT |
| **PC20** | | | | | | | | | | | | |
| 8 | randomised trials | serious | serious | not serious | not serious | strong association all plausible residual confounding would reduce the demonstrated effect dose response gradient | 102 | 96 | - | SMD **0.96 higher** (0.65 higher to 1.27 higher) | ⊕⊕⊕⊕ High | IMPORTANT |

**CI:** confidence interval; **SMD:** standardised mean difference

# GRADE Table after sensitivity analysis.

# Appendix 4

## S4: Forest plot of FVC reported studies.

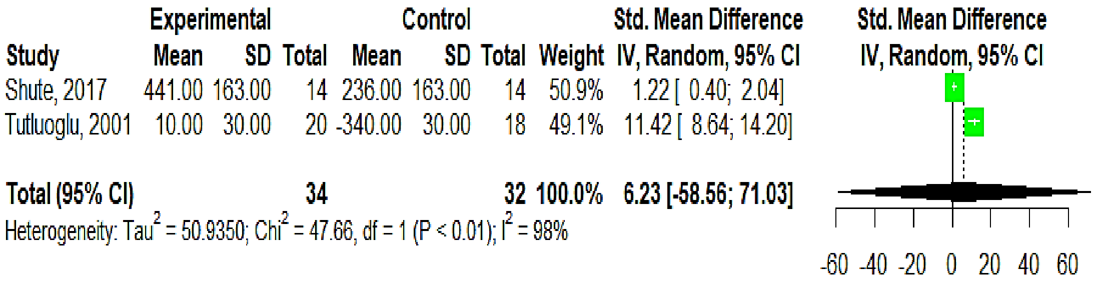


## S5: Forest plot of PEFR reported studies.

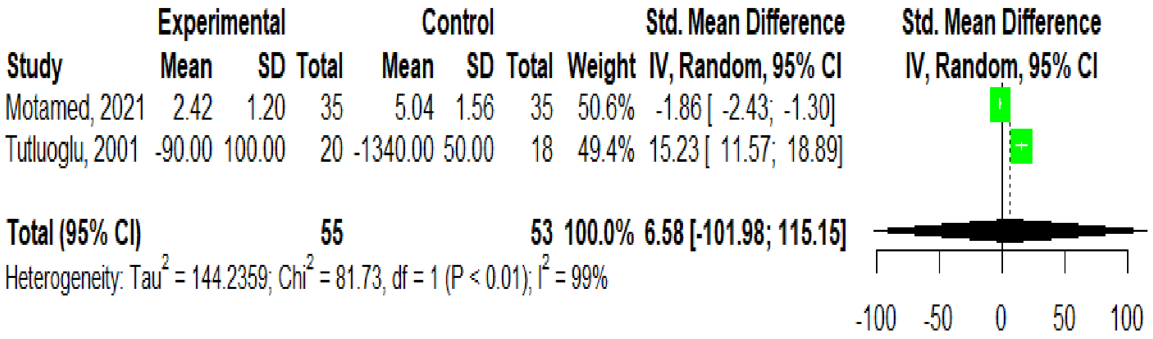


## S6: Forest plot of AUC reported studies.

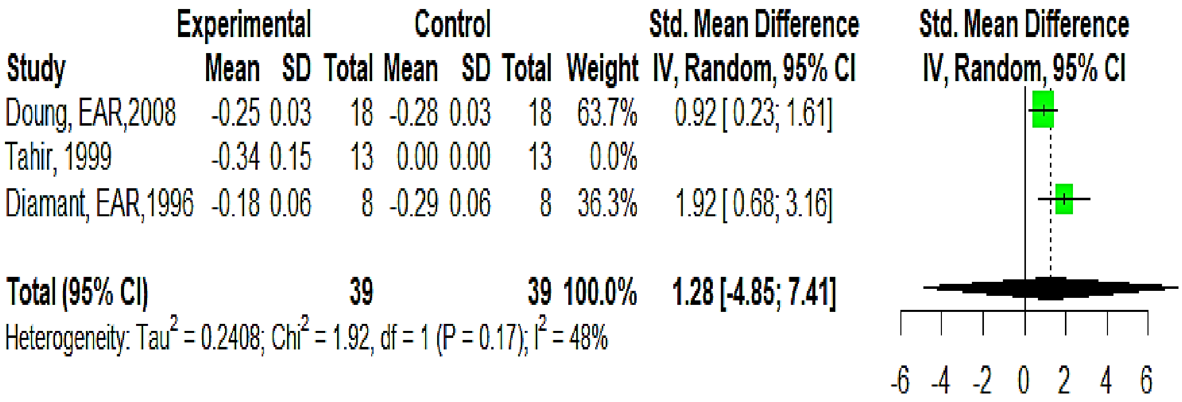

# Appendix 5
# Narrative synthesis of included studies.

| No | Author, year, country | Population | Study design | Inclusion criteria. | Heparin timing | Type of heparin and device | Comparator | Allergen used | Outcomes |
|---|---|---|---|---|---|---|---|---|---|
| 1 | Pavord, 1996, UK | - Adults<br><br>- Mild asthmatic<br><br>- Mean age 34 y.<br><br>- N=11<br><br>- 73% male | RCT, double blind, cross over 4 times | -Mild stable asthma<br><br>- FEV1> 60% predicted<br><br>-Stop bronchodilator medication 6 h before each visit.<br><br>-Non-smokers.<br><br>-CS not sopped. | - once<br><br>-10 minutes before provocation test for each allergen. | Heparin sodium (1000 U/Kg) max 80,000<br><br>Jet nebulizer (output 0.2 mL·min-1) | Placebo (N.S.) | -Sodium metabisulphite<br><br>-Methacholine | - No significant change in FEV1.<br><br>- No significant effect of dose response curve of the two allergens (p= 0.67, 0.21).<br><br>- inhaled heparin is well tolerated.<br><br>- No prolongation in ATTP. |
| 2 | Ceyhan, 1995, Turkey | - Adults<br><br>- Mild asthmatic.<br><br>- N= 13<br><br>- 53.8% male | RCT, single-blind, crossover | -Low frequency of symptoms (≤ 2 attack/week).<br><br>-FEV1 >80% predicted<br><br>-Not requiring regular therapy except for short periods of time.<br><br>-No acute attack or respiratory tract infection within the | - once<br><br>-45 minutes before provocation test. | -Undiluted commercial heparin sodium (1000 U/Kg) max 60,000<br><br>- Administered as a constant-flow aerosol during tidal breathing.<br><br>-The nebulizer provided an aerosol with a | Placebo | Methacholine | -Heparin inhibits bronchoconstrictio n induced by methacholine.<br><br>-Heparin increases the mean PD20 over placebo (p< 0.0002)<br><br>-8/13 patient reported headache due to heparin |

| | | | | | | | | | |
|---|---|---|---|---|---|---|---|---|---|
| | | | | last 3 months.<br><br>-Nonsmoker<br><br>- Bronchodilator stopped 24 hours before each visit. | | mass median aerodynamic diameter of 1 to 4 !Lm (Voyage nebulizer, Mefar, Italy). | | | inhalation and resolved at 1-2 hours.<br><br>-1 patient withdrawn due to acute bronchospasm after heparin inhalation. |
| 3(a ) | Lee, 1998, Korea | -Adults<br><br>-Exercise-induced asthma (EIA).<br><br>-Mean age 31.5 y<br><br>-N =8<br><br>-62.5% male | not mentioned | Exercise induced asthmatic patients | -Once for subsequent five days before exercise. | -Heparin 1000unit/Kg/day | -Control | Methacholine | -Heparin has protective effect on EIA.<br><br>- heparin prevents FEV1 decrease after exercise than control (p = 0.01)<br><br>-Heparin not significantly increases methacholine PD20 than control at day 3 (p>0.05)<br><br>-No effect on anticoagulation (aPTT). |
| 4 | Kalpaklioglu, 1997, Turkey | -Adults<br><br>-Mild | RCT, double blind, cross | -Stable patients with strong history of allergic | -Once<br><br>-20-30 minutes | -Commercial heparin sodium administered at a | Placebo (N.S.) | Methacholine | -Heparin causes a significant increase in PC20 |

| | | | | | | | | | |
|---|---|---|---|---|---|---|---|---|---|
| | | asthmatic patients<br><br>-Mean age 31.8 y<br><br>-N =12<br><br>-25% male | over. | asthma/rhinitis.<br><br>-Documented positive bronchoconstriction response to methacholine.<br><br>-No clotting problems.<br><br>Free of RTI or exacerbation of the symptoms within 3 months before entering the study.<br><br>-Inhaled B2-agonists and caffeine-containing foods and beverages were withheld for 24 hours, antihistamines for 6 weeks before the study day | before provocation test. | fixed dose of 20,000 U.<br><br>-Pari nebulizer.<br><br>-Constant flow aerosol during tidal breathing. | | | methacholine compared to placebo ($p<0.05$)<br><br>-Heparin didn't prevent the increase in airway resistance (Raw). ($p<0.05$)<br><br>-Heparin didn't prevent decrease in specific conductance (SGaw). ($p>0.05$)<br><br>-Side effects: Not mentioned |
| 5 | Duong, 2008,Canada | -Adults<br><br>-Mild atopic asthmatics<br><br>-Mean age | RCT, double-blind, placebo-controlled, double- | -Stable asthma (for 6 weeks prior to screening)<br><br>-FEV1 ≥70% | -Single dose<br><br>-30 minutes before allergen' | -Nebulized IVX-0142 (80 mg in 4 ml of sterile water)<br><br>- | Placebo | Exhaled nitric oxide (eNO), methacholine test. | -Treatment with IVX-0142 showed a trend toward attenuation of the EAR (maxFEV1% fall 26.5 ± 2.8% vs |

| | | | | | | | | | | |
|---|---|---|---|---|---|---|---|---|---|---|
| | | **29.7 y**<br><br>**-N= 18**<br><br>**-21% male** | **dummy, two periods cross-over study.** | **predicted.**<br><br>**- Nonsmoker.** | **EAR: max fall in FEV1 at 0-3 hrs post allergen**<br><br>**LAR : max fall in FEV1 at 3-7 hrs post allergen** | **nonanticoagulate d, porcine heparin-derived hexa-sulfated disaccharide** | | | **placebo 31.0 ± 2.8%, P = 0.059)**<br><br>**-The LAR (15.6 ± 2.9% placebo 19.0 ± 2.9%, P = 0.24).**<br><br>**-No significant effect on the allergen-induced changes in methacholine-PC20 (P = 0.2).**<br><br>**-significant rise in absolute TCC, eosinophils and neutrophil count 7 hours after allergen in placebo arm only.**<br><br>**-one subject was suffering from a**<br><br>**Self-limited mild headache**<br><br>**-No major adverse events were reported**<br><br>**-no significant change in the measured coagulation Factors.** |

| | | | | | | | | | |
|---|---|---|---|---|---|---|---|---|---|
| 6 | Polosa, 1997, Italy | -Adults<br><br>-Asthmatic patients<br><br>-Mean age 27.2 y<br><br>-N =17 (10 phase I, 7 phase II)<br><br>-23.5% male | Randomized , double-blind, placebo-controlled study, 2 phases | - Atopic asthma<br><br>-Nonsmokers<br><br>-FEV 1 >75% predicted.<br><br>-withheld CS, theophylline, or sodium cromoglycate for 8 weeks.<br><br>-Bronchodilators were withheld for 8 hours before each visit.<br><br>-No RTI or asthma exacerbation at previous 4 weeks. | -Once<br><br>-phase 1:<br><br>Provocation test carried at 2, 5,10,15 minutes intervals with doubling the allergen dose till reach 20% fall in FEV1% from baseline.<br><br>-Phase2: 3 visits at 15, 60, 180 before provocation test respectively. | inhaled heparin (15,000 units USP/ml, total 40.000 units)<br><br>-Device: Inspiron mini nebulizer driven by compressed air at 8 L 'minute. and inhaled to dryness by deep tidal breathing over a 7_ to 9-minute period. | Placebo (N.S) | Phase I: AMP or Methacholine, Phase II: AMP | -Phase I:<br><br>Heparin causes significant increase in methacholine dose response curve for 8/10patients.<br><br>Heparin cause significant effect in preventing the fall of the AMP provoked FEV1, affords 2.4 (0.9 – 4.2) folds protection of AMP produced bronchoconstriction.<br><br>-Phase II:<br><br>Heparin effect on AMP produced bronchoconstriction depends on time:<br><br>At 15 minutes weak effect.<br><br>At 60minutes significant effect<br><br>At 180 minutes no effect. |

| | | | | | | | | | |
|---|---|---|---|---|---|---|---|---|---|
| | | | | | | | | | -Side effects: not reported |
| 7 | Shute, 2017, UK | -Adults<br>-Moderate to severe COPD patients.<br>-Mean age 72 y<br>-N= 40<br>-67.5% male | RCT, double blind, 3 parallel groups 1:1:1, placebo controlled | - COPD patients (stage II to IV according to GOLD2014)<br>-Current or ex-smokers' patients with a smoking history of at least 20 pack years.<br>- No hemoptysis, bleeding or preexisting heparin induced antibodies. | -Twice daily regimen for 21 days of either 2 different UFH doses or placebo.<br>-Besides concomitant twice daily medications (nebulized salbutamol and beclomethasone dipropionate) and pulmonary rehabilitation | -UFH (75.000IU or 150.000 IU)<br>-Nebulized from a jet nebulizer (particles having a mass median aerodynamic diameter (MMAD) of 3.25 µm, and a fill volume of 7 ml) | Placebo (distilled water) | --------------- | - Higher and lower dose heparin significantly ($P<0.05$) increased the FEV1 and FVC change from baseline during the study period specially at day 7, compared with placebo treated patients, and this effect was sustained and even greater at the follow-up visit in the higher dose UFH ( day 21, $P>0.05$) while for lower dose this effect decreases at days 14,28.<br>- Inhaled heparin significantly ($P<0.05$) increased the 6MWD, improves post exercise dyspnea.<br>- UFH doses did not induce any serious |

| | | | | | | | | | |
|---|---|---|---|---|---|---|---|---|---|
| | | | | | | | | | adverse events. No difference at either dose or the placebo group (P>0.05). |
| 8 | Tranfa, 2000, Italy | -Adults<br>-Atopic asthmatic patients.<br>-N= 8<br>-37.5% male | RCT, double blind, cross over | -Nonsmoker<br>-Stable mild - moderate asthma.<br>- hyperresponsiveness to UNDW.<br>-baseline FEV1% at least 80% predicted<br>- No recent history of respiratory tract infection or relevant allergen – exposure within 4 weeks<br>-No use of anti-asthmatic drugs except on demand inhaler and kstopped 24 hours before study. | -Once<br>-45 minutes before UNDW challenge test. | -UFH (1000 iu/ KG) maximum 60,000 IU<br>- Constant flow aerosol through De Vilbiss 65 electronic nebulizer. | Placebo | UNDW (2,4,8,16 ml) till FEV1 fall more than 40% from baseline. | -Inhaled heparin shows significant protective effect than placebo in all UNDW doses ($p<0.05$ after 2 ml, $p<0.01$ after 4,8,16 ml)<br>-Inhaled heparin was well tolerated, and no adverse events reported |
| 9 | Diamant,1996, Uk | -Adults<br>-Atopic mild to moderate asthmatic | RCT, double blind, cross over, two-periods | -Nonsmoking with stable asthma.<br>-Documented EAR (fall in FEV1 >= 20% from baseline, between zero and 3 | -5 doses<br>- 30, 90 minutes before and 2,4,6 hours after the | UFH (1000 iu/ KG) maximum 80,000 IU per dose. | placebo | Histamine inhalation challenge test | -Heparin attenuated EAR $AUC_{0-3}$ response by an average 40% than placebo (p= 0.08). |

| | | | | | | | | | |
|---|---|---|---|---|---|---|---|---|---|
| | | patients<br><br>-Mean age 27.4 y<br><br>-N = 8<br><br>-100% male | | h post allergen) and LAR (fall in FEV1 >= 15% from baseline between 3 and 7 h post allergen) to inhaled house-dust mite extract in the screening period, and that the FEV1 remained stable (± 10% from baseline) for 7 h after inhalation of the diluent of allergen | challenge test. | | | | -Heparin significantly decreased the LAR $AUC_{3-10}$ by 36% than placebo (p=0.005).<br><br>- Inhaled heparin was well tolerated with no reported side effects for all participants during study period. |
| 10 | Garrigo,1996, USA | -Adults<br><br>- Patients with EIA history.<br><br>-Mean age 25 y<br><br>-N= 9<br><br>-66.6% male | Single blind randomized crossover design, 10 different experiment al days. | -Asymptomatic.<br><br>-Nonsmokers.<br><br>-No history of heart disease.<br><br>-No recent history of RTI.<br><br>-No use of anti-asthma medicines except on need B2-agonist inhalers. (Withheld for 24 h before each study day). | -Once<br><br>-Measurement time intervals (15min, 1h, 3h pretreatment with either heparin, cromolyn or placebo) | Undiluted heparin sodium in a concentration of 80,000USP units + benzyl alcohol.<br><br>-Constant flow aerosol during tidal breathing.<br><br>-Device: disposable raindrop medication nebulizer (Puritan Bennett, Lenexa, KS) | Placebo (water contain benzyl alcohol as bacteriostat agent).<br><br>Or<br><br>Cromolyn sodium solution (20mg) + benzyl alohol. | Exercise induced asthma challenge (10 minutes exercise at trade mail). | -Inhaled heparin prevented exercise-induced decreases in SGaw in a time-dependent fashion. The mean ± SE maximum decreases in SGaw after exercise were 16 ± 4.3070 (58% protection), 8 ± 3.5% (78% protection), and 12 ± 2.9% (67% protection), when exercise challenge was performed at 15 min, 1 h, and 3 h after pretreatment with heparin, respectively (n = 9). At all pretreatment |

| | | | | | | | | | |
|---|---|---|---|---|---|---|---|---|---|
| | | | | | | | | | intervals (except 6 h) heparin significantly attenuated the exercise-induced decreases in SGaw ($p < 0.05$); the maximum protection was observed at 1 h after pretreatment with heparin.<br><br>-Side effects: Not reported |
| 11 | Motamed, 2021, Iran | -Adults<br><br>-Mild to moderate asthmatic patients.<br><br>-Mean age 46.4 y<br><br>-N= 70<br><br>-51.4% male | Single blind randomized control trial, two parallel groups). | -Possible acute asthma attack based on initial FEV1, PEFR, on minute zero of the study.<br><br>-No bronchodilators 6 h before ED admission.<br><br>- Able to perform peak flow measurements.<br><br>-No underlying pulmonary or cardiac diseases | -3 doses, with 20 minutes intervals. | - 2.5 mg of albuterol with LMWH (1 mg/kg)<br><br>-Device:<br><br>compressor nebulizer (PulmoMate Compressor Nebulizer 4650D, DeVilbiss Health Care Company, United States) administered via aerosol mask | Albuterol (2.5mg) alone | ----------------------- | -The study suggests that LMWH in mild-moderate asthma attacks may be beneficial in the short term and could be prescribed in addition to standard albuterol therapy.<br><br>the mean PEFR at 40 min was higher in the LMWH group than the control group (202.51 L/min and 180.2 L/min) ($p = 0.001$). Moreover, this |

| | | | | | | | | | |
|---|---|---|---|---|---|---|---|---|---|
| | | | | – No pregnancy.<br><br>-No clotting disorders or treated with anticoagulants. - Not critically ill.<br><br>-Nonsmokers<br><br>-No corticosteroids used medications. | | | | | difference remains significant in the 60th minute (p < 0.001). Further, FEV1 was significantly higher in the LMWH group after 60 min (1.82 L/min vs 1.48 L/min, p < 0.001). Moreover, we found that the hemodynamic parameters were sustainable in the intervention group. |
| 12 (a) | Fal, 2004, Poland | -Adults<br><br>-Asthmatic patients<br><br>-N= 24 | Quasi experiment, No control group. (before &after) | Asthmatic patients | Twice daily for 14 days | Nebulized LMWH 5-10 KU | LMWH is an add-on drug to their asthma medication. | ----------------- | -Statistically significant increase in FEV1% (+15.69 from baseline value, p=0,0049).<br><br>-Statistically significant decrease in the percentage of eosinophils and lymphocytes in BAL, from 4,86 ± 3,48% to 1,25 ± 2,76%; p=0,0006 and from 5,39 ± 2,25% to 2,94 ± 1,23%; p=0,0209, respectively<br><br>-A drop of EG2 in |

| | | | | | | | | | |
|---|---|---|---|---|---|---|---|---|---|
| | | | | | | | | | **BAL supernatant p=0,0493, a slight non-significant decrease in sVCAM1 concentration.**<br>**-No changes in IL-5 or ECP concentrations in serum.** |
| **13** | **Tutluoglu, 2001, Turkey** | **-Adults**<br>**-Allergic asthmatics**<br>**-N= 38**<br>**-31.75% male** | **RCT, double-blind, placebo-controlled study** | **-Asthmatic patients who showed ≥10% decrease in FEV1 during preliminary KCL challenge test**<br>**-Nonsmokers.**<br>**-No pregnancy.**<br>**-No associated diseases (pulmonary, cardiac, renal, hepatic, or diabetic).** | **-Once**<br>**-20 minutes before KCl challenging test.** | **-Inhaled heparin 1000 units/kg**<br>**-Device: a compressor nebulizer (Aerofamily, Medel, Italy). The compressor had**<br>**a maximal pressure of 1.8 bar and the nebulization rate was 0.25**<br>**mL/min.** | **Placebo (normal saline).** | **Hypertonic potassium chloride.** | **No significant difference between EG2, IL-5, ECP two groups regarding basal RFT while for Posttreatment RFT parameters were significantly lower in the normal saline group when compared with the heparin group**<br>**($p < 0.001$).**<br>**specially in day 2, There was no observed decrease in RFT for heparin group.**<br>**Side effects: Not mentioned** |

| | | | | | | | | | |
|---|---|---|---|---|---|---|---|---|---|
| 14 | Ashoor,2020, Egypt | -Adults<br><br>-Stage II to IV COPD<br><br>mechanicall y ventilated.<br><br>-Mean age 45.8 y<br><br>-N= 60<br><br>-73.3% male | RCT, double-blind, two parallel groups. | -COPD exacerbation with primary respiratory failure.<br><br>-body mass index of ≤ 40 kg/m2 with<br><br>-Mechanically ventilated for more than 24 h<br><br>-Nonpregnant.<br><br>-No history of ischemic heart disease, pulmonary bleeding (within the previous three months), bleeding diathesis.<br><br>- No allergy to heparin, or history of heparin-induced thrombocytopenia (HIT). | Every 6 hours for fourteen days.<br><br>Device: nebulization chamber (Ameco Technology; particle size: 0.5–10 µm, nebulization rate: > 0.3 ml/min) connected to the inspiratory limb of the breathing circuit 15 cm from the Y of the circuit. | Nebulized heparin (25,000 IU) and salbutamol (5 mg) . | Salbutamol only | ------------- | Patients in the Group HS had significantly more ventilator free days (4.7 ± 3.3) compared with those in the Group S (2.4 ± 2.6), P = 0.007. PaCO2 levels, PaO2/FiO2, the decrease in the CRP level and the increase in the APTT from the baseline showed no evidence of difference in both groups.<br><br>The co-administration of nebulized heparin and salbutamol, compared with salbutamol alone, significantly increased (VFDs) among mechanically ventilated AECOPD patients without increasing bleeding risks. |

| | | | | | | | | | |
|---|---|---|---|---|---|---|---|---|---|
| 15 | Tahir, 1999, USA | -Adults<br><br>-Exercise-induced asthmatic patients.<br><br>-N= 13<br><br>-30.7% male | RCT, double-blind, cross over, 7 different experimental days. | -Nonsmokers<br><br>-Asymptomatic at the time of the study<br><br>-No history of bleeding diathesis, heart disease, or a recent RTI). Subjects with a 15% decrease in FEV1 after 10 min treadmill exercise were included in the study. | -once<br><br>-45 minutes before the exercise challenge. | -Enoxaparin 0.5 mg/kg,.<br><br>-Enoxaparin 1 mg/kg<br><br>- Enoxaparin 2 mg/kg<br><br>-Heparin undiluted 20,000 USP U/ml.<br><br>- Constant-flow aerosol during tidal breathing<br><br>Device: disposable raindrop medication nebulizer<br><br>(Puritan Bennett, Lenexa,KS) during a 15- to 20-min period | Placebo (vehicle, bacteriostatic injection water) | -Exercise testing was performed on a treadmill.<br><br>-Methacholine provocation test (5 subjects only with enoxaparin 2 mg/kg or placebo) | -The exercise-induced decreases in FEV1 were inhibited by 31% with heparin and by 28%, 38%, and 48% by enoxaparin at doses of 0.5,1,2 mg/kg ($p<0.05$).<br><br>-In EIB, The inhibitory effect of 0.5 mg/kg dose of enoxaparin was comparable to heparin (7.5 mg/kg), whereas 2 mg/kg dose of enoxaparin was the most potent.<br><br>-Inhaled enoxaparin failed to modify the bronchoconstrictor response to methacholine, and did not change the plasma antifactor Xa activity. |
| 16 | Ahmed, 1993, | -Adults | -Single blind | -Nonsmoker | - 45 minutes | -Inhaled heparin | -placebo | -------------------- | -Inhaled heparin |

| | | | | | | | | | |
|---|---|---|---|---|---|---|---|---|---|
| | USA | -Exercise induced asthma.<br>-N= 12<br>-66.6% male | randomized crossover. | -No history of eart disease or recent RTI.<br>-No asthma treatment at the previous 24 hours. | before exercise. | 1000 U/kg.<br>-Device: disposable raindrop medication nebulizer<br>(Puritan Bennett, Lenexa,KS) during a 15- to 20-min period | Or cromolyn sodium 20 mg | | prevent exercise induced asthma without influencing histamin induced bronchoconstrictio n.<br>-Inhaled heparin didn't affect partial thromboplastin time. |
| 17<br>(a) | Abd-Elaty, 2007, Egypt | -Adults<br>-Acute asthmatic exacerbatio n<br>-Mean age 31 y<br>-N= 30<br>-66.6% male | RCT, double blind, placebo-controlled | -Moderate severity of asthma exacerbation. | - Every 4 hr. and the placebo group received 0.9% saline solution for 24 hours.<br>-Measurement of peak flow rate after 24hours | -Inhaled heparin therapy (20,000 U in 4 mL) | -Placebo (0.9% saline)<br>-Both groups receive IV hydrocortison e and inhaled salbutamol | ------------------ | All patients in both groups showed improvement in oxygen saturations, respiratory rates, and peak flow rates. Statistically significant difference was observed between the 2 groups regarding both the respiratory parameters and the mean number of salbutamol nebulization's needed (P < 0.05, P < 0.01). |

| | | | | | | | | | |
|---|---|---|---|---|---|---|---|---|---|
| **18**<br><br>**(a)** | **Ceyhan, 2000, Turkey** | **-Adults**<br><br>**-Mild asthmatic patients.**<br><br>**-Mean age 33 y**<br><br>**-N= 15**<br><br>**-46.7% male** | **RCT, single-blind, crossover, placebo-controlled.** | **-Mild asthmatic patients** | **-Once**<br><br>**-45 minutes before the provocation test.** | **-Aerosolized standard heparin (1.000 U/kg) or aerosolized LMWH (Enoxaparin, 0.8 mg/kg).** | **Placebo (saline 0.9%)** | **Methacholine bronchial provocation test.** | **-Heparin and LMWH cause a statistically significant increase in the geometric mean log PD20 values of methacholine than placebo (p < 0.0009).**<br><br>**-Therefore, both standard heparin and LMWH can reduce Methacholine induced bronchoconstrictio n.** |
| **19**<br><br>**(a)** | **Ceyhan, 1997, Turkey** | **-Adults**<br><br>**-Mild asthmatic patients.**<br><br>**-N= 15 - 46.6% male** | **RCT, single-blind, crossover, placebo-controlled** | **mild asthmatic patients** | **-Once**<br><br>**-45 minutes before the provocation tests.** | **-Aerosolized heparin inhalation (1,000 U/kg).** | **Placebo (saline 0.9%)** | **Methacholine and adenosine bronchial provocation test.** | **The heparin increased the geometric mean log methacholine PD20 (P < 0.0009), with less attenuation at the adenosine-induced bronchoconstrictio n.** |
| **20**<br>**(a)** | **Kwasniewski, 2000** | **-Adults**<br><br>**-Asthmatic and COPD patients** | **-Not mentioned** | **Adults**<br><br>**Asthmatic patients & COPD.** | **-Twice a day for 3-6 days treatment.** | **-Nadroparin (LMWH) in nebulization as an odd on treatment, 15 kU per dose** | **Ordinary treatment only.** | **………………………** | **-Nadroparin increased serum anti-Xa activity in all subjects. This change was less pronounced in** |

| | | | | | | | | | |
|---|---|---|---|---|---|---|---|---|---|
| | | -N= 21<br><br>-52.4% male | | | | | | | asthmatics but tended to last longer than in controls (p<0.05).<br><br>-No changes in other estimated parameters (e.g., APTT, fibrinogen, platelets) were observed after 8-9 days of treatment. |
| 21<br><br>(a) | Muszyńska, 2002, Poland | -Adults<br><br>-Asthmatic patients<br><br>-N=17 | Quasi experiment (before &after) | Asthmatic patients. | -once daily for one week. | -Inhaled LMWH dose 5000 IU | | ----------------------<br>-- | LMWH changed significantly cell BALF profile, decreased the number of inflammatory cells (p < 0.005) but did not change serum sIL-2R concentrations (p < 0.13). Baseline serum levels of sIL-2R were normal (73.9 +/- 26.6 U/ml) according to producer's norm. |
| 22<br><br>(a) | Hong, 1996 | -Children<br><br>-Asthmatic patients<br><br>-N=30 | -Cross-over design of 2 groups (interventio n and control) | Asthmatic children | Not mentioned | Heparin inhalation + routine treatment | Routine treatment only | -------------- | The effective rate of the treatment group is 96.67% obviously higher than the control group's (70.00%), P |

| | | | | | | | | | |
|---|---|---|---|---|---|---|---|---|---|
| | | | | | | | | | < 0.01.<br><br>No side effects were seen in the treatment group. |
| 23 (a) | Stelmach, 2001, Poland | -Children<br><br>-Mild atopic asthma.<br><br>-N=15 | RCT, double-blind, cross-over, placebo-controlled. | Children with mild atopic asthma | A single dose before the provocation test. | Heparin inhalation | Placebo | Methacholine | A single dose of inhaled heparin significantly decreased bronchial hyperresponsiveness to methacholine when compared to a placebo.<br><br>Side effects: not mentioned |
| 24 (a) | Stelmach, 2002, Poland | -Children<br><br>-Mild asthmatic patients.<br><br>-N=14 | RCT, double-blind, cross-over, placebo-controlled. | children with a typical history of mild atopic asthma | A single dose before the provocation test. | Heparin inhalation | Placebo | leukotriene D3 | A single dose of inhaled heparin significantly decreased bronchial hyperreactivity to leukotriene in children with mild asthma (p = 0.005). PC20L after heparin inhalation increased in eight patients and decreased in two.<br><br>Side effects: not mentioned |

| | | | | | | | | | |
|---|---|---|---|---|---|---|---|---|---|
| 25 (a) | Stelmach, 2003, Poland | -Children<br><br>-Mild asthmatic patients<br><br>-N= 23 | RCT, double-blind, cross-over, placebo-controlled | Children with a typical history of mild allergic asthma. | A single dose before the provocation test. | Heparin inhalation | Placebo | histamine or leukotriene D4 | A single dose of inhaled heparin significantly decreased bronchial hyperreactivity to histamine and leukotriene in children with mild asthma.<br><br>Side effects : not mentioned |
| 26 (a) | Lui, 2003, China | -Adults<br><br>-Asthmatic patients<br><br>-N= 40 | RCT, single-blind, auto controlled | Mild-moderate asthmatic patients | | heparin spray | Placebo | Methacholine | -Decreased ECP, TNF-α and sIL-2R levels in sputum of heparin group (P<0.05);<br><br>-$FEV_1$, PEFR and FVC increased in heparin group (P<0.05)<br><br>-There was a significant increase in PC20 Methacholine value in the heparin group (P<0.05).<br><br>Side effects: not mentioned |
| (a): abstract N.S: Normal saline FEV1: Forced expiratory volume at one second. ATP : adenosine triphosphateTCC: total cell count | | | | | | | | | |

# Appendix 6

# PRISMA checklist

| Section and Topic | Item # | Checklist item | Location where item is reported |
|---|---|---|---|
| **TITLE** | | | |
| Title | 1 | Identify the report as a systematic review. | P1 |
| **ABSTRACT** | | | |
| Abstract | 2 | See the PRISMA 2020 for Abstracts checklist. | P2 |
| **INTRODUCTION** | | | |
| Rationale | 3 | Describe the rationale for the review in the context of existing knowledge. | P3 |
| Objectives | 4 | Provide an explicit statement of the objective(s) or question(s) the review addresses. | P4 |
| **METHODS** | | | |
| Eligibility criteria | 5 | Specify the inclusion and exclusion criteria for the review and how studies were grouped for the syntheses. | P4-5 |
| Information sources | 6 | Specify all databases, registers, websites, organisations, reference lists and other sources searched or consulted to identify studies. Specify the date when each source was last searched or consulted. | P5 |
| Search strategy | 7 | Present the full search strategies for all databases, registers and websites, including any filters and limits used. | P6 |
| Selection process | 8 | Specify the methods used to decide whether a study met the inclusion criteria of the review, including how many reviewers screened each record and each report retrieved, whether they worked independently, and if applicable, details of automation tools used in the process. | P6 |
| Data collection process | 9 | Specify the methods used to collect data from reports, including how many reviewers collected data from each report, whether they worked independently, any processes for obtaining or confirming data from study investigators, and if applicable, details of automation tools used in the process. | P6 |
| Data items | 10a | List and define all outcomes for which data were sought. Specify whether all results that were compatible with each outcome domain in each study were sought (e.g. for all measures, time points, analyses), and if not, the methods used to decide which results to collect. | P6 |
| | 10b | List and define all other variables for which data were sought (e.g. participant and intervention characteristics, funding sources). Describe any assumptions made about any missing or unclear information. | P6 |
| Study risk of bias assessment | 11 | Specify the methods used to assess risk of bias in the included studies, including details of the tool(s) used, how many reviewers assessed each study and whether they worked independently, and if applicable, details of automation tools used in the process. | P7 |
| Effect | 12 | Specify for each outcome the effect measure(s) (e.g. risk ratio, mean difference) used in the synthesis or presentation of | P7p7 |

| Section and Topic | Item # | Checklist item | Location where item is reported |
|---|---|---|---|
| measures | | results. | |
| Synthesis methods | 13a | Describe the processes used to decide which studies were eligible for each synthesis (e.g. tabulating the study intervention characteristics and comparing against the planned groups for each synthesis (item #5)). | P7 |
| | 13b | Describe any methods required to prepare the data for presentation or synthesis, such as handling of missing summary statistics, or data conversions. | P7 |
| | 13c | Describe any methods used to tabulate or visually display results of individual studies and syntheses. | P7 |
| | 13d | Describe any methods used to synthesize results and provide a rationale for the choice(s). If meta-analysis was performed, describe the model(s), method(s) to identify the presence and extent of statistical heterogeneity, and software package(s) used. | P7 |
| | 13e | Describe any methods used to explore possible causes of heterogeneity among study results (e.g. subgroup analysis, meta-regression). | P7 |
| | 13f | Describe any sensitivity analyses conducted to assess robustness of the synthesized results. | P8 |
| Reporting bias assessment | 14 | Describe any methods used to assess risk of bias due to missing results in a synthesis (arising from reporting biases). | P7 |
| Certainty assessment | 15 | Describe any methods used to assess certainty (or confidence) in the body of evidence for an outcome. | P8 |
| **RESULTS** | | | |
| Study selection | 16a | Describe the results of the search and selection process, from the number of records identified in the search to the number of studies included in the review, ideally using a flow diagram. | P9, P26 |
| | 16b | Cite studies that might appear to meet the inclusion criteria, but which were excluded, and explain why they were excluded. | P 23-25 |
| Study characteristics | 17 | Cite each included study and present its characteristics. | P 23-25 |
| Risk of bias in studies | 18 | Present assessments of risk of bias for each included study. | P9, P27 |
| Results of individual studies | 19 | For all outcomes, present, for each study: (a) summary statistics for each group (where appropriate) and (b) an effect estimate and its precision (e.g. confidence/credible interval), ideally using structured tables or plots. | P27-28 Appendix 2 |
| Results of syntheses | 20a | For each synthesis, briefly summarise the characteristics and risk of bias among contributing studies. | P 9-13 |
| | 20b | Present results of all statistical syntheses conducted. If meta-analysis was done, present for each the summary estimate and its precision (e.g. confidence/credible interval) and measures of statistical heterogeneity. If comparing groups, describe the direction of the effect. | P 9-13 |
| | 20c | Present results of all investigations of possible causes of heterogeneity among study results. | P 9-13 |

| Section and Topic | Item # | Checklist item | Location where item is reported |
|---|---|---|---|
| | 20d | Present results of all sensitivity analyses conducted to assess the robustness of the synthesized results. | P 9-13 |
| Reporting biases | 21 | Present assessments of risk of bias due to missing results (arising from reporting biases) for each synthesis assessed. | P13, appendix 2 |
| Certainty of evidence | 22 | Present assessments of certainty (or confidence) in the body of evidence for each outcome assessed. | Appendix 4 |
| **DISCUSSION** | | | |
| Discussion | 23a | Provide a general interpretation of the results in the context of other evidence. | P14 |
| | 23b | Discuss any limitations of the evidence included in the review. | P15 |
| | 23c | Discuss any limitations of the review processes used. | P15 |
| | 23d | Discuss implications of the results for practice, policy, and future research. | P15 |
| **OTHER INFORMATION** | | | |
| Registration and protocol | 24a | Provide registration information for the review, including register name and registration number, or state that the review was not registered. | P4 |
| | 24b | Indicate where the review protocol can be accessed, or state that a protocol was not prepared. | P4 |
| | 24c | Describe and explain any amendments to information provided at registration or in the protocol. | P4 |
| Support | 25 | Describe sources of financial or non-financial support for the review, and the role of the funders or sponsors in the review. | P15 |
| Competing interests | 26 | Declare any competing interests of review authors. | P15 |
| Availability of data, code and other materials | 27 | Report which of the following are publicly available and where they can be found: template data collection forms; data extracted from included studies; data used for all analyses; analytic code; any other materials used in the review. | P15 |

*From:* Page MJ, McKenzie JE, Bossuyt PM, Boutron I, Hoffmann TC, Mulrow CD, et al. The PRISMA 2020 statement: an updated guideline for reporting systematic reviews. BMJ 2021;372:n71. doi: 10.1136/bmj.n71

For more information, visit: http://www.prisma-statement.org/
